# Phylogenetic Distribution and Evolution of Type VI Secretion System in the Genus *Xanthomonas*

**DOI:** 10.3389/fmicb.2022.840308

**Published:** 2022-04-14

**Authors:** Prabha Liyanapathiranage, Naama Wagner, Oren Avram, Tal Pupko, Neha Potnis

**Affiliations:** ^1^Department of Entomology and Plant Pathology, Auburn University, Auburn, AL, United States; ^2^The Shmunis School of Biomedicine and Cancer Research, George S. Wise Faculty of Life Sciences, Tel Aviv University, Tel Aviv, Israel

**Keywords:** T6SS, *Xanthomonas*, evolution, non-pathogenic, phylogenetics

## Abstract

The type VI secretion system (T6SS) present in many Gram-negative bacteria is a contact-dependent apparatus that can directly deliver secreted effectors or toxins into diverse neighboring cellular targets including both prokaryotic and eukaryotic organisms. Recent reverse genetics studies with T6 core gene loci have indicated the importance of functional T6SS toward overall competitive fitness in various pathogenic *Xanthomonas* spp. To understand the contribution of T6SS toward ecology and evolution of *Xanthomonas* spp., we explored the distribution of the three distinguishable T6SS clusters, i3*, i3***, and i4, in approximately 1,740 *Xanthomonas* genomes, along with their conservation, genetic organization, and their evolutionary patterns in this genus. Screening genomes for core genes of each T6 cluster indicated that 40% of the sequenced strains possess two T6 clusters, with combinations of i3*** and i3* or i3*** and i4. A few strains of *Xanthomonas citri*, *Xanthomonas phaseoli*, and *Xanthomonas cissicola* were the exception, possessing a unique combination of i3* and i4. The findings also indicated clade-specific distribution of T6SS clusters. Phylogenetic analysis demonstrated that T6SS clusters i3* and i3*** were probably acquired by the ancestor of the genus *Xanthomonas*, followed by gain or loss of individual clusters upon diversification into subsequent clades. T6 i4 cluster has been acquired in recent independent events by group 2 xanthomonads followed by its spread via horizontal dissemination across distinct clades across groups 1 and 2 xanthomonads. We also noted reshuffling of the entire core T6 loci, as well as T6SS spike complex components, *hcp* and *vgrG*, among different species. Our findings indicate that gain or loss events of specific T6SS clusters across *Xanthomonas* phylogeny have not been random.

## Introduction

The genus *Xanthomonas* belongs to the gamma subdivision of the phylum Proteobacteria (Jun et al., 2010). It consists of more than 35 species, some of which cause economically important diseases in more than 400 host plants (Timilsina et al., 2020). Even though a wide host range is observed with *Xanthomonas* spp., individual strains can be tightly restricted to a particular host or host group. Hence, the *Xanthomonas* spp. are further differentiated into pathovars based on this characteristic (White et al., 2009; An et al., 2020). To date, *Xanthomonas* spp. have been isolated from host plants belonging to 124 monocotyledonous and 268 dicotyledonous species (White et al., 2009; Kyrova et al., 2020). In terms of host tissue specificity, *Xanthomonas* spp. can either invade the vascular tissues or survive in the intercellular spaces of the mesophyll parenchyma tissue (An et al., 2020). To successfully adapt to the hostile host environment, *Xanthomonas* spp. usually depend on several factors that are important for their invasion. These factors are responsible for various functions, including adherence to the plant surface, invasion to the intercellular space of the host tissue, manipulation of plant cellular processes, acquisition of nutrients, counteracting plant defense responses, and competing with other microbes that occupy the same niche (Büttner and Bonas, 2010). Identification of the adaptation factors that contribute to host–pathogen interactions is crucial in implementing future management strategies.

To date, more than 1,700 *Xanthomonas* genomes are publicly available in the National Center for Biotechnology Information (NCBI) database. With the availability of such a large amount of genomic data, comparative genomic studies enabled the identification of many potential virulence and fitness strategies contributing to the adaptation to a variety of monocot/dicot hosts and the establishment of successful vascular or non-vascular modes of infection (Jacques et al., 2016; An et al., 2020). These strategies include various secretion systems, by which pathogenic bacteria translocate proteins/DNA directly into the host cell or into the extracellular milieu.

In Gram-negative bacteria, 10 different secretion systems have been identified, but only six of them (I–VI) have been described in the genus *Xanthomonas* (Meuskens et al., 2019; Alvarez-Martinez et al., 2020; Palmer et al., 2020). Of these six systems, types II, III, and IV have been extensively studied in the genus *Xanthomonas*. In contrast, the role, distribution, and evolutionary history of the most recently identified type VI secretion system (T6SS) has been described only in few studies (Bayer-Santos et al., 2018; Bansal et al., 2019, 2020; Bayer-Santos et al., 2019; Ceseti et al., 2019; Alvarez-Martinez et al., 2020; Choi et al., 2020; Zhu et al., 2020; Montenegro Benavides et al., 2021). Most of the aforementioned studies used only representative *Xanthomonas* spp., to derive conclusions regarding the distribution of the T6SS clusters in the whole genus *Xanthomonas*. Yet, the evolutionary history of multiple T6SS clusters, their distribution across the genus, and their association with particular pathogen lifestyle all remain unclear.

T6SS is present in approximately 25% of Gram-negative bacteria, mainly within the phylum Proteobacteria and the classes α-, β-, and γ-proteobacteria, including pathogenic, beneficial, and commensal bacteria, indicating possible functions of this system unrelated to pathogenicity (Boyer et al., 2009; Durand et al., 2014). T6SS is composed of 15 to 23 different proteins, including 13 core structural proteins that assemble into an injectisome-like structure (Shyntum et al., 2014). Core components are named as Tss (for type VI secretion) A-M and are usually encoded in the same gene cluster (Zoued et al., 2014). In addition to the core genes, most T6 clusters also include Tag (type VI–associated genes), which encode accessory proteins, suggested to be important for the transcriptional or posttranscriptional regulation and assembly of the T6SS. Finally, T6SS includes T6 effectors and their associated immunity proteins (Shalom et al., 2007; Boyer et al., 2009; Silverman et al., 2011; Coulthurst, 2013). This Tss nomenclature is now widely adopted by many, while common names, such as Hcp, VgrG, and ClpV, are also in use.

T6SS is comprised of three major complexes: the transmembrane complex, the baseplate, and the tail. Together, they form a larger machine that extends from the bacterial cytoplasm, across the bacterial cell wall and into the target cell (Silverman et al., 2012; Coulthurst, 2013; Durand et al., 2015; Nguyen et al., 2018; and Hernandez et al., 2020). The transmembrane complex made from TssM, TssJ, and TssL acts as a docking site for the cytoplasmic baseplate-like structure made from TssE, TssF, TssG, and TssK. It also serves as an adaptor between the transmembrane complex and the tail. The internal tube of the tail component is made of Hcp tubes that are assembled into the central VgrG–PAAR spike and is enclosed by a contractile sheath composed of TssB and TssC. Secretion of effector proteins through the T6SS is a one-step process, independent of the Sec or Tat secretion machinery, which is mediated through contraction of the sheath to propel the Hcp–VgrG–PAAR complex toward the target cell and deliver a cocktail of effectors in each contraction. Unlike other secretion systems, after each firing event, the T6 tube disassembles, and Hcp is lost to the extracellular milieu. AAA+ClpV (TssH) ATPase is recruited to the contracted sheath to disassemble the complex and to recycle the sheath components for the next round of firing. Apart from being core components of the T6SS, Hcp (hemolysin coregulated protein) and VgrG (valine-glycine repeat protein G) have also been identified as specialized effectors important in antibacterial activity in several pathogen systems (Lien and Lai, 2017; Ma et al., 2017). Effectors that get delivered via the T6SS are categorized as either “cargo” or “specialized.” Cargo effectors interact with the Hcp–VgrG–PAAR complex non-covalently, whereas specialized effectors are covalently linked to these proteins. T6 effectors and their antitoxins or immunity proteins may be encoded either within the T6 gene cluster, which encodes the core genes, or scattered in other parts of the bacterial genome (Hernandez et al., 2020).

Depending on the 13 core proteins, T6 clusters have been separated into four subtypes (T6SS^i–iv^). The majority of the proteobacteria have T6 clusters belonging to the subtype T6SS^i^, and because of the diversity observed in this subgroup, it has been further subdivided into five phylogenetic clades (i1–i5) (Boyer et al., 2009). Bacteria belonging to the family Xanthomonadales harbor three such clades: i1, i3, and i4, whereas bacteria within genus *Xanthomonas* harbor only the i3 and i4 clades (Bayer-Santos et al., 2019; Alvarez-Martinez et al., 2020). Clade i3 was further subdivided into distinct subclades: i3*, i3**, and i3*** based on the phylogenetic analysis conducted using tssC gene (Bayer-Santos et al., 2019). Species within the genus *Xanthomonas* harbor subclades i3* (e.g., *Xanthomonas citri*), i3*** (e.g., *Xanthomonas oryzae*), or both i3* and i3*** (e.g., *Xanthomonas perforans*), but not i3**.

The phylogenetic-based division to clades and subclades is also reflected in the set of genes included within the T6SS gene cluster and its genome organization. For example, all *Xanthomonas* species within the i3 clade lack *tssJ*, a core component in the outer membrane complex of T6 machinery (Alvarez-Martinez et al., 2020). In addition, i3* and i3*** differ in their genetic architecture, for example, i3* cluster in *Xanthomonas euvesicatoria* 85-10 is approximately 40 kb, whereas the i3*** cluster is approximately 20 kb. This difference in cluster size is due to the presence of different genes encoding hypothetical proteins and transcriptional regulators in each cluster (Boyer et al., 2009; Abendroth et al., 2017; Alvarez-Martinez et al., 2020).

Similar to observations with other bacteria, some *Xanthomonas* spp. encode multiple T6 copies: either both i3* and i3*** or one copy from each subclade together with the i4 cluster (Bingle et al., 2008; Alvarez-Martinez et al., 2020). These multiple copies of T6 gene clusters are believed to be acquired by independent horizontal gene transfer events, suggesting different roles played by the T6 clusters in different bacteria (Bingle et al., 2008; Blondel et al., 2009; Boyer et al., 2009). In *X. oryzae* pv. oryzae, one T6 cluster is distantly related to the *phaseoli* clade, whereas the second cluster forms a monophyletic clade with i3*** cluster of *X. euvesicatoria* and *X. perforans*, implicating a recent common origin (Montenegro Benavides et al., 2021).

The goal of this study is to harness a large set of publicly available *Xanthomonas* genomes spanning the entire genus *Xanthomonas* with the genomes of multiple important pathogenic and non-pathogenic or commensal species and study the conservation, genetic organization, and evolutionary patterns of multiple T6SS clusters. We hypothesize that the acquisition of the multiple T6SS clusters occurred as independent events during the adaptation of *Xanthomonas* species to specific hosts, as they diverged into various clades. Specific cases of loss or gain events happened in certain clades as an adaptation to vascular or non-vascular lifestyles. We examined the phylogenetic distribution of T6SS clusters and systematically assessed the acquisition and loss events among different species along with the reshuffling of entire loci or individual genes, to obtain insights into the significance of T6SS in host adaptation of xanthomonads.

## Materials and Methods

### *Xanthomonas* Genomes

All *Xanthomonas* RefSeq genomes available in NCBI on January 16, 2022, were downloaded. This included a total of 1,740 genomes of 39 *Xanthomonas* species (see full list of species and accession numbers in Supplementary Table 1). For each of these genomes, protein translation of open reading frames was extracted using Prodigal (Hyatt et al., 2010).

### Initial Dataset of T6 Genes

To find the core genes of T6-i3*, T6-i3***, and T6-i4 clusters in each of the *Xanthomonas* genomes, we first established datasets of core genes of each of the three clusters. The core genes of T6-i3* and T6-i3*** were taken from *X. euvesicatoria* str. 85-10 (accession NC_007508.1), whereas the core genes of T6-i4 were taken from *X. oryzae* pv. oryzae PXO99A (accession NC_010717.2). The core gene loci of all three clusters included TssA, TssB, TssF, TssG, TssK, TssL (T6-i4 included both TssL1 and TssL2), and TssM. T6-i3* and T6-i3*** also included TssC and TssD. T6-i3*** and T6-i4 also included TssE. T6-i3* also included TssH, T6-i3*** also included ImpE-like protein TagF, and T6-i4 also included TssJ and ClpV.

### Identification of T6SS Clusters Across All 1,740 *Xanthomonas* Genomes

The core genes of T6-i3*, T6-i3***, and T6-i4 clusters were searched for in each of the *Xanthomonas* genomes using BLASTx (Altschul et al., 1990). We used the core gene sequences described above as query and the protein sequences of each of the genomes as databases. The commands used were **makeblastdb -in {dataset} -dbtype prot -out {dataset_db}** to create the BLAST database, and **blastx -db {dataset_db} -query {query} -outfmt 6 -out {out}** to run BLASTx. “dataset” was the protein FASTA file of a certain *Xanthomonas* genome, and “query” was a DNA FASTA file containing the core genes of a certain T6 system. It was repeated for each genome and system combination. For each of the core genes in the query, the best BLAST hit in the genome was considered, if it had an E-value lower than 10^−10^ and identity of at least 50% to the core gene, and a coverage of at least 30% (i.e. that the length of the aligned region is at least 30% of the query gene length). In case a certain gene in the genome was found to be a hit matching our criteria for genes of more than one T6 system, for example, TssA of T6-i3* and TssA of T6-i3***, the hit was saved only for the gene of the system it had the highest identity to. ClpV shares domains with ClpB, so to avoid classifying ClpB genes as ClpV; we blasted ClpB versus the genomes, and protein sequences that showed a higher sequence similarity to ClpB than to ClpV were discarded. Then, for each genome, the presence of each T6 system was evaluated. If all the core genes of a specific system were found in the genome, we considered this system to be full in this genome. Otherwise, if it had at least one core gene, we considered it partial. Gene Neighborhood tool was used to identify gene synteny and cluster organization (Chen et al., 2021).

### Construction of T6 Core Gene Phylogenies

Following the identification of core genes, as described previously, the corresponding protein sequences of each gene were aligned using MAFFT (v.7.471) with the L-ins-I option (Katoh et al., 2002). Phylogenetic trees under the maximum likelihood (ML) criterion were reconstructed using the IQ-TREE multicore version 2.1.2 COVID edition (Minh et al., 2020). The “–m MFP” function was used for the model selection, whereas “–merge rclusterf” was used for partition finding. Branch support was determined using 1,000 bootstraps and 1,000 SH-aLRT bootstrap replicates (-B 1000, -alrt 1000). Nodes with ≥80% support with the SH-aLRT and >80% with normal bootstrap supports were considered well supported (Davis et al., 2021). Trees were visualized using FigTree v1.4.4 (Rambaut et al., 2018).

### SplitsTree Analysis for Homologous Recombination Visualization

To identify and visualize possible conflicting signals, which would suggest possible recombination events and evolutionary relationships within sequence data, phylogenetic networks were inferred using SplitsTree4 (version 4.17.0) (Huson and Bryant, 2006). Concatenated T6 core genes from each cluster were used to generate SplitsTree files, for a selected subset of genomes using the automated multilocus sequence analysis pipeline (automlsa2) (Davis et al., 2021). Resultant splits.nex files were visualized using the SplitsTree4 (version 4.17.0), with the rooted equal angle algorithm (Gambette and Huson, 2008). Recombination events were identified by the branches that form parallelograms (Joseph and Forsythe, 2012).

### Multilocus Sequence Analysis

To reconstruct the species phylogeny, we conducted a multilocus sequence analysis (MLSA) using 12 housekeeping genes: *atpD* (ATP synthase β chain), *dnaK* (70-kDa heat shock protein), *efp* (elongation factor P), *fusA* (translation elongation factor aEF-2), *fyuA* (transmembrane protein; Ton-B–dependent transporter), *gapA* (glyceraldehyde 3-phosphate dehydrogenase), *glnA* (glutamine synthetase I), *gltA* (citrate (Si)-synthase gene), *gyrB* (DNA gyrase β subunit), *lacF* (sugar ABC transporter permease), *lepA* (GTP-binding protein), and *rpoD* (RNA polymerase sigma 70 factor). These genes were previously used for MLSA (Young et al., 2008; Fischer-Le Saux et al., 2015; Timilsina et al., 2015). We created a multiple sequence alignment (MSA) for each of these 12 genes using MAFFT (Katoh et al., 2002; Katoh and Standley, 2013) and then concatenated these MSAs. The concatenated MSA was used as input for RaxML (Stamatakis, 2014) to create the phylogenetic tree.

### Inferring Evolutionary Gain/Loss Events of T6SS Clusters Across *Xanthomonas* Genus Phylogeny

We used GLOOME to analyze the gain and loss probabilities for three T6 clusters during evolution of the entire *Xanthomonas* genus (Cohen et al., 2010). GLOOME analyzes phyletic patterns based on presence and absence of individual core genes of the cluster and accurately infers branch-specific and site-specific gain and loss events. We generated the input file of phyletic pattern based on the BLASTx results described above, in which “1” indicates presence and “0” indicates absence. The MLSA tree was used as input tree. Cladogram was drawn with FigTree v1.4.4 (Rambaut et al., 2018). Gain and loss events were added manually based on the GLOOME output files.

### Construction of Core Genome Phylogeny

For the reconstruction of the core genome phylogeny Roary was used (Page et al., 2015). All the genomes downloaded from the NCBI GenBank in the fna format were first annotated using the Prokka (v1.14.5) software tool (Seemann, 2014). After annotating the genome to determine the location and attributes of the genes contained in them, the resultant gff file in the GFF3 format was used as input file to perform the pan-genome analysis using Roary. Roary extracted the coding sequences from the input GFF3 files, converted them into protein sequences, removed partial clusters, and created preclusters. Next Roary performed BLASTP with 95% sequence identity, and sequences were clustered with Markov cluster algorithm. As the next step, Roary generated clusters and inferred orthologs. Finally, Roary identified core genes and generated a concatenated core gene alignment that was used to generate maximum likelihood phylogeny using RAxML (v 8.2.12) (Stamatakis, 2014).

## Results

### Distribution of T6SS Clusters in the Genus *Xanthomonas*

In this study, we analyzed 1,740 complete genome sequences, belonging to 39 *Xanthomonas* spp. retrieved from the NCBI RefSeq with a large proportion of them from species, *X. oryzae* (396 genomes), *X. citri* (195 genomes), *X. phaseoli* (97 genomes), *X. perforans* (151 genomes), and *X. arboricola* (136 genomes). This collection of genomes also included commensal *Xanthomonas* spp., isolated from environment (rain) as well as isolated from diverse plant species (Potnis et al., unpublished). Based on core gene sequence identity of T6SS clusters, presence of complete T6 i3*** was identified in 832 genomes; T6 i3* in 710 genomes and 497 genomes had the complete T6 i4 cluster (Table 1). Presence of only a single T6 cluster, T6 i3* was identified in 23% of *Xanthomonas genomes*, the majority belonging to *X. citri*, *X. phaseoli*, *X. vesicatoria*, *X. axonopodis*, and few strains belonging to *X. arboricola*, *X. campestris*, *X. pisi*, *X. dyei*, and *X. cucurbitae*, whereas that of T6SS i3*** alone in *X. vasicola* and that of T6 i4 alone in *X. oryzae*, *X. translucens*, *X. fragarie*, couple of *X. hortorum* strains and *X. bromi*. Some *X. oryzae* strains contain both T6 i3*** and T6 i4. The T6SS makeup in *X. citri* is highly variable, with majority of strains containing T6 i3* alone, 14 strains with T6 i3* and T6 i4, whereas a couple of them lack T6 clusters. *X. phaseoli* also shows variability in genetic makeup of T6 clusters, with 40 strains with T6 i3* alone, 52 strains with T6 i3* and T6 i3***, and 2 strains containing T6 i3* and T6 i4. Eighty-nine percent of the *X. oryzae* strains possess both T6 i3*** and T6 i4, and 2.77% contain T6 i4 alone. *X. euvesicatoria* and *X. perforans* contain both T6 i3* and T6 i3*** clusters. *Xanthomonas albilineans*, *Xanthomonas sacchari*, *X. cannabis*, and majority of *X. arboricola* strains lack T6 cluster. *Xanthomonas maliensis* strains contain all three T6 clusters. Approximately 40% of the currently classified *X. campestris* strains contain either i3* or i3*** complete clusters, with 13% of them containing both i3* and i3*** clusters (Table 1).

**TABLE 1:** Analysis of T6 cluster distribution in genus *Xanthomonas*.

*Xanthomonas* spp.	Total no. of genomes	i3***	i3*	i4	i3*** only	i3 only	i4 only	i3*** and i3*	i3*** and i4	i3* and i4	i3*** and i3* and i4
*Xanthomonas campestris*	125	23 (6)	44 (71)	0 (0)	6	27	0	17	0	0	0
*Xanthomonas cassavae*	2	0 (2)	2 (0)	0 (0)	0	2	0	0	0	0	0
*Xanthomonas cannabis*	4	0 (0)	0 (0)	0 (0)	0	0	0	0	0	0	0
*Xanthomonas vasicola*	100	98 (2)	0 (100)	0 (0)	98	0	0	0	0	0	0
*Xanthomonas citri*	195	0 (49)	194 (0)	14 (0)	0	180	0	0	0	14	0
*Xanthomonas oryzae*	396	359 (37)	0 (396)	364 (32)	6	0	11	0	353	0	0
*Xanthomonas phaseoli*	97	52 (3)	94 (3)	2 (0)	0	40	0	52	0	2	0
*Xanthomonas floridensis*	1	0 (0)	1 (0)	0 (0)	0	1	0	0	0	0	0
*Xanthomonas nasturtii*	2	0 (0)	0 (2)	0 (0)	0	0	0	0	0	0	0
*Xanthomonas vesicatoria*	30	0 (0)	30 (0)	0 (0)	0	30	0	0	0	0	0
*Xanthomonas cissicola*	2	0 (0)	2 (0)	2 (0)	0	0	0	0	0	2	0
*Xanthomonas hortorum*	74	0 (0)	0 (0)	5 (0)	0	0	5	0	0	0	0
*Xanthomonas prunicola*	3	0 (0)	0 (3)	0 (0)	0	0	0	0	0	0	0
*Xanthomonas codiaei*	2	1 (1)	2 (0)	0 (0)	0	1	0	1	0	0	0
*Xanthomonas dyei*	4	1 (2)	2 (2)	0 (0)	0	1	0	1	0	0	0
*Xanthomonas melonis*	6	0 (4)	6 (0)	0 (0)	0	6	0	0	0	0	0
*Xanthomonas pisi*	2	0 (2)	0 (2)	0 (0)	0	0	0	0	0	0	0
*Xanthomonas populi*	1	0 (0)	0 (0)	0 (1)	0	0	0	0	0	0	0
*Xanthomonas sacchari*	5	0 (0)	0 (0)	0 (0)	0	0	0	0	0	0	0
*Xanthomonas maliensis*	2	2 (0)	2 (0)	2 (0)	0	0	0	0	0	0	2
*Xanthomonas sontii*	5	0 (0)	0 (0)	0 (0)	0	0	0	0	0	0	0
*Xanthomonas hyacinthi*	2	0 (0)	0 (0)	0 (0)	0	0	0	0	0	0	0
*Xanthomonas cucurbitae*	2	0 (2)	0 (2)	0 (0)	0	0	0	0	0	0	0
*Xanthomonas albilineans*	17	0 (0)	0 (0)	0 (0)	0	0	0	0	0	0	0
*Xanthomonas axonopodis*	83	7 (4)	72 (11)	0 (1)	6	71	0	1	0	0	0
*Xanthomonas theicola*	2	0 (0)	0 (0)	0 (0)	0	0	0	0	0	0	0
*Xanthomonas translucens*	65	44 (4)	0 (48)	42 (3)	5	0	3	0	39	0	0
*Xanthomonas perforans*	151	148 (3)	142 (9)	0 (0)	9	3	0	139	0	0	0
*Xanthomonas bromi*	2	0 (0)	0 (0)	1 (1)	0	0	1	0	0	0	0
*Xanthomonas fragariae*	63	0 (0)	0 (0)	62 (0)	0	0	62	0	0	0	0
*Xanthomonas euroxanthea*	6	0 (0)	0 (0)	0 (0)	0	0	0	0	0	0	0
*Xanthomonas hydrangea*	3	0 (0)	0 (0)	0 (0)	0	0	0	0	0	0	0
*Xanthomonas arboricola*	136	1 (0)	7 (0)	0 (0)	0	6	0	1	0	0	0
*Xanthomonas euvesicatoria*	98	95 (1)	93 (5)	0 (0)	4	2	0	91	0	0	0
*Xanthomonas sp.*	47	1 (1)	17 (0)	1 (0)	0	15	0	1	0	1	0
*Xanthomonas alfalfae*	1	0 (1)	0 (1)	0 (0)	0	0	0	0	0	0	0
*Xanthomonas massiliensis*	2	0 (0)	0 (0)	2 (0)	0	0	2	0	0	0	0
*Xanthomonas retroflexus*	1	0 (0)	0 (0)	0 (0)	0	0	0	0	0	0	0

*Numbers of strains with partial T6 clusters are shown inside the parenthesis.*

Among early-branching strains, also known as group 1 xanthomonads, *X. translucens* and few strains belonging to unclassified species contain T6 clusters. Of 65 sequenced *X. translucens* strains, 44 strains contain intact T6 i3*** cluster, and 42 strains contain intact i4 cluster, with 39 strains containing both i3*** and i4 clusters (Table 1). While *Xanthomonas hyancinthi* genomes available in RefSeq do not contain any intact T6 cluster, *X. hyacinthi* DSM 19077 contains intact T6 i3*** cluster, with core genes of this cluster showing 100% nucleotide sequence identity to those from *Luteibacter rhizovicinus* DSM 16549 strain. This strain did not get included in Table 1 as it lacks RefSeq record. *L. rhizovicinus* is a gamma proteobacterium in the order *Xanthomonadales* isolated from the rhizosphere of barley (*Hordeum vulgare*) (Johansen et al., 2005). This observation indicates the possible independent acquisitions of the T6 clusters by individual strains within the early-branching clade from species belonging to closely related genera within *Xanthomonadaceae* family.

The size of each T6 cluster was also compared in this study. Most of the species with complete i3* cluster were 49–60 kbp whereas the i3* cluster in *X. cucurbitae* CFBP 2542 was approximately 70 kbp (Supplementary Figure 1). Shorter i3*** clusters with 23–28 kbps in size were identified in the analyzed genomes, whereas determining the size of the i4 cluster was not possible because of the presence of the i4 cluster in two loci.

### Evolutionary Patterns Among Core Genes of T6 Clusters in *Xanthomonas* spp.

**FIGURE 1:**
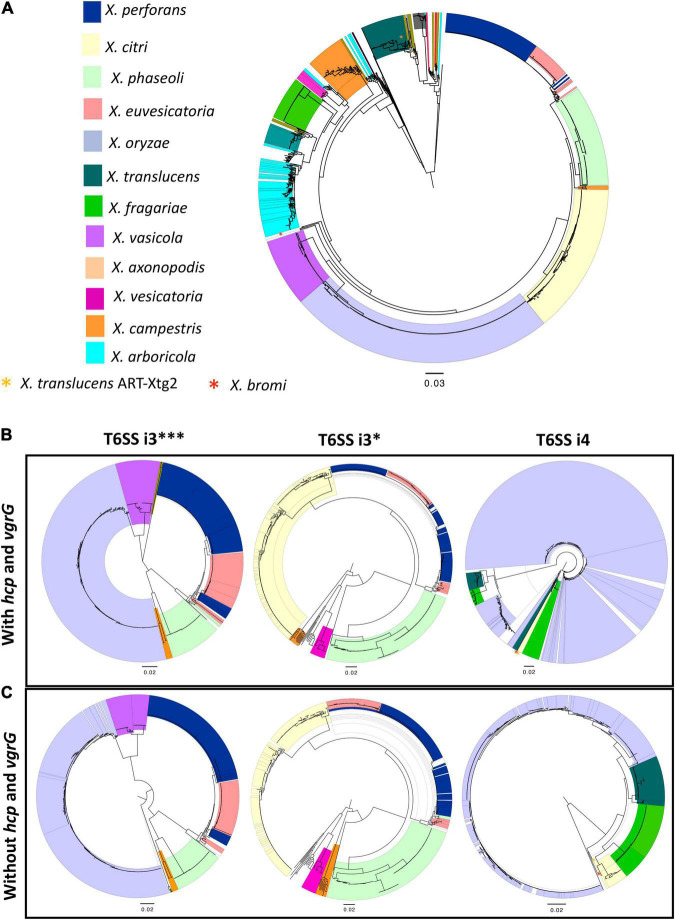
Distribution of T6 clusters in the genus *Xanthomonas.* **(A)** Relationship among 1,577 *Xanthomonas* genomes, multilocus sequence analysis (MLSA) phylogeny of *Xanthomonas* spp. based on the concatenation of partial sequences of housekeeping genes atpD, dnaK, efP, fusA, fyuA, gapA, glnA, gltA, gyrB, lacF, lepA, and rpoD. **(B)** T6 core gene phylogeny of *Xanthomonas* spp. based on the concatenation of T6 core genes *tssA*, *tssB*, *tssC*, *tssD (hcp)*, *tssE*, *tssF*, *tssG*, *tssH*, *tssI (vgrG)*, *tssJ* (only in i4 cluster), *tssK*, *tssL*, and *tssM.* **(C)** T6SS core gene phylogeny of i3***, i3*, and i4 clusters in the absence of *hcp* and *vgrG* core genes. **(B,C)** T6 core gene phylogenies were generated using the automated multilocus sequence analysis pipeline (automlsa2). Midpoint rooted phylogenetic trees in maximum likelihood (ML) criterion were reconstructed using the IQ-TREE and branch support was determined using 1,000 bootstraps and 1,000 SH-aLRT bootstrap replicates.

Phylogeny based on concatenated sequences of 12 housekeeping genes (MLSA) was compared with that of T6 i3***, i3*, and i4 core gene phylogenies including conserved effector genes, *vgrG* and *hcp* (Figure 1). Incongruencies among MLSA phylogeny and that of T6 i3* were noted for the phylogenetic placements of clades belonging to species *X. perforans* and *X. euvesicatoria*. *X. euvesicatoria* is nestled between two *X. perforans* clades, whereas *X. euvesicatoria*–related pathovars (those pathogenic on other hosts such as alfalfa, and citrus) form a separate clade based on the MLSA phylogeny. T6 i3* phylogeny with *hcp* and *vgrG* sequences reshuffled some *X. perforans* strains among the two clades. Incongruencies between MLSA phylogeny and T6 i4 cluster phylogeny with *hcp* and *vgrG* sequences were observed in case of *X. oryzae*, *X. translucens*, and *X. fragariae* species. Each of these species forms a distinct clade in the MLSA phylogeny. However, based on T6 i4 phylogeny, *X. oryzae* was highly diverse, with strains dispersed throughout the phylogeny. Some *X. translucens* and *X. fragarie* strains carry T6 i4 loci that are closely related to *X. oryzae*. *X. translucens* strain ART-Xtg2 clustered with *X. bromi* based on T6 i4 phylogeny (Figures 1A,B). No apparent incongruencies were observed when i3*** phylogeny was compared with that of the MLSA phylogeny.

Hcp and VgrG are the components of T6SS spike complex and play a role in secretion of effectors by covalently linking to the effectors. To investigate if *hcp* and *vgrG* contribute to the incongruencies observed among the above phylogeny comparisons, these two gene sequences were removed from the concatenated T6 core gene query file, and the phylogenies of T6SS core genes without *hcp* and *vgrG* sequences were reconstructed for the T6 clusters (Figure 1C). Phylogeny of core T6SS i3*** genes without *hcp* and *vgrG* showed *X. euvesicatoria*–related pathovars that form a separate clade in MLSA phylogeny, now clustering together with pepper pathogenic *X. euvesicatoria* strains. Likewise, some *X. perforans* strains that formed a distinct clade nested between *X. euvesicatoria* and *X. euvesicatoria*–related pathovars in MLSA phylogeny from the other *X. perforans* clade were now clustering together in phylogeny based on T6SS i3*** genes without *hcp* and *vgrG*. A distantly related species, *X. maliensis*, was observed closely related to *X. euvesicatoria* species complex based on T6 i3*** phylogeny with or without *hcp* and *vgrG* genes. Interestingly, comparison of i3* phylogeny without *hcp* and *vgrG* to that with *hcp* and *vgrG* showed change in the placement of some *X. perforans* strains in separate clades. The incongruencies observed with placement of *X. oryzae*, and *X. fragarie* observed in T6 i4 phylogeny with *vgrG* genes in comparison to MLSA phylogeny were resolved in the phylogeny constructed without *vgrG* genes (Figure 1). *X. oryzae* was observed as highly diverse species in phylogeny with *vgrG* and formed one distinct clade in T6 i4 phylogeny without *vgrG*, indicating a high degree of variation in *vgrG* genes in this species in addition to a gene flow with other species such as *X. translucens* and *X. fragarie*. Multiple copies of *vgrG* are presently dispersed throughout the genome of *X. oryzae* (Supplementary Figure 2). Group 1 species, *X. translucens*, was found to cluster with group 2 species, *X. oryzae* based on core gene T6 i4 phylogeny without *vgrG* genes, indicating a shared evolutionary path for T6 i4 cluster in these two species. A strain of *X. translucens*, ART-Xtg2, was an exception, which is more closely related to *X. bromi*, a group 2 species, based on T6 i4 core gene phylogeny with or without *vgrG* genes, suggesting shared evolutionary patterns for T6 i4 cluster (Figure 1).

**FIGURE 2:**
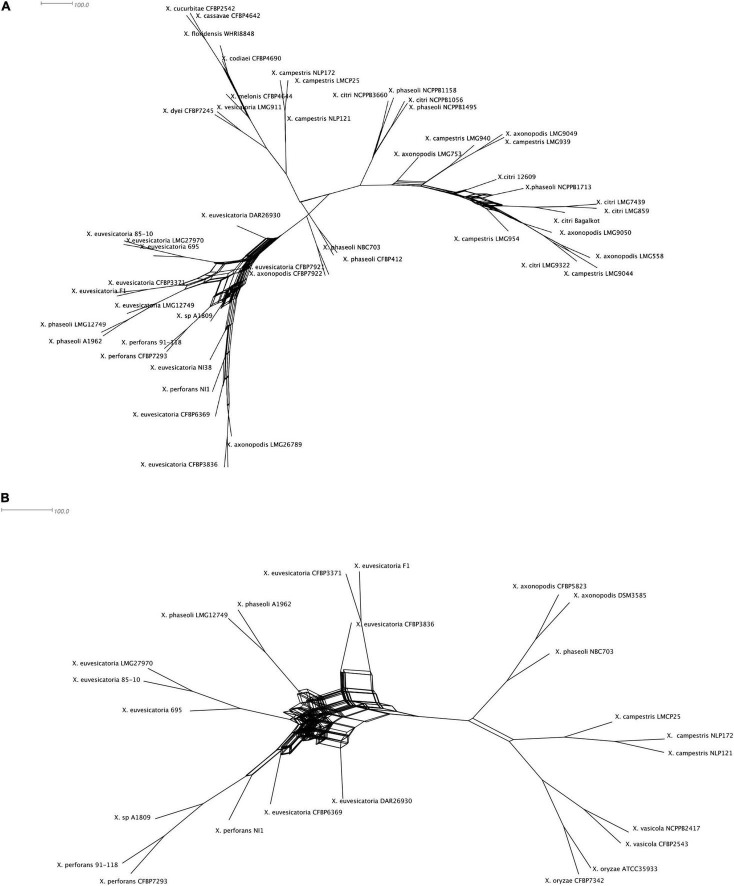
Split decomposition tree of the selected *Xanthomonas* spp. demonstrating incongruencies between the MLSA-based phylogeny and **(A)** T6 i3* without *hcp* and *vgrG* and **(B)** T6 i3*** without *hcp* and *vgrG*. The figure was drawn to scale using Splitstree4. The formation of parallel lines indicates conflicting phylogeny or possible recombination events.

Given our observation of incongruencies among MLSA phylogeny and T6SS core gene phylogeny without *hcp, vgrG* in case of clusters i3* and i3***, we hypothesized that there is genetic exchange of T6 core genes among different strains belonging to different species. We constructed phylogenetic networks using SplitsTree focusing on representatives from individual species. SplitsTree can demonstrate conflicting signals for phylogenetic placements as reticulation. Upon examining the split decomposition tree generated for T6 i3* without *hcp* and *vgrG*, we observed reticulation at the base of the branches connecting unusual recombinant strains thought to be hybrid between *X. perforans* and *X. euvesicatoria* (NI1, NI38), strains belonging to *X. euvesicatoria* sister species (CFBP 6369, F1, LMG12749, LMG 495, CFBP 3836, CFBP3371, DAR26930), commensal *Xanthomonas* strains (CFBP7921, CFBP7922), some strains belonging to *X. phaseoli* (A1962, LMG12749), *X. axonopodis* (LMG26789), and *X. euvesicatoria* 85-10 and *X. perforans* 91-118 (Figure 2A). Another evident reticulated event was identified to involve *X. citri* (LMG859, *Bagalkot*, LMG7439, 12609, NCPPB3660, NCPPB1495, NCPPB1056), *X. axonopodis* (LMG753, LMG9049, LMG9050, LMG558), and *X. campestris* strains (LMG954, LMG940, LMG939, LMG9044), indicating flow of genetic information between them with respect to T6 i3* core genes. In a split decomposition tree inferred for T6 i3*** without *hcp* and *vgrG*, a highly reticulated network encompassing above identified *X. perforans*, *X. euvesicatoria*, *X. euvesicatoria*–related pathovars, and some strains of *X. phaseoli* was observed (Figure 2B).

### Estimating the Acquisition or Loss of T6SS Clusters in the Genus *Xanthomonas* Phylogeny

**FIGURE 3:**
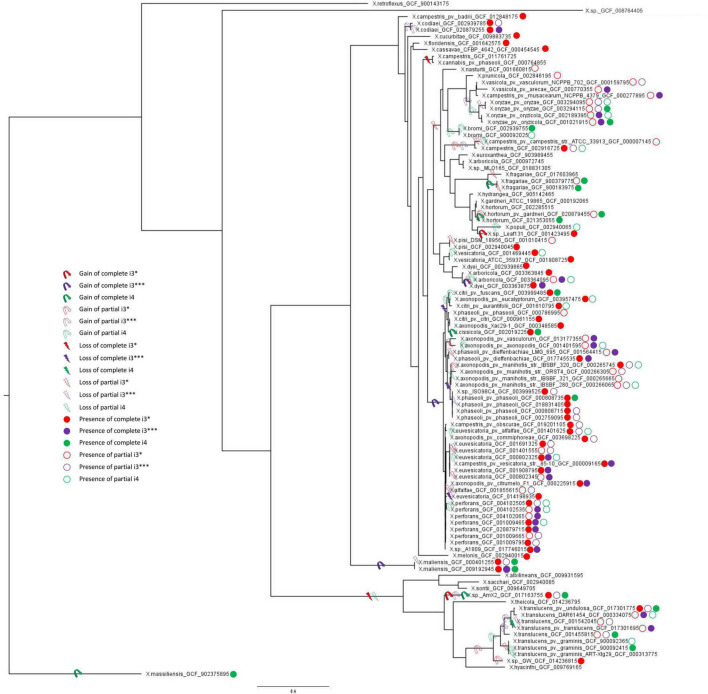
Multilocus sequence analysis phylogenetic tree and T6SS clusters presence/absence and gain/loss prediction for the genus *Xanthomonas.* Gain and loss of individual T6SS genes were mapped onto the MLSA phylogeny of 1,740 *Xanthomonas* spp. From each species, one genome with each phyletic pattern was included; that is, if two or more genomes from the same species had identical presence/absence profile of T6SS genes, only a single representative was included in the analysis. The tree was rooted using *X. massiliensis* as outgroup. The presence of complete and partial T6 clusters (i3*, i3***, and i4) in all the strains was taken from Table 1. Ancestral gain and loss events were mapped onto the tree based on the output of GLOOME. For all the predicted events, the probability was higher than 0.5.

Gene gain and loss evolutionary dynamics among bacterial species can be inferred by contrasting the reference species phylogeny to that of individual genes (Kettler et al., 2007; Cohen and Pupko, 2011; Iranzo et al., 2019). We next analyzed the evolutionary origin and distribution of multiple T6SS clusters using such an approach, as implemented in GLOOME (Cohen et al., 2010). For the reference species tree, we used a phylogenetic tree generated based on a concatenated MSA of 12 housekeeping gene sequences: *atpD*, *dnaK*, *efP*, *fusA*, *fyuA*, *gapA*, *glnA*, *gltA*, *gyrB*, *lacF*, *lepA*, and *rpoD*. A profile of presence and absence of core genes of individual T6 clusters was used as input, and the output is a probabilistic mapping of events over the reference tree (Figure 3). We additionally added clade information based on core genome phylogeny presented by Merda et al. (2017) (Supplementary Figure 4). Our results clearly present a pattern of multiple gain and loss events for all clusters (Figure 3 and Supplementary Figure 4).

The i3* cluster is present in the majority of group 2 xanthomonads, and in few *X. translucens* strains belonging to group 1. GLOOME predicted a strong signal for multiple loss events of the i3* cluster in both groups 1 and 2 *Xanthomonas* spp. For example, in group 1, the bacteria in which this cluster is present are nested among multiple strains in which the cluster is absent. Thus, a likely scenario, is that the T6 i3* cluster was acquired by lateral gene transfer event by the ancestor of the *X. translucens* clade, after its divergence from the clade that includes *X. albilineans*, *Xanthomonas sontii*, and *X. sacchari* (Figure 3). The i3* cluster is present in many but not all group 2 xanthomonads. The phyletic pattern analysis suggests that it experienced multiple independent loss events, for example, in the lineage leading to the clade that includes *X. cannabis* and also in the lineage leading to a clade that includes *X. arboricola* 3307 (Supplementary Figure 4). The presence of the i3* cluster in *X*. sp. leaf 131, a commensal *Xanthomonas*, suggests a recent independent acquisition of the cluster in that strain (Figure 3). *X. oryzae*, *X. vasicola*, *X. prunicola*, *X. bromi*, and *Xanthomonas nasturtii* have recently lost T6 i3*, whereas some strains belonging to these species have retained a partial cluster. These species belong to clade B based on Merda et al., 2017 core genome phylogeny (Supplementary Figure 4). In addition, several strains of *X. phaseoli*, *X. axonopodis*, and *X. euvesicatoria* have experienced recent partial loss of this cluster (Figure 3).

Our analysis suggests that the T6 i4 cluster was independently acquired in *X. oryzae*, *X. translucens*, *X. fragarie*, and in some strains of *X. hortorum*, *X. citri*, *X. phaseoli*, and *X. maliensis*. These multiple gain events are also supported by a SplitsTree analysis that indicated a gene flow of core genes of T6 i4 cluster among *X. oryzae* and *X. translucens*, as well as among *X. citri* and *X. hortorum*, suggesting more recent spread of this cluster with repeated events of horizontal dissemination into distinct clades across *Xanthomonas* phylogeny (Figure 2).

T6 i3*** cluster was likely gained by the ancestor of *X. axonopodis* complex, containing species *euvesicatoria*, *citri*, *phaseoli*, and *perforans*, and was subsequently lost by some species within this clade, as indicated by the presence of partial clusters in strains of these species. An independent gain is inferred to have occurred in the lineage leading to species *X. campestris* (clade D, as shown in Supplementary Figure 4), a conclusion that is corroborated by the SplitsTree analysis that indicated a gene flow of core genes of this cluster among *X. axonopodis* species complex (clade B) and *X. campestris*. The presence of the i3*** cluster in *X. maliensis* sharing higher similarity to that of *X. euvesicatoria* compared with *X. campestris* might suggest a recent independent acquisition of this cluster into *X. maliensis*. Another independent acquisition of this cluster was also identified in *X. oryzae* pv. oryzicola strains. Presence of intact T6 i3*** cluster in some *X. translucens* strains and partial in some *X. translucens* strains might indicate its presence in the ancestor of group 1. It is likely that the availability of more genomes of species belonging to group 1 might be able to resolve the phylogenetic inference of evolutionary patterns of the three clusters. Nevertheless, this analysis highlights that all three types of clusters experienced numerous transfer and loss events along the evolution of the analyzed *Xanthomonas* genomes.

### Contribution of the T6SS Toward the Evolutionary History of the *Xanthomonas axonopodis* Complex

Presence of more than one T6SS cluster was detected in *X. axonopodis* complex (i3* and i3***) and in *X. oryzae* (i3*** and i4). Strong evidence of acquisition of both i3* and i3*** by the ancestors of *X. axonopodis* species complex was obtained in aforementioned GLOOME analysis. We also observed genetic exchange of core T6 genes among different species of *X. axonopodis* in the SplitsTree analysis. Evolutionary history of *X. axonopodis* complex has been recently well studied, where it has been proposed that the first step of generalist diversification into the *X. axonopodis* subgroups was followed by the second step of ecology-driven specialization that favored the emergence of novel pathovars (Mhedbi-Hajri et al., 2013). Given our prior work that suggested the potential role of core gene *tssM* of cluster T6 i3*, in overall epiphytic fitness in *X. perforans* and during initial stages of disease development (Liyanapathiranage et al., 2021), we hypothesized that during diversification into *X. axonopodis* subgroups, a combination of specific T6SS clusters in individual species provided significant advantage during the process of niche adaptation onto specific hosts. We closely examined the patterns of the evolutionary history of the i3* and i3*** T6 clusters in the *X. axonopodis* complex subspecies groups and the lifestyle of these pathogens. When the distribution of the T6 clusters was examined according to the subspecies group they belong to, it was observed that strains belonging to the same subgroup have a similar distribution of the T6 clusters except for *X. phaseoli* pv. dieffenbachiae LMG 695 in subgroup 9.4. Strains belonging to subgroup 9.2 and *X. phaseoli* pv. dieffenbachiae LMG 695 in subgroup 9.4 possess both complete clusters, whereas strains belonging to subgroups 9.4, 9.5, and 9.6 have complete i3* clusters. Subgroup 9.3 contains only a single complete i3*** cluster, along with an incomplete i3* cluster with one to three core genes (Table 2). Interestingly, 9.3 is the only subgroup that has strains pathogenic only on monocot plants. *X. axonopodis* pv. axonopodis belonging to subgroup 9.3 are pathogenic on *Axonopus scoparius*, whereas *X. axonopodis* pv. vasculorum are pathogenic on *Saccharum officinarum.* All the other subgroups consist of strains that are pathogenic on dicot plants, except for *X. axonopodis* pv. allii (9.2) that is pathogenic on *Allium* sp.

**TABLE 2:** Distribution of the i3* and i3*** clusters in the *X. axonopodis* complex.

Subgroups	*X. axonopodis* pathovars/strains	i3***	i3*
9.1	*X. axonopodis* pv. begoniae strain CFBP2524		
	*X. axonopodis* pv. spondiae strain CFBP2623		
9.4	*X. phaseoli* pv. dieffenbachiae LMG 695	x	x
	*X. axonopodis* pv. manihotis str. CFBP1851		x
	*X. phaseoli* pv. phaseoli CFBP6546 GL1		x
9.2	*X. axonopodis* pv. citrumelo F1	x	x
	*X. axonopodis* pv. allii CFBP6369	x	x
	*X. alfalfae* subsp. alfalfae CFBP3836	x	x
	*X. euvesicatoria* 85-10	x	x
9.3	*X. axonopodis* pv. axonopodis LMG 982	x	only *tssA*
	*X. axonopodis* pv. vasculorum NCPPB900	x	only *tssA, tssB* and *tssC*
9.5	*X. citri* CFBP3369		x
	*X. citri* pv. glycines CFBP2526		x
	*X. citri* pv. malvacearum XcmN1003		x
	*X. citri* pv. mangiferaeindicae CFBP1716		x
9.6	*X. citri* pv. anacardii CFBP2913	Only *tssB*, *tssC* partial genes	x
	*X. citri* pv. phaseoli var. fuscans CFBP6165	Only *tssB*, *tssC* partial genes	x
	*X. citri* pv. fuscans CFBP6991 *phaseoli* GL2	Only *tssB*, *tssC* partial genes	x
	*X. citri* pv. fuscans pv. phaseoli GL3 CFBP6996	Only *tssB*, *tssC* partial genes	x
	*X. citri* pv. vignicola CFBP7112	Only *tssB*, *tssC* partial genes	x
	*X. citri* pv. aurantifolii ICPB 10535	Only *tssB*, *tssC* partial genes	x

*“x” indicates the presence of all the core genes.*

**FIGURE 4:**
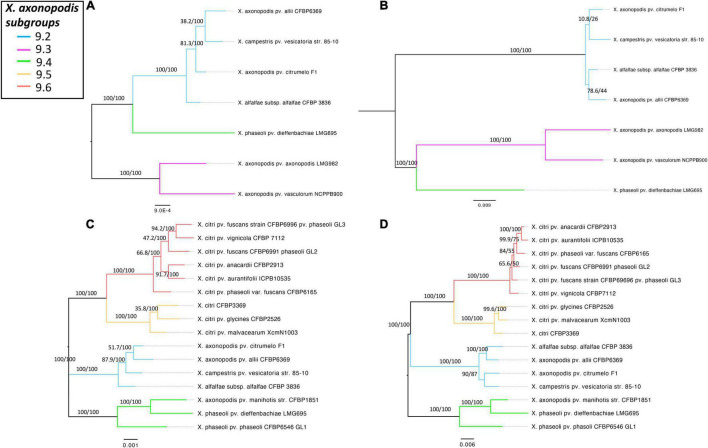
Comparison of the core genome phylogeny of *X. axonopodis* subspecies with T6 i3***/i3* core genome phylogeny to T6 i3*/i3*** core gene phylogeny. **(A)** Core genome phylogeny for the strains with i3***. **(B)** T6SS i3*** phylogeny. **(C)** Core genome phylogeny for the strains with i3*. **(D)** T6SS i3* phylogeny. Core genome phylogenies were developed based on the core alignment of nucleotide sequences using the Roary pipeline (Page et al., 2015), whereas T6 phylogenies were generated using the automated multilocus sequence analysis pipeline (automlsa2). Midpoint rooted phylogenetic tree in maximum likelihood (ML) criterion was developed using the IQ-TREE, and branch support was determined using 1,000 bootstraps and 1,000 SH-aLRT bootstrap replicates.

To determine if the T6 clusters follow the same evolutionary history as the species belonging to the *X. axonopodis* complex, a phylogeny developed based on the T6 core genes in i3*/i3*** clusters was compared with the phylogenies generated based on the core genome of strains with either i3* or i3*** core genes encoded in their genomes. Incongruencies were observed when comparing core genome phylogeny and phylogeny based on i3* or i3*** core genes (Figure 4). Core genome phylogeny clustered subgroups 9.2 and 9.4 in a single clade, whereas i3*** phylogeny clustered subgroups 9.4 and 9.3 in one clade, and i3* phylogeny indicates an early divergence of the subgroup 9.4. Clustering of subgroups 9.5 and 9.6 shows the same arrangement when core genome phylogeny was compared with the i3* phylogeny. These differences in the phylogeny hint at a possible evolution pattern in T6 clusters independent of its core genome evolution, thus further suggesting that T6 might have played a major role in host adaptation.

### T6SS Presence and Lifestyle of the Pathogen

We further assessed the distribution of T6 clusters according to vascular and non-vascular mode of colonization as well as preferred dicot versus monocot host. Most of the dicotyledonous species infecting *Xanthomonas* contain T6 i3*, whereas most of the monocotyledonous host plants infecting *Xanthomonas* contain T6 i3*** cluster (Table 3). Absence of i3* cluster was noted in the genomes of species infecting monocots. Furthermore, the T6 cluster distribution was analyzed depending on their lifestyles of vascular versus non-vascular mode. We observed presence of i4 cluster and its association with the ability of the majority of those strains to colonize vascular tissues. Notable examples of the presence of i4 cluster and demonstrated vascular colonization are mentioned below. Presence of i4 cluster was noted in only some strains of species, *X. citri*, more specifically, *X. citri* pv. fuscans and *X. citri* pv. glycines, of which *X. citri* pv. fuscans has been demonstrated to colonize vascular tissues (Table 3). While all *X. citri* strains including vascular colonizers contain intact i3* cluster, loss of i3* was seen in *X. oryzae* strains. Presence of intact i4 cluster was observed in 393 strains out of 396 sequenced genomes of *X. oryzae*. Vascular mode of infection has been demonstrated in pathovar oryzae, whereas pathovar oryzicola colonizes non-vascular tissues during infection. A partial i4 cluster was observed in some oryzicola strains. Another vascular pathogen, *X. fragariae*, encodes an intact i4 cluster, similar to *X. oryzae*, with absence of any other intact T6 cluster (Table 3). Other known vascular colonizers, *X. phaseoli* pv. manihotis and pv. dieffenbachiae, were found to lack complete i4 cluster, although a partial cluster was noted in some pathovar dieffenbachiae strains. Two *X. phaseoli* pv. phaseoli strains contain intact i4 clusters, strains of which are known to colonize both vascular and mesophyll tissues. *X. campestris*, *X. arboricola* pv. pruni, and *X. hortorum* pv. vitians can be considered as exception to the observation of association of intact i4 cluster with vascular mode of pathogenesis (Table 3). Only one sequenced strain of pathovar vitians contains intact i4 cluster. One strain of *X. hortorum* pv. gardneri was found to contain intact i4 cluster. While *X. hortorum* pv. gardneri is not known to colonize vascular tissues, Bernal et al. (2021) recently showed colonization of hydathodes upon inoculation of tomato plants with *X. hortorum* pv. gardneri. *X. translucens* strains show diversity in their preferences to colonize vascular and non-vascular tissues. However, our above observation of association of intact i4 cluster with vascular pathogenesis does not hold true with *X. translucens* species complex, which contains strains known to colonize vascular as well as non-vascular tissues. Some *X. translucens* strains despite being vascular lack T6 cluster (Table 3).

**TABLE 3:** Distribution of the T6 clusters according to the lifestyle of *Xanthomonas* spp.

*Xanthomonas* spp.	Vascular(V)/ non-vascular (NV)	Reference for tissue/host specificity	Host of isolation monocot (M)/dicot (D)	T6 clusters		
				i3***	i3*	i4
*X. floridensis*	NV	Vicente et al., 2017	D		x	
*X. nasturtii*	V	Vicente et al., 2017	D		x(partial)	
*X. vesicatoria*	NV	Potnis et al., 2015	D		x	
*X. citri* pv. citri	NV	Lee et al., 2009	D		x	
*X. citri* pv. malvacearum	NV, V	Mijatovic et al., 2021	D		x	
*X. citri* pv. fuscans	V, NV	Darrasse et al., 2018	D		x	x*
*X. perforans*	NV	Potnis et al., 2015	D	x	x	
*X. euvesicatoria*	NV	Potnis et al., 2015	D	x	x	
*X. fragariae*	V	Wang et al., 2018	D			x
*X. cucurbitae*	NV	Vieira et al., 2021	D		x	
*X. phaseoli* pv. phaseoli	NV,V	Vieira et al., 2021	D		x	x*
*X. phaseoli pv.* manihotis	V	Zarate-Chaves et al., 2021	D		x	
*X. phaseoli* pv. dieffenbachiae LMG695	V	Constantin et al., 2017	D	x*	x	
*X. cassavae*	NV	Zarate-Chaves et al., 2021	D		x	
*X. codiaei*	V	Rockey et al., 2015	D	x*	x	
*X. hortorum* pv. vitians	V	Barak et al., 2002	D			x**
*X. hortorum* pv. gardneri	NV	Bernal et al., 2021	D			x**
*X. campestris* pv. campestris	V	Bogdanove et al., 2011	D			
*X. campestris* pv. raphani	NV	Bogdanove et al., 2011	D			
*X. arboricola* pv. pruni	V	du Plessis, 1984	D			
*X. oryzae*	V	Bogdanove et al., 2011	M	x		x
*X. oryzae* pv. oryzicola	NV	Bogdanove et al., 2011	M	x		x^#^
*X. vasicola* pv. vasculorum	V	Vieira et al., 2021	M	x		
*X. campestris pv.* musacearum	V	Vieira et al., 2021	M	x		
*X. translucens* pv. translucens DSM18974	V	Gluck-Thaler et al., 2020	M	x		
*X. translucens* pv. arrhenatheri UPB455	V	Gluck-Thaler et al., 2020	M			
*X. translucens* pv. graminis ART-Xtg29	V	Gluck-Thaler et al., 2020	M			
*X. translucens* pv. graminis CNC2-P4	V	Gluck-Thaler et al., 2020	M			
*X. translucens* pv. translucens UPB787	V	Gluck-Thaler et al., 2020	M	x		x
*X. translucens* pv. cerealis NCPPB1943	V	Gluck-Thaler et al., 2020	M	x		x
*X. translucens* pv. translucens BLSB3	NV	Gluck-Thaler et al., 2020	M	x		x
*X. translucens* pv. undulosa NARK-1	NV	Gluck-Thaler et al., 2020	M	x		x
*X. translucens* DAR61454	NV	Gluck-Thaler et al., 2020	M	x		
*X. sacchari*	NV	Gluck-Thaler et al., 2020	M			

*“x” indicates the presence of all the core genes.*

“*” indicates that not all strains carry this cluster.

*“#” indicates that some strains carry partial cluster.*

“**” only one sequenced strain so far reported to carry this cluster.

### T6SS in Non-pathogenic and Environmental Strains

Next, we expanded our analysis of distribution of T6SS clusters to non-pathogenic or environmental isolates. This could provide added clarification to its role in *Xanthomonas* strains regardless of their pathogenicity. Thirteen strains collected from rainwater and 16 non-pathogenic *Xanthomonas* collected from tomato, beans, citrus, pepper, and rice seed or phyllosphere were included in a core genome phylogenetic analysis (Supplementary Table 2). Among the non-pathogenic *Xanthomonas* species, *X. maliensis* strains, early-branching strain *X. translucens* F5, and *X. floridensis* WHRI 8848 contain i3* cluster encoded in their genome. Interestingly, *X. maliensis* contains all three clusters, i3*, i3***, and i4 clusters, whereas other non-pathogenic rice leaf–associated *Xanthomonas* species such as *X. sontii* and *X. sacchari* lack any T6 clusters. Similarly, non-pathogenic *X. floridensis* WHRI 8848 isolated from watercress has a complete i3* cluster, whereas watercress pathogenic *X. nasturtii* WHRI 8853 only has a single i3* core gene with flanking regions similar to the *X. floridensis*, suggesting recent loss (Supplementary Figure 1). A commensal *Xanthomonas* strain F5 isolated from pepper belonging to group 1 contains complete i3* cluster, with core genes showing 95% to 100% identity to the genes from non-pathogenic *Xanthomonas* strains SI, SS, and GW isolated from perennial ryegrass seed (Supplementary Table 3). These strains showed antagonistic activity against key fungal pathogens. Li et al. (2020) hypothesized the involvement of unique secondary metabolites in this antagonistic activity. Our results suggest that T6SS may also play a role in this antagonism to some extent.

Among the *Xanthomonas* strains collected from the rainwater, two *X. arboricola* strains (4461 and 3793) possess a T6 i3* cluster (Supplementary Figure 4). Other *X. arboricola* strains including both known pathogens and commensals contain a partial *tssB* gene along with potential regulatory genes encoding serine/threonine protein kinase and RNA polymerase sigma factor and conserved synteny for i3* cluster, indicating recent loss.

## Discussion

In this study, we conducted a comprehensive analysis to study the distribution and the evolutionary patterns of multiple T6 clusters in the 1,740 genomes spanning the entire genus *Xanthomonas.* Complete T6 clusters, partial clusters, and even single T6 genes belonging to i3*, i3***, and i4 subgroups of T6 secretion system are dispersed throughout the genus. Our data indicate that T6SS cluster genes have been inherited both vertically and horizontally throughout the genus phylogeny. Like other genera, *Xanthomonas* species contain multiple copies of T6 clusters, with presence of either i3* + i3*** or i3* + i4 combinations being frequently observed. *X. maliensis*, associated with healthy rice seeds or leaves in Mali (Triplett et al., 2015), was the only species identified carrying all three clusters, i3*, i3***, and i4. Being an early branching species within group 2 xanthomonads, conservation of all three clusters in this species and the genomic context is important to infer evolutionary scenarios explaining gain/loss events of the three types of T6 clusters. Simultaneous maintenance of all three clusters in the species is surprising, given the energy investment in these large clusters. Sequencing multiple strains of this species might help address whether presence of all three clusters may represent random flicker or a sign of positive selection for acquisition in the evolutionary history. If the latter is true, this species would represent an ideal candidate for studying differential regulation of each cluster and how the environment influences this regulation.

The majority of the species belonging to *X. axonopodis* complex possess both i3* and i3*** clusters, with exception of *X. citri* that lacks cluster i3***. The loss of i3*, observed in clade A strains and strains of *X. oryzae*, and *X. vasicola* from clade B, was accompanied by gain of i4 cluster. Some strains belonging to *X. citri* and *X. phaseoli*, which possess both i3 and i4 clusters in 1% to 5% of the strains are an exception to the rule of two i3 clusters in *X. axonopodis* complex and to the observation of replacement of i3 by i4. The identification of two *X. phaseoli* strains with the i4 cluster was an unusual observation as the *X. phaseoli* strains were previously only known to harbor i3* and i3*** clusters (Montenegro Benavides et al., 2021). This highlights the importance of the availability of genomes of multiple hundreds of strains of each species for understanding intraspecific diversity. It is unclear whether the loss of i3* and subsequent gain of i4 cluster in species such as *X. oryzae*, and *X. vasicola*, was in response to selection pressure from the environment including resident microflora.

The role of i3* has been suggested not only in competition against resident flora, more specifically, resistance to predation by the bacterivorous amoeba, *Dictyostelium discoideum*, but also in adaptation to the phyllosphere non-vascular environment (Bayer-Santos et al., 2018; Liyanapathiranage et al., 2021). Loss of i3* in lineages containing *X. oryzae* and *X. vasicola*, both vascular pathogens, might be in response to the vascular lifestyle. In the rice pathogen *X. oryzae* pv. oryzicola, T6SS i4 has been identified for its role in the interbacterial competition, whereas in *X. oryzae* pv. oryzae, it was identified for its role in virulence (Choi et al., 2020; Zhu et al., 2020; Montenegro Benavides et al., 2021).

As it has been identified that presence of all the core T6SS genes is required for the functionality of the T6SS (Boyer et al., 2009), it is intriguing to identify some strains that lack single T6 genes and raise the questions about the possible function of that apparently inactive T6 cluster. In our analysis, we observed the T6SS locus lacking *clpV* gene in some of the *X. oryzae* strains, and interestingly, in previous studies on the distribution of the T6 clusters in *Campylobacter jejuni*, T6SS locus lacking the *clpV (tssH)* gene has been noted (Bönemann et al., 2009; Bleumink-Pluym et al., 2013). Even though ClpV is required for the recycling of the TssB/TssC tubular sheath, in *Vibrio cholerae*, a *clpV* mutant was still effective in killing *Escherichia coli*, thus indicating the possibility of function of a *clpV*-related ClpB family of ATPase encoded from elsewhere compensating for the lack of *clpV* in the T6SS (Pietrosiuk et al., 2011; Zheng et al., 2011).

The distribution of T6SS among *Xanthomonas* species does not correlate with the host preference. Four *Xanthomonas* spp. that cause bacterial leaf spot in tomato and pepper show the diverse distribution of T6 clusters. *X. euvesicatoria* and *X. perforans* have both i3* and i3*** clusters; whereas *X. vesicatoria* has i3*cluster, *X. hortorum pv.* gardneri does not have T6 clusters encoded in its genome, with the exception of a single strain carrying i4 cluster (Potnis et al., 2011). Even the three species that have the i3* cluster show differences in cluster organization (Supplementary Figure 3), with different set of putative effectors. Another example could be of rice-associated strains. An extensive diversity in the T6 cluster distribution was observed in the rice-associated xanthomonads, ranging from absence of T6 clusters in non-pathogenic strains, *X. sontii* (Bansal et al., 2019) and *X. sacchari* R1 (Fang et al., 2015), to presence of i4 alone or i3*** and i4 clusters in pathogenic *X. oryzae* and *X. oryzicola*. Another such example is of watercress-associated strains. A non-pathogenic strain, *X. floridensis* WHRI 8848 isolated from the diseased leaves of watercress (*Nasturtium officinale*) (Vicente et al., 2017), contains intact i3* cluster, but a pathogenic strain, *X. nasturtii* WHRI 8853, lacks a functional cluster and encodes only a single partial T6SS gene (*tssA*), indicating a recent loss of the cluster with similar flanking regions.

The distribution of T6 clusters does not depend on host of isolation or pathogenic status. We observed presence of intact T6 clusters in non-pathogenic or commensal strains, even those recovered from rainwater. This observation highlights the role of T6 clusters and associated effectors in conferring fitness during host colonization and also possibly outside the host environment, in conferring traits such as nutrient acquisition, or bacterial communication. While host environment has been suggested to contribute to induction of T6 clusters in pathogenic bacteria, commensal strains would be an ideal background to understand how T6SS is regulated in absence of network of other virulence factors, although constitutive expression of T6SS cannot be ruled out as observed in *V. cholerae* non-pathogenic strains (Lopez et al., 2020).

Based on the probability of gain/loss events during *Xanthomonas* evolution, as well as information gathered by analysis of flanking regions of T6 clusters and identities of core genes, the data obtained in this study indicate that T6SS was already present in the ancestors of the major xanthomonad lineages. When we mapped the presence of various T6 clusters onto the MLSA tree, we inferred numerous gain events via horizontal gene transfer as well as many loss events. We hypothesize that such evolutionary dynamics is driven by selective pressure related to adaptation to specific environments. In case of T6 i3* cluster, a conserved flanking region across group 2 xanthomonads indicate vertical acquisition of T6 clusters by a common ancestor in a single acquisition event. However, T6 i3* cluster experienced subsequent loss in several lineages, including those in *X. arboricola*, *X. oryzae*, and *X. hortorum*, and has left signatures within genomes, such as partial cluster, or presence of tRNA indicating a rearrangement event. This loss was also followed by gain of T6 i4 cluster in some vascular pathogenic species such as *X. oryzae* and *X. fragarie*. T6 i4 gain events were identified as independent events across the phylogeny. Recent gain of the i4 cluster via horizontal transfer events in multiple species was further confirmed by the fact that flanking regions of the i4 cluster show extreme heterogeneity.

Comparing T6 cluster core gene phylogenies to that of housekeeping genes suggested gene flow of core genes among different species of xanthomonads. This is in agreement with previous findings of T6SS gene clusters as recombination hotspots in *X. euvesicatoria* species complex (Newberry et al., 2019), which suggests reshuffling of alleles of core genes of T6 clusters. In addition to core genes, we also observed reticulations in phylogenetic network, indicating frequent exchange of Hcp and VgrG among *Xanthomonas* species. Such events of reshuffling of VgrG and Hcp have been observed as a strong force in diversification of effector-immunity proteins in many genera, such as *Agrobacterium* and *Vibrio* (Salomon et al., 2015; Thomas et al., 2017; Wu et al., 2021). Although it is true that poorly resolved phylogenies or phylogenies of closely related strains with low branch support could appear as reticulations in the network, reticulations observed specifically for distantly related species suggest that recombination could be a highly likely explanation in those cases.

Species belonging to the *X. axonopodis* complex are pathogenic on a wide range of crops (Vauterin et al., 1995) and are classified into six groups named 9.1 to 9.6 within *X. axonopodis* (Rademaker et al., 2005; Young et al., 2008; Hajri et al., 2009; Bui Thi Ngoc et al., 2010; Mhedbi-Hajri et al., 2013). According to the core genome phylogeny and i3* or i3*** core genes phylogeny in this current study, we observe the same pattern in the way each strain categorizes into the subgroupings as explained in the previous literature, suggesting acquisition of these clusters by the ancestor of *X. axonopodis*. Mhedbi-Hajri et al. (2013) speculated that the first step of generalist diversification was followed by a second step of ecology-driven specialization that probably occurred in the past two centuries and favored the emergence of novel pathovars. This second event of host specialization has occurred likely through agricultural intensification and expansion that facilitated genetic exchanges of virulence-associated genes. All of these pathovars are restricted to one of the six subgroups and most of them formed monophyletic (e.g., citri, mangiferaeindicae, malvacearum, begonia, and manihotis) clusters, whereas some strains were polyphyletic (e.g., dieffenbachiae, glycines, and phaseoli) (Hajri et al., 2009; Mhedbi-Hajri et al., 2013). Some strains that belong to the same pathovar can be found in phylogenetically distant clusters. For example, *X. axonopodis* strains that infect legumes can be found in subgroups 9.2, 9.4, and 9.6; mango *Mangifera indica*–infecting strains were found in subgroups 9.5 and 9.6, and citrus-infecting strains were identified in clusters 9.2, 9.5, and 9.6. Similarly, *X. axonopodis* strains that are pathogenic on phylogenetically distant hosts are clustered in the same subgroups, as seen with subgroup 9.5, where citrus-, mango-, legume-, and cotton-infecting *X. axonopodis* strains are grouped together. An independent rapid evolution in the subgroup 9.3 compared with the other subgroups might also be reflected in its loss of i3* cluster during niche adaptation. *X. phaseoli* pv. dieffenbachiae is polyphyletic and found in subgroups 9.6 (A- *Dieffenbachia* sp.), 9.2 (B- *Philodendron* sp.) and 9.4 (C- *Anthurium* sp.) according to the *rpoD* sequences analysis and Rep-PCR, this grouping is also dependent on the host of isolation (Hajri et al., 2009; Mhedbi-Hajri et al., 2013). Our observation of heterogeneity of T6 cluster distribution in the subgroup 9.4 suggests preferences in specific T6 clusters during niche adaptation of different host species. This phylogenetic analysis and T6SS cluster distributions suggest that T6SS cluster conservation and associated effector alleles might have played a role during host specialization in the *X. axonopodis* strains in addition to the already suggested factors such as methyl-accepting chemotaxis proteins and type III effectors (Hajri et al., 2009; Mhedbi-Hajri et al., 2013).

In conclusion, our findings point to the scenario in which the different T6 clusters are often gained and lost during the evolution of *Xanthomonas* species, with some species encoding two clusters, whereas others, not a single one. We hypothesize that the majority of gain and loss events are not random, but rather reflect adaptations and specialization to specific environments. The factors involved in niche adaptation have been of interest in case of plant pathogenic bacteria, and a new paradigm has suggested involvement of multiple genetic determinants underlying this process with small to major effect genes during the process of adaptation of a pathogen to a host type or to a particular tissue (Bogdanove et al., 2011; Huang et al., 2015; Jacques et al., 2016). Here, we explored association of T6 clusters and preference of *Xanthomona*s species toward dicot or monocot hosts and vascular or non-vascular mode of infection. As not all species have been studied for their breadth of host range or tissue colonization, the associations evaluated here are based on current literature. We noted the absence of i3* cluster in monocot-infecting pathogens. The gain/loss patterns also indicated loss of i3* followed by gain of i4 cluster in vascular pathogenic species in group 2 xanthomonads, such as *oryzae* and *fragarie*. But this observation was not true in case of *X. translucens* species complex and *X. campestris* pv. campestris, *X. vasicola*. In brief, there does not seem to be association of specific T6 cluster for a lifestyle of *Xanthomonas* when evaluating the entire genus as a whole. However, functional characterization using T6SS-defective mutants of *Xanthomonas* has suggested importance of T6SS in overall fitness during host colonization, as well as its role in competing with the resident flora. As pathogen fitness criteria vary depending on the niche that pathogen experiences, involvement of T6 cluster/s and its role in regulatory network may vary depending on the host environment. Thus, it is likely that associations that we have observed with gain/loss patterns for a specific T6 cluster may be valid for specific clades that experience similar host environments or that harness similar virulence factor repertoires to coordinate the process of colonization. It is also possible that secreted effectors or toxins may be more important in specialization to the host environment rather than association of a specific T6 cluster/s.

## Data Availability Statement

The original contributions presented in the study are included in the article/Supplementary Material, further inquiries can be directed to the corresponding author/s.

## Author Contributions

NP conceived the project. PL, NW, and NP conducted the comparative genome analyses and SplitsTree analysis, generated the core T6 gene and core genome phylogenies, and wrote the manuscript. OA conducted the Gloome analysis. OA, NP, NW, and TP interpreted the Gloome analysis results. NW, OA, and TP edited the manuscript. All authors approved final manuscript.

## Conflict of Interest

The authors declare that the research was conducted in the absence of any commercial or financial relationships that could be construed as a potential conflict of interest.

## Publisher’s Note

All claims expressed in this article are solely those of the authors and do not necessarily represent those of their affiliated organizations, or those of the publisher, the editors and the reviewers. Any product that may be evaluated in this article, or claim that may be made by its manufacturer, is not guaranteed or endorsed by the publisher.
