# Supplementary material for: Phylogenetic Distribution and Evolution of Type VI Secretion System in the Genus Xanthomonas
Source: Front Microbiol. 2022 Apr 14;13:840308. doi: 10.3389/fmicb.2022.840308 (PMC9048695; doi:10.3389/fmicb.2022.840308)
Supplement: Supplementary file 1 [file Data_Sheet_1.PDF]

Supplementary Material

1 Supplementary Figures and Tables

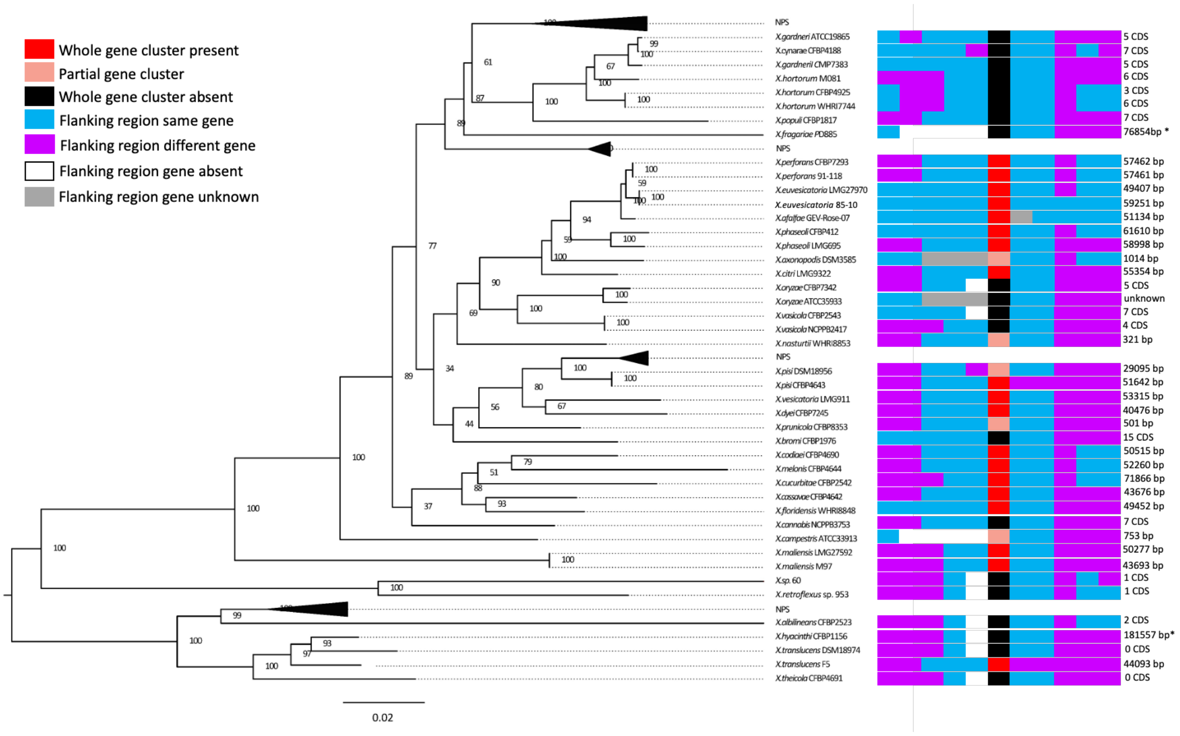

Supplementary Figure 1. Presence of T6 cluster i3* in *Xanthomonas* spp. with flanking regions. Phylogenetic distribution of 43 *Xanthomonas* spp. based on the core alignment of nucleotide sequences using the Roary pipeline and branch support was determined using 1000 bootstraps (Page et al., 2015). Whole-genome sequences of type strains or completely sequenced genomes representing the *Xanthomonas* spp. available in the National Center for Biotechnology Information (NCBI) database were used for phylogenetic reconstruction. Flanking regions of the T6 clusters were identified using the (IMG/M) (v.6.0) system and genome and microbiome datasets sequenced at DOE's Joint Genome Institute. Presence of all the core genes in the i3* cluster (Red), absence of one or more core genes in

the i3* cluster (pink) or the absence of all the core genes (black) have been indicted with different colors and can be found in the middle of up and downstream genes. Genomic environments, similar (blue) and different (purple) genes found in the up and downstream of the T6 i3* cluster are represented with colors. Comparisons were done using the *X. vesicatoria* 85-10 as the reference strain. Size of the T6 i3* cluster is mentioned if the cluster is present or the number of CDS or the length of the DND fragment (*) found in place of the i3*cluster has been included in the last column of the figure.

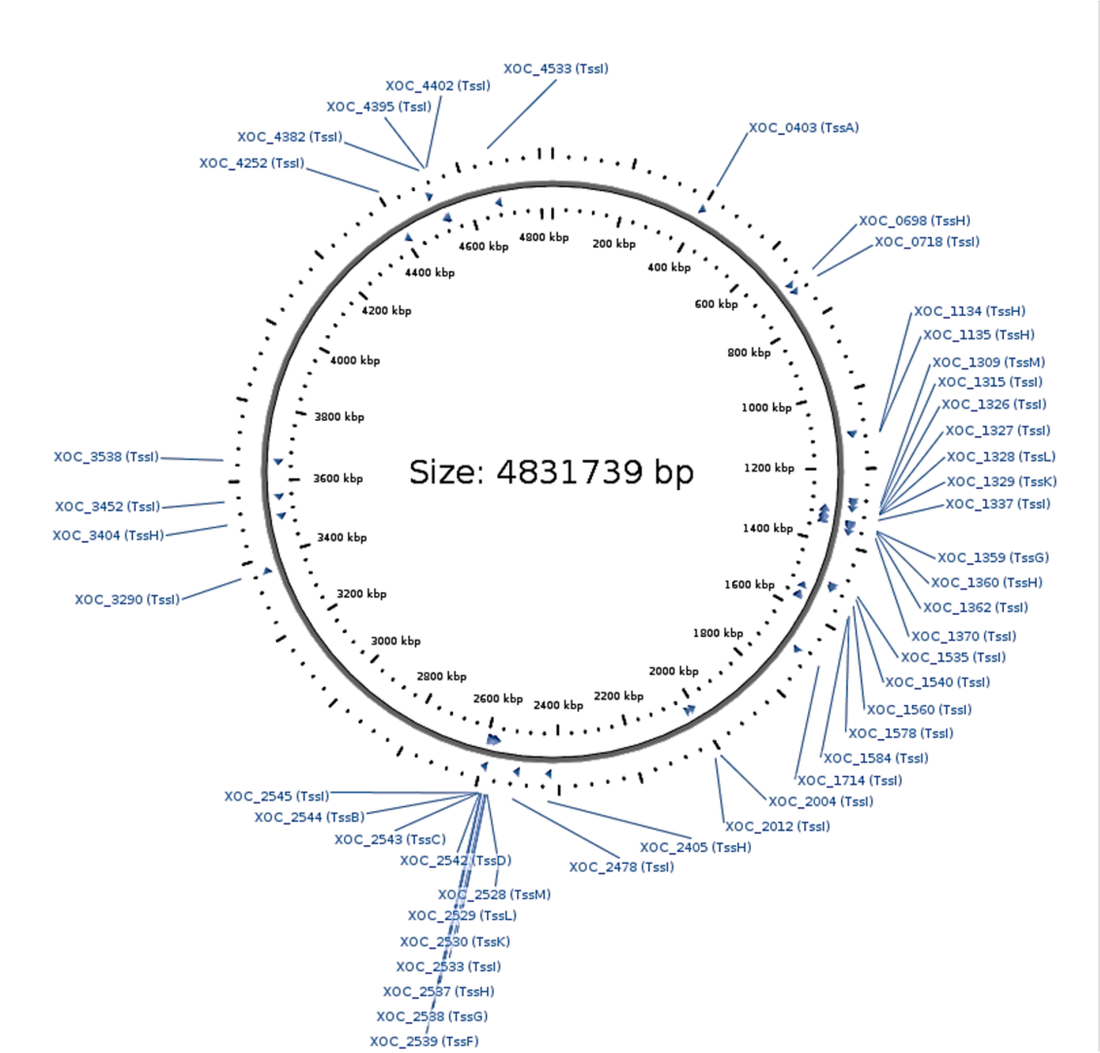

Supplementary Figure 2. Distribution of the *VgrG (tssI)* genes in the *Xanthomonas oryzae* pv. *oryzicola* BLS256 genome.

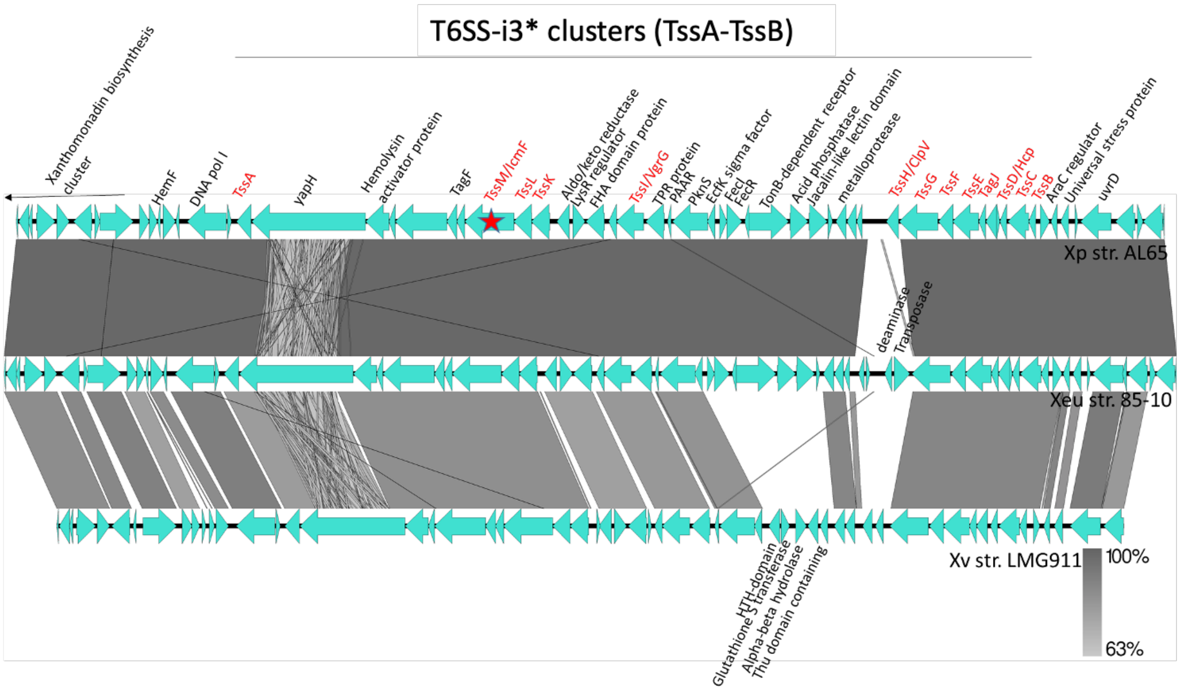

Supplementary Figure 3. Organization of the T6SS-i3* clusters in BLS *Xanthomonas.* (a) *XpAL65* (b) *Xe85-10* and (c) *XvLMG911*

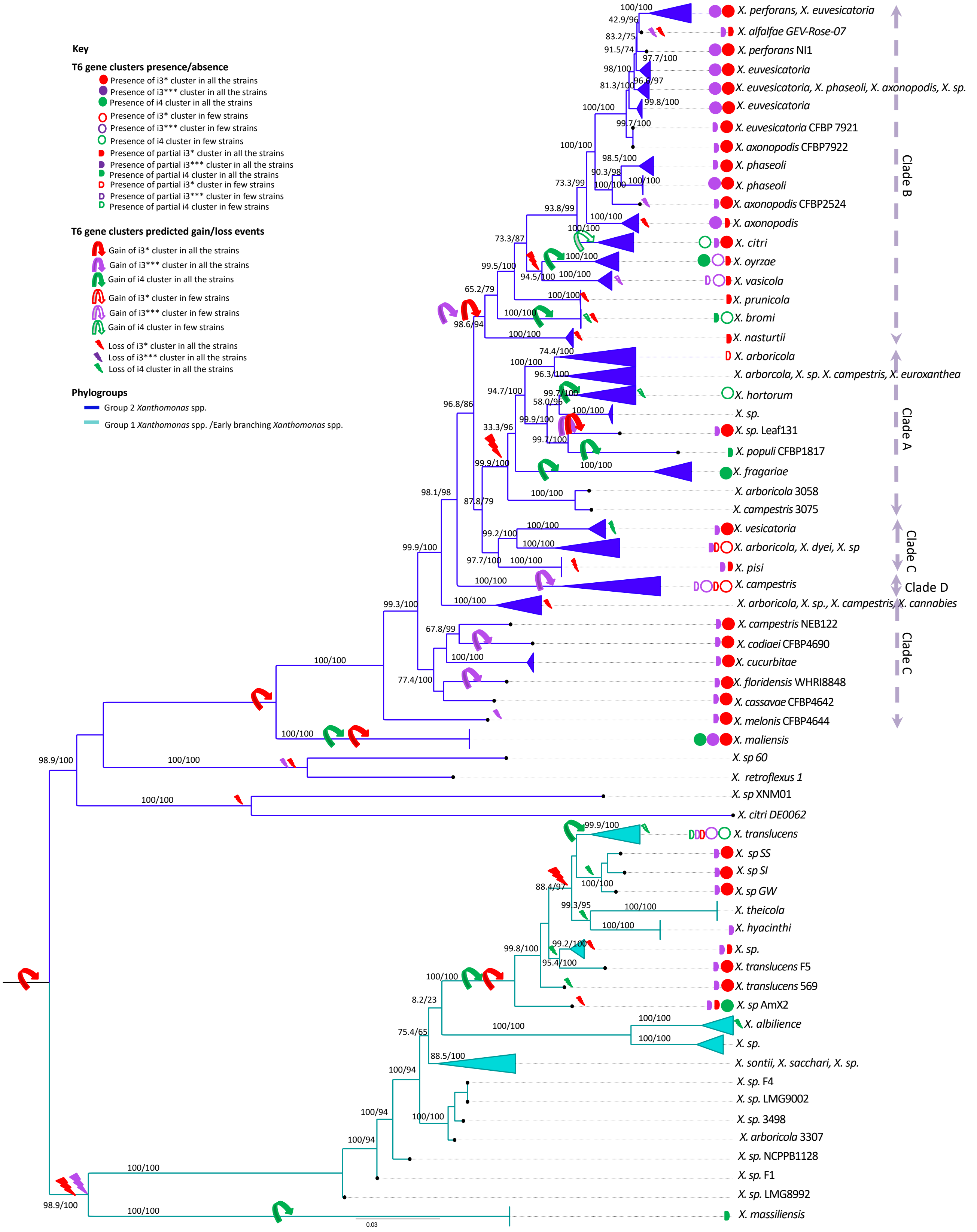
Key
T6 gene clusters presence/absence
Presence of i3* cluster in all the strains
Presence of i3*** cluster in all the strains
Presence of i4 cluster in all the strains
Presence of i3* cluster in few strains
Presence of i3*** cluster in few strains
Presence of i4 cluster in few strains
Presence of partial i3* cluster in all the strains
Presence of partial i3*** cluster in all the strains
Presence of partial i4 cluster in all the strains
Presence of partial i3* cluster in few strains
Presence of partial i3*** cluster in few strains
Presence of partial i4 cluster in few strains
T6 gene clusters predicted gain/loss events
Gain of i3* cluster in all the strains
Gain of i3*** cluster in all the strains
Gain of i4 cluster in all the strains
Gain of i3* cluster in few strains
Gain of i3*** cluster in few strains
Gain of i4 cluster in few strains
Loss of i3* cluster in all the strains
Loss of i3*** cluster in all the strains
Loss of i4 cluster in all the strains
Phylogroups
Group 2 Xanthomonas spp.
Group 1 Xanthomonas spp. /Early branching Xanthomonas spp.
X. perforans, X. euvesicatoria
X. alfalfae GEV-Rose-07
X. perforans NI1
X. euvesicatoria
X. euvesicatoria, X. phaseoli, X. axonopodis, X. sp.
X. euvesicatoria
X. euvesicatoria CFBP 7921
X. axonopodis CFBP7922
X. phaseoli
X. phaseoli
X. axonopodis CFBP2524
X. axonopodis
X. citri
X. oyrzae
X. vasicola
X. prunicola
X. bromi
X. nasturtii
X. arboricola
X. arborcola, X. sp. X. campestris, X. euroxanthea
X. hortorum
X. sp.
X. sp. Leaf131
X. populi CFBP1817
X. fragariae
X. arboricola 3058
X. campestris 3075
X. vesicatoria
X. arboricola, X. dyei, X. sp
X. pisi
X. campestris
X. arboricola, X. sp., X. campestris, X. cannabies
X. campestris NEB122
X. codiaei CFBP4690
X. cucurbitae
X. floridensis WHRI8848
X. cassavae CFBP4642
X. melonis CFBP4644
X. maliensis
X. sp 60
X. retroflexus 1
X. sp XNM01
X. citri DE0062
X. translucens
X. sp SS
X. sp SI
X. sp GW
X. theicola
X. hyacinthi
X. sp.
X. translucens F5
X. translucens 569
X. sp AmX2
X. albilience
X. sp.
X. sontii, X. sacchari, X. sp.
X. sp. F4
X. sp. LMG9002
X. sp. 3498
X. arboricola 3307
X. sp. NCPPB1128
X. sp. F1
X. sp. LMG8992
X. massiliensis
Clade B
Clade A
Clade C
Clade D
Clade C
0.03

Supplementary Figure 4. MLSA phylogenetic tree and T6SS clusters presence/absence and gain/loss prediction for the genus *Xanthomonas.* Multi-locus sequence analysis (MLSA) phylogeny of all *Xanthomonas* spp. Analyzed in this study based on the concatenation of partial sequences of housekeeping genes *atpD, dnaK, efP, fusA, fyuA, gapA, glnA, gltA, gyrB, lacF, lepA* and *rpoD.* Midpoint rooted phylogenetic tree in maximum likelihood (ML) criterion was developed using the IQ-TREE multicore version 2.1.2 COVID-edition. Branches were collapsed based on the similarities in the T6 clusters on each node. The phylogroup to which each strain belongs is indicated by the color of the branch. The presence of complete and partial T6 clusters (i3*, i3***, and i4) in all the strains or only in a few strains found in a single clade are represented by color-coded complete or half circles respectively. Probable T6SS cluster gain or loss events are represented by arrows and thunder bullets respectively. Prediction of T6SS clusters gain and loss events were performed using GLOOME.

Supplementary Table 1. T6SS cluster presence-absence table with accession numbers and *Xanthomonas* species names.

RefSeq_accession	Organism	T6-i3*	T6-i3***	T6-i4
GCF_000007145.1	Xanthomonas campestris pv. campestris str. ATCC 33913	partial	None	None
GCF_000277895.2	Xanthomonas campestris pv. musacearum NCPPB 4379	partial	full	None
GCF_000454545.1	Xanthomonas cassavae CFBP 4642	full	partial	None
GCF_020783895.1	Xanthomonas cassavae CFBP 4642	full	partial	None
GCF_000764855.1	Xanthomonas cannabis pv. phaseoli	None	None	None
GCF_000770355.2	Xanthomonas vasicola pv. arecae	partial	full	None
GCF_000961155.1	Xanthomonas citri pv. citri	full	None	None
GCF_000961195.1	Xanthomonas citri pv. citri	full	None	None
GCF_000961215.1	Xanthomonas citri pv. citri	full	None	None
GCF_000961235.1	Xanthomonas citri pv. citri	full	None	None
GCF_000961255.1	Xanthomonas citri pv. citri	full	None	None
GCF_000961275.1	Xanthomonas citri pv. citri	full	None	None
GCF_000961295.1	Xanthomonas citri pv. citri	full	None	None
GCF_000961315.1	Xanthomonas citri pv. citri	full	None	None
GCF_000961335.1	Xanthomonas citri pv. citri	full	None	None
GCF_000961355.1	Xanthomonas citri pv. citri	full	None	None
GCF_000961375.1	Xanthomonas citri pv. citri	full	None	None
GCF_000961395.1	Xanthomonas citri pv. citri	full	None	None
GCF_000961415.1	Xanthomonas citri pv. citri	full	None	None
GCF_000961435.1	Xanthomonas citri pv. citri	full	None	None
GCF_000961455.1	Xanthomonas citri pv. citri	full	None	None
GCF_000961475.1	Xanthomonas citri pv. citri	full	None	None
GCF_000961495.1	Xanthomonas citri pv. citri	full	None	None
GCF_001028285.3	Xanthomonas citri pv. citri	full	None	None

**GCF_001922045.1**	Xanthomonas citri pv. citri	full	None	None
**GCF_001922065.1**	Xanthomonas citri pv. citri	full	None	None
**GCF_001922085.1**	Xanthomonas citri pv. citri	full	None	None
**GCF_001922105.1**	Xanthomonas citri pv. citri	full	None	None
**GCF_002139955.1**	Xanthomonas citri pv. citri	full	None	None
**GCF_002139975.1**	Xanthomonas citri pv. citri	full	None	None
**GCF_002139995.1**	Xanthomonas citri pv. citri	full	None	None
**GCF_002952295.1**	Xanthomonas citri pv. citri	full	None	None
**GCF_003665455.1**	Xanthomonas citri pv. citri	full	None	None
**GCF_003665475.1**	Xanthomonas citri pv. citri	full	None	None
**GCF_016801615.1**	Xanthomonas citri pv. citri	full	None	None
**GCF_016801635.1**	Xanthomonas citri pv. citri	full	None	None
**GCF_016801655.1**	Xanthomonas citri pv. citri	full	None	None
**GCF_016801675.1**	Xanthomonas citri pv. citri	full	None	None
**GCF_016801695.1**	Xanthomonas citri pv. citri	full	None	None
**GCF_019076985.1**	Xanthomonas citri pv. citri	full	None	None
**GCF_019434315.1**	Xanthomonas citri pv. citri	full	None	None
**GCF_019434335.1**	Xanthomonas citri pv. citri	full	None	None
**GCF_001401655.1**	Xanthomonas citri pv. citri	full	partial	None
**GCF_001939965.1**	Xanthomonas citri pv. citri	full	None	None
**GCF_001939985.1**	Xanthomonas citri pv. citri	full	None	None
**GCF_002018575.1**	Xanthomonas citri pv. citri	full	None	None
**GCF_014694055.1**	Xanthomonas citri pv. citri	full	None	None
**GCF_000825185.2**	Xanthomonas citri pv. citri	full	None	None
**GCF_000825205.2**	Xanthomonas citri pv. citri	full	None	None
**GCF_000825225.2**	Xanthomonas citri pv. citri	full	None	None
**GCF_000825245.2**	Xanthomonas citri pv. citri	full	None	None
**GCF_000825265.2**	Xanthomonas citri pv. citri	full	None	None
**GCF_000825285.2**	Xanthomonas citri pv. citri	full	None	None
**GCF_000825305.2**	Xanthomonas citri pv. citri	full	None	None
**GCF_000825325.2**	Xanthomonas citri pv. citri	full	None	None
**GCF_000825345.2**	Xanthomonas citri pv. citri	full	None	None
**GCF_000825365.2**	Xanthomonas citri pv. citri	full	None	None
**GCF_000825385.2**	Xanthomonas citri pv. citri	full	None	None
**GCF_000825405.2**	Xanthomonas citri pv. citri	full	None	None
**GCF_000825425.2**	Xanthomonas citri pv. citri	full	None	None
**GCF_000825465.2**	Xanthomonas citri pv. citri	full	None	None
**GCF_000825485.2**	Xanthomonas citri pv. citri	full	None	None
**GCF_000825505.2**	Xanthomonas citri pv. citri	full	None	None
**GCF_000825525.2**	Xanthomonas citri pv. citri	full	None	None
**GCF_000825545.2**	Xanthomonas citri pv. citri	full	None	None

**GCF_000825565.2**	Xanthomonas citri pv. citri	full	None	None
**GCF_000825605.2**	Xanthomonas citri pv. citri	full	None	None
**GCF_000829945.2**	Xanthomonas citri pv. citri	full	None	None
**GCF_000950845.1**	Xanthomonas citri pv. citri	full	None	None
**GCF_000950855.1**	Xanthomonas citri pv. citri	full	None	None
**GCF_000950875.1**	Xanthomonas citri pv. citri	full	None	None
**GCF_001956275.1**	Xanthomonas citri pv. citri	full	None	None
**GCF_002742455.1**	Xanthomonas citri pv. citri	full	None	None
**GCF_003122545.1**	Xanthomonas citri pv. citri	full	None	None
**GCF_003122565.1**	Xanthomonas citri pv. citri	full	None	None
**GCF_003122605.1**	Xanthomonas citri pv. citri	full	None	None
**GCF_003122625.1**	Xanthomonas citri pv. citri	full	None	None
**GCF_014694075.1**	Xanthomonas citri pv. citri	full	None	None
**GCF_014694175.1**	Xanthomonas citri pv. citri	full	None	None
**GCF_014694185.1**	Xanthomonas citri pv. citri	full	None	None
**GCF_001021915.1**	Xanthomonas oryzae pv. oryzicola	partial	full	full
**GCF_000940825.1**	Xanthomonas oryzae pv. oryzicola	partial	full	full
**GCF_001042735.1**	Xanthomonas oryzae pv. oryzicola	partial	full	full
**GCF_001042745.1**	Xanthomonas oryzae pv. oryzicola	partial	full	full
**GCF_001042775.1**	Xanthomonas oryzae pv. oryzicola	partial	full	full
**GCF_001042795.1**	Xanthomonas oryzae pv. oryzicola	partial	full	full
**GCF_001042815.1**	Xanthomonas oryzae pv. oryzicola	partial	full	full
**GCF_001042835.1**	Xanthomonas oryzae pv. oryzicola	partial	full	full
**GCF_001042855.1**	Xanthomonas oryzae pv. oryzicola	partial	full	full
**GCF_001042875.1**	Xanthomonas oryzae pv. oryzicola	partial	full	full
**GCF_008370835.2**	Xanthomonas oryzae pv. oryzicola	partial	full	full
**GCF_009660205.1**	Xanthomonas oryzae pv. oryzicola	partial	full	full
**GCF_020084945.1**	Xanthomonas oryzae pv. oryzicola	partial	full	full
**GCF_003064145.1**	Xanthomonas oryzae pv. oryzicola	partial	full	full
**GCF_001929305.1**	Xanthomonas oryzae pv. oryzicola	partial	full	full
**GCF_002189395.1**	Xanthomonas oryzae pv. oryzicola	partial	full	partial
**GCF_002189435.1**	Xanthomonas oryzae pv. oryzicola	partial	full	partial
**GCF_002189465.1**	Xanthomonas oryzae pv. oryzicola	partial	full	full
**GCF_001564415.1**	Xanthomonas phaseoli pv. dieffenbachiae LMG 695	full	full	None
**GCF_001402235.1**	Xanthomonas phaseoli pv. dieffenbachiae LMG 695	full	full	None
**GCF_001642575.1**	Xanthomonas floridensis	full	None	None
**GCF_001660815.1**	Xanthomonas nasturtii	partial	None	None
**GCF_003409975.1**	Xanthomonas nasturtii	partial	None	None
**GCF_001719145.1**	Xanthomonas citri pv. malvacearum	full	None	None
**GCF_002224525.1**	Xanthomonas citri pv. malvacearum	full	None	None
**GCF_002224545.1**	Xanthomonas citri pv. malvacearum	full	None	None
**GCF_002288565.1**	Xanthomonas citri pv. malvacearum	full	None	None

**GCF_002288585.1**	Xanthomonas citri pv. malvacearum	full	None	None
**GCF_009671025.1**	Xanthomonas citri pv. malvacearum	full	None	None
**GCF_003064125.1**	Xanthomonas citri pv. malvacearum	full	None	None
**GCF_002019255.1**	Xanthomonas citri pv. malvacearum	full	None	None
**GCF_001908725.1**	Xanthomonas vesicatoria ATCC 35937	full	None	None
**GCF_000192025.1**	Xanthomonas vesicatoria ATCC 35937	full	None	None
**GCF_002019225.1**	Xanthomonas cissicola	full	None	full
**GCF_008801575.1**	Xanthomonas cissicola	full	None	full
**GCF_002218265.1**	Xanthomonas citri pv. vignicola	full	partial	None
**GCF_002218245.1**	Xanthomonas citri pv. vignicola	full	None	None
**GCF_002218285.1**	Xanthomonas citri pv. vignicola	full	partial	None
**GCF_002285515.1**	Xanthomonas hortorum	None	None	None
**GCF_008728175.1**	Xanthomonas hortorum	None	None	None
**GCF_011305375.1**	Xanthomonas hortorum	None	None	None
**GCF_021353055.1**	Xanthomonas hortorum	None	None	full
**GCF_002846195.1**	Xanthomonas prunicola	partial	None	None
**GCF_002846205.1**	Xanthomonas prunicola	partial	None	None
**GCF_002846225.1**	Xanthomonas prunicola	partial	None	None
**GCF_002939785.1**	Xanthomonas codiaei	full	partial	None
**GCF_020879255.1**	Xanthomonas codiaei	full	full	None
**GCF_002939865.1**	Xanthomonas dyei	partial	partial	None
**GCF_003363875.1**	Xanthomonas dyei	full	full	None
**GCF_003363885.1**	Xanthomonas dyei	full	None	None
**GCF_002940015.1**	Xanthomonas melonis	full	None	None
**GCF_020783655.1**	Xanthomonas melonis	full	None	None
**GCF_020990835.1**	Xanthomonas melonis	full	partial	None
**GCF_020990825.1**	Xanthomonas melonis	full	partial	None
**GCF_020990865.1**	Xanthomonas melonis	full	partial	None
**GCF_020990895.1**	Xanthomonas melonis	full	partial	None
**GCF_002940045.1**	Xanthomonas pisi	partial	partial	None
**GCF_002940065.1**	Xanthomonas populi	None	None	partial
**GCF_002940085.1**	Xanthomonas sacchari	None	None	None
**GCF_000815185.1**	Xanthomonas sacchari	None	None	None
**GCF_000831625.1**	Xanthomonas sacchari	None	None	None
**GCF_014206815.1**	Xanthomonas sacchari	None	None	None
**GCF_003015715.1**	Xanthomonas vasicola pv. vasculorum	partial	full	None
**GCF_003949975.1**	Xanthomonas vasicola pv. vasculorum	partial	full	None
**GCF_002191955.1**	Xanthomonas vasicola pv. vasculorum	partial	full	None
**GCF_002490275.1**	Xanthomonas vasicola pv. vasculorum	partial	full	None
**GCF_003116615.1**	Xanthomonas vasicola pv. vasculorum	partial	full	None
**GCF_003116635.1**	Xanthomonas vasicola pv. vasculorum	partial	full	None

**GCF_003116655.1**	Xanthomonas vasicola pv. vasculorum	partial	full	None
**GCF_003724955.1**	Xanthomonas vasicola pv. vasculorum	partial	full	None
**GCF_003725315.1**	Xanthomonas vasicola pv. vasculorum	partial	full	None
**GCF_019200945.1**	Xanthomonas vasicola pv. vasculorum	partial	full	None
**GCF_019209805.1**	Xanthomonas vasicola pv. vasculorum	partial	full	None
**GCF_002191965.1**	Xanthomonas vasicola pv. vasculorum	partial	full	None
**GCF_003724915.1**	Xanthomonas vasicola pv. vasculorum	partial	full	None
**GCF_003724975.1**	Xanthomonas vasicola pv. vasculorum	partial	full	None
**GCF_003724995.1**	Xanthomonas vasicola pv. vasculorum	partial	full	None
**GCF_003725005.1**	Xanthomonas vasicola pv. vasculorum	partial	full	None
**GCF_003725035.1**	Xanthomonas vasicola pv. vasculorum	partial	full	None
**GCF_003725055.1**	Xanthomonas vasicola pv. vasculorum	partial	full	None
**GCF_003725075.1**	Xanthomonas vasicola pv. vasculorum	partial	full	None
**GCF_003725095.1**	Xanthomonas vasicola pv. vasculorum	partial	full	None
**GCF_003725115.1**	Xanthomonas vasicola pv. vasculorum	partial	full	None
**GCF_003725135.1**	Xanthomonas vasicola pv. vasculorum	partial	full	None
**GCF_003725155.1**	Xanthomonas vasicola pv. vasculorum	partial	full	None
**GCF_003725165.1**	Xanthomonas vasicola pv. vasculorum	partial	full	None
**GCF_003725195.1**	Xanthomonas vasicola pv. vasculorum	partial	full	None
**GCF_003725215.1**	Xanthomonas vasicola pv. vasculorum	partial	full	None
**GCF_003725235.1**	Xanthomonas vasicola pv. vasculorum	partial	full	None
**GCF_003725255.1**	Xanthomonas vasicola pv. vasculorum	partial	full	None
**GCF_003725265.1**	Xanthomonas vasicola pv. vasculorum	partial	full	None
**GCF_003725875.1**	Xanthomonas vasicola pv. vasculorum	partial	full	None
**GCF_003725915.1**	Xanthomonas vasicola pv. vasculorum	partial	full	None
**GCF_003725935.1**	Xanthomonas vasicola pv. vasculorum	partial	full	None
**GCF_010279765.1**	Xanthomonas vasicola pv. vasculorum	partial	full	None
**GCF_003999485.1**	Xanthomonas citri pv. fuscans	full	partial	full
**GCF_003999505.1**	Xanthomonas citri pv. fuscans	full	partial	full
**GCF_004000475.1**	Xanthomonas citri pv. fuscans	full	partial	full
**GCF_018831285.1**	Xanthomonas citri pv. fuscans	full	partial	None
**GCF_018831325.1**	Xanthomonas citri pv. fuscans	full	partial	None
**GCF_018831385.1**	Xanthomonas citri pv. fuscans	full	partial	None
**GCF_000969685.2**	Xanthomonas citri pv. fuscans	full	partial	None
**GCF_017742815.1**	Xanthomonas citri pv. fuscans	full	partial	None
**GCF_000788075.1**	Xanthomonas citri pv. fuscans	full	partial	None
**GCF_001401605.1**	Xanthomonas citri pv. fuscans	full	partial	None
**GCF_002309515.1**	Xanthomonas citri pv. fuscans	full	partial	None
**GCF_000741885.1**	Xanthomonas citri pv. fuscans	full	partial	None
**GCF_000764875.1**	Xanthomonas citri pv. fuscans	full	partial	None
**GCF_000774025.2**	Xanthomonas citri pv. fuscans	full	partial	None
**GCF_000775185.1**	Xanthomonas citri pv. fuscans	full	partial	None

**GCF_000775195.1**	Xanthomonas citri pv. fuscans	full	partial	None
**GCF_000775215.1**	Xanthomonas citri pv. fuscans	full	partial	None
**GCF_000786925.1**	Xanthomonas citri pv. fuscans	full	partial	None
**GCF_000786935.1**	Xanthomonas citri pv. fuscans	full	partial	None
**GCF_000786945.1**	Xanthomonas citri pv. fuscans	full	partial	None
**GCF_000817715.3**	Xanthomonas citri pv. fuscans	full	partial	None
**GCF_000827985.2**	Xanthomonas citri pv. fuscans	full	partial	None
**GCF_020879395.1**	Xanthomonas citri pv. fuscans	full	None	None
**GCF_020879475.1**	Xanthomonas citri pv. fuscans	full	partial	None
**GCF_900234415.1**	Xanthomonas citri pv. fuscans	full	partial	None
**GCF_900234455.1**	Xanthomonas citri pv. fuscans	full	partial	None
**GCF_900234465.1**	Xanthomonas citri pv. fuscans	full	partial	full
**GCF_900234475.1**	Xanthomonas citri pv. fuscans	full	partial	full
**GCF_900234495.1**	Xanthomonas citri pv. fuscans	full	partial	full
**GCF_900234505.1**	Xanthomonas citri pv. fuscans	full	partial	None
**GCF_900234515.1**	Xanthomonas citri pv. fuscans	full	partial	None
**GCF_900234565.1**	Xanthomonas citri pv. fuscans	full	None	None
**GCF_900234485.1**	Xanthomonas citri pv. fuscans	full	partial	None
**GCF_004136375.1**	Xanthomonas oryzae pv. oryzae	partial	full	full
**GCF_001518895.1**	Xanthomonas oryzae pv. oryzae	partial	full	full
**GCF_001746595.1**	Xanthomonas oryzae pv. oryzae	partial	full	full
**GCF_001746615.1**	Xanthomonas oryzae pv. oryzae	partial	full	full
**GCF_001746635.1**	Xanthomonas oryzae pv. oryzae	partial	full	full
**GCF_001746655.1**	Xanthomonas oryzae pv. oryzae	partial	full	full
**GCF_001746675.1**	Xanthomonas oryzae pv. oryzae	partial	full	full
**GCF_001746695.1**	Xanthomonas oryzae pv. oryzae	partial	full	full
**GCF_001746715.1**	Xanthomonas oryzae pv. oryzae	partial	full	full
**GCF_001746735.1**	Xanthomonas oryzae pv. oryzae	partial	full	full
**GCF_001929095.2**	Xanthomonas oryzae pv. oryzae	partial	full	full
**GCF_001929235.2**	Xanthomonas oryzae pv. oryzae	partial	full	full
**GCF_002023005.1**	Xanthomonas oryzae pv. oryzae	partial	full	full
**GCF_002850075.1**	Xanthomonas oryzae pv. oryzae	partial	full	full
**GCF_002850095.1**	Xanthomonas oryzae pv. oryzae	partial	full	full
**GCF_002850115.1**	Xanthomonas oryzae pv. oryzae	partial	full	full
**GCF_002850135.1**	Xanthomonas oryzae pv. oryzae	partial	full	full
**GCF_002850155.1**	Xanthomonas oryzae pv. oryzae	partial	full	full
**GCF_002850175.1**	Xanthomonas oryzae pv. oryzae	partial	full	full
**GCF_002850195.1**	Xanthomonas oryzae pv. oryzae	partial	full	full
**GCF_002850215.1**	Xanthomonas oryzae pv. oryzae	partial	full	full
**GCF_002895725.2**	Xanthomonas oryzae pv. oryzae	partial	full	full
**GCF_003031365.1**	Xanthomonas oryzae pv. oryzae	partial	full	full

**GCF_003031385.1**	Xanthomonas oryzae pv. oryzae	partial	full	full
**GCF_003382775.1**	Xanthomonas oryzae pv. oryzae	partial	full	full
**GCF_003382795.1**	Xanthomonas oryzae pv. oryzae	partial	full	full
**GCF_003382815.1**	Xanthomonas oryzae pv. oryzae	partial	full	full
**GCF_003382835.1**	Xanthomonas oryzae pv. oryzae	partial	full	full
**GCF_003382855.1**	Xanthomonas oryzae pv. oryzae	partial	full	full
**GCF_003382895.1**	Xanthomonas oryzae pv. oryzae	partial	full	full
**GCF_003382915.1**	Xanthomonas oryzae pv. oryzae	partial	full	full
**GCF_003382935.1**	Xanthomonas oryzae pv. oryzae	partial	full	full
**GCF_003427055.1**	Xanthomonas oryzae pv. oryzae	partial	full	full
**GCF_003428965.1**	Xanthomonas oryzae pv. oryzae	partial	full	full
**GCF_003522605.1**	Xanthomonas oryzae pv. oryzae	partial	full	full
**GCF_003932055.1**	Xanthomonas oryzae pv. oryzae	partial	full	full
**GCF_004322815.1**	Xanthomonas oryzae pv. oryzae	partial	full	full
**GCF_004322835.1**	Xanthomonas oryzae pv. oryzae	partial	full	full
**GCF_004355285.1**	Xanthomonas oryzae pv. oryzae	partial	full	full
**GCF_004355305.1**	Xanthomonas oryzae pv. oryzae	partial	full	full
**GCF_004355325.1**	Xanthomonas oryzae pv. oryzae	partial	full	full
**GCF_004355365.1**	Xanthomonas oryzae pv. oryzae	partial	full	full
**GCF_004355385.1**	Xanthomonas oryzae pv. oryzae	partial	full	full
**GCF_004355405.1**	Xanthomonas oryzae pv. oryzae	partial	full	full
**GCF_004355425.1**	Xanthomonas oryzae pv. oryzae	partial	full	full
**GCF_004355445.1**	Xanthomonas oryzae pv. oryzae	partial	full	full
**GCF_004355485.1**	Xanthomonas oryzae pv. oryzae	partial	full	full
**GCF_004355505.1**	Xanthomonas oryzae pv. oryzae	partial	full	full
**GCF_004355525.1**	Xanthomonas oryzae pv. oryzae	partial	full	full
**GCF_004355545.1**	Xanthomonas oryzae pv. oryzae	partial	full	full
**GCF_004355565.1**	Xanthomonas oryzae pv. oryzae	partial	full	full
**GCF_004355585.1**	Xanthomonas oryzae pv. oryzae	partial	full	full
**GCF_004355605.1**	Xanthomonas oryzae pv. oryzae	partial	full	full
**GCF_004355705.1**	Xanthomonas oryzae pv. oryzae	partial	full	full
**GCF_004355725.1**	Xanthomonas oryzae pv. oryzae	partial	full	full
**GCF_004355745.1**	Xanthomonas oryzae pv. oryzae	partial	full	full
**GCF_004355765.2**	Xanthomonas oryzae pv. oryzae	partial	full	full
**GCF_004355785.1**	Xanthomonas oryzae pv. oryzae	partial	full	full
**GCF_004355805.1**	Xanthomonas oryzae pv. oryzae	partial	full	full
**GCF_004355825.1**	Xanthomonas oryzae pv. oryzae	partial	full	full
**GCF_004355845.1**	Xanthomonas oryzae pv. oryzae	partial	full	full
**GCF_004355865.1**	Xanthomonas oryzae pv. oryzae	partial	full	full
**GCF_004355885.3**	Xanthomonas oryzae pv. oryzae	partial	full	full
**GCF_011045315.1**	Xanthomonas oryzae pv. oryzae	partial	full	full
**GCF_011604745.1**	Xanthomonas oryzae pv. oryzae	partial	full	full

**GCF_011604765.1**	Xanthomonas oryzae pv. oryzae	partial	full	full
**GCF_011604785.1**	Xanthomonas oryzae pv. oryzae	partial	full	full
**GCF_017577225.1**	Xanthomonas oryzae pv. oryzae	partial	full	full
**GCF_018288775.1**	Xanthomonas oryzae pv. oryzae	partial	full	full
**GCF_019915985.1**	Xanthomonas oryzae pv. oryzae	partial	full	full
**GCF_020790135.1**	Xanthomonas oryzae pv. oryzae	partial	full	full
**GCF_020866785.1**	Xanthomonas oryzae pv. oryzae	partial	full	full
**GCF_021228635.1**	Xanthomonas oryzae pv. oryzae	partial	full	full
**GCF_001466505.1**	Xanthomonas oryzae pv. oryzae	partial	full	full
**GCF_004355345.1**	Xanthomonas oryzae pv. oryzae	partial	full	full
**GCF_004355465.1**	Xanthomonas oryzae pv. oryzae	partial	full	full
**GCF_002895665.1**	Xanthomonas oryzae pv. oryzae	partial	full	full
**GCF_003295455.1**	Xanthomonas oryzae pv. oryzae	partial	full	full
**GCF_003295485.1**	Xanthomonas oryzae pv. oryzae	partial	full	full
**GCF_003295515.1**	Xanthomonas oryzae pv. oryzae	partial	full	full
**GCF_003295525.1**	Xanthomonas oryzae pv. oryzae	partial	full	full
**GCF_003296105.1**	Xanthomonas oryzae pv. oryzae	partial	full	full
**GCF_003297235.1**	Xanthomonas oryzae pv. oryzae	partial	full	full
**GCF_003297275.1**	Xanthomonas oryzae pv. oryzae	partial	full	full
**GCF_003297285.1**	Xanthomonas oryzae pv. oryzae	partial	full	full
**GCF_003297375.1**	Xanthomonas oryzae pv. oryzae	partial	full	full
**GCF_003297395.1**	Xanthomonas oryzae pv. oryzae	partial	full	full
**GCF_003297685.1**	Xanthomonas oryzae pv. oryzae	partial	full	full
**GCF_003298095.1**	Xanthomonas oryzae pv. oryzae	partial	full	full
**GCF_003298365.1**	Xanthomonas oryzae pv. oryzae	partial	full	full
**GCF_003299275.1**	Xanthomonas oryzae pv. oryzae	partial	full	full
**GCF_004299365.1**	Xanthomonas oryzae pv. oryzae	partial	full	full
**GCF_004299395.1**	Xanthomonas oryzae pv. oryzae	partial	full	full
**GCF_004321555.1**	Xanthomonas oryzae pv. oryzae	partial	full	full
**GCF_002895745.1**	Xanthomonas oryzae pv. oryzae	partial	full	full
**GCF_002895775.1**	Xanthomonas oryzae pv. oryzae	partial	full	full
**GCF_002895825.1**	Xanthomonas oryzae pv. oryzae	partial	full	full
**GCF_003294085.1**	Xanthomonas oryzae pv. oryzae	partial	full	full
**GCF_003294295.1**	Xanthomonas oryzae pv. oryzae	partial	full	full
**GCF_003294335.1**	Xanthomonas oryzae pv. oryzae	partial	full	full
**GCF_003294345.1**	Xanthomonas oryzae pv. oryzae	partial	full	full
**GCF_003294365.1**	Xanthomonas oryzae pv. oryzae	partial	full	full
**GCF_003294385.1**	Xanthomonas oryzae pv. oryzae	partial	full	full
**GCF_003294405.1**	Xanthomonas oryzae pv. oryzae	partial	full	full
**GCF_003294425.1**	Xanthomonas oryzae pv. oryzae	partial	full	full
**GCF_003294445.1**	Xanthomonas oryzae pv. oryzae	partial	full	full

**GCF_003294465.1**	Xanthomonas oryzae pv. oryzae	partial	full	full
**GCF_003294495.1**	Xanthomonas oryzae pv. oryzae	partial	full	full
**GCF_003294505.1**	Xanthomonas oryzae pv. oryzae	partial	full	full
**GCF_003294525.1**	Xanthomonas oryzae pv. oryzae	partial	full	full
**GCF_003294545.1**	Xanthomonas oryzae pv. oryzae	partial	full	full
**GCF_003294715.1**	Xanthomonas oryzae pv. oryzae	partial	full	full
**GCF_003294835.1**	Xanthomonas oryzae pv. oryzae	partial	full	full
**GCF_003294875.1**	Xanthomonas oryzae pv. oryzae	partial	full	full
**GCF_003294955.1**	Xanthomonas oryzae pv. oryzae	partial	full	full
**GCF_003294995.1**	Xanthomonas oryzae pv. oryzae	partial	full	full
**GCF_003295045.1**	Xanthomonas oryzae pv. oryzae	partial	full	full
**GCF_003295065.1**	Xanthomonas oryzae pv. oryzae	partial	full	full
**GCF_003295075.1**	Xanthomonas oryzae pv. oryzae	partial	full	full
**GCF_003295125.1**	Xanthomonas oryzae pv. oryzae	partial	full	full
**GCF_003295175.1**	Xanthomonas oryzae pv. oryzae	partial	full	full
**GCF_003295185.1**	Xanthomonas oryzae pv. oryzae	partial	full	full
**GCF_003295245.1**	Xanthomonas oryzae pv. oryzae	partial	full	full
**GCF_003295315.1**	Xanthomonas oryzae pv. oryzae	partial	full	full
**GCF_003295335.1**	Xanthomonas oryzae pv. oryzae	partial	full	full
**GCF_003295365.1**	Xanthomonas oryzae pv. oryzae	partial	full	full
**GCF_003295375.1**	Xanthomonas oryzae pv. oryzae	partial	full	full
**GCF_003295545.1**	Xanthomonas oryzae pv. oryzae	partial	full	full
**GCF_003295565.1**	Xanthomonas oryzae pv. oryzae	partial	full	full
**GCF_003295585.1**	Xanthomonas oryzae pv. oryzae	partial	full	full
**GCF_003295645.1**	Xanthomonas oryzae pv. oryzae	partial	full	full
**GCF_003295705.1**	Xanthomonas oryzae pv. oryzae	partial	full	full
**GCF_003295725.1**	Xanthomonas oryzae pv. oryzae	partial	full	full
**GCF_003295735.1**	Xanthomonas oryzae pv. oryzae	partial	full	full
**GCF_003295775.1**	Xanthomonas oryzae pv. oryzae	partial	full	full
**GCF_003295795.1**	Xanthomonas oryzae pv. oryzae	partial	full	full
**GCF_003295805.1**	Xanthomonas oryzae pv. oryzae	partial	full	full
**GCF_003295915.1**	Xanthomonas oryzae pv. oryzae	partial	full	full
**GCF_003295925.1**	Xanthomonas oryzae pv. oryzae	partial	full	full
**GCF_003295955.1**	Xanthomonas oryzae pv. oryzae	partial	full	full
**GCF_003295965.1**	Xanthomonas oryzae pv. oryzae	partial	full	full
**GCF_003296015.1**	Xanthomonas oryzae pv. oryzae	partial	full	full
**GCF_003296065.1**	Xanthomonas oryzae pv. oryzae	partial	full	full
**GCF_003296115.1**	Xanthomonas oryzae pv. oryzae	partial	full	full
**GCF_003296245.1**	Xanthomonas oryzae pv. oryzae	partial	full	full
**GCF_003296255.1**	Xanthomonas oryzae pv. oryzae	partial	full	full
**GCF_003296305.1**	Xanthomonas oryzae pv. oryzae	partial	full	full
**GCF_003296365.1**	Xanthomonas oryzae pv. oryzae	partial	full	full

**GCF_003296405.1**	Xanthomonas oryzae pv. oryzae	partial	full	full
**GCF_003296415.1**	Xanthomonas oryzae pv. oryzae	partial	full	full
**GCF_003296455.1**	Xanthomonas oryzae pv. oryzae	partial	full	full
**GCF_003296465.1**	Xanthomonas oryzae pv. oryzae	partial	full	full
**GCF_003296495.1**	Xanthomonas oryzae pv. oryzae	partial	full	full
**GCF_003296505.1**	Xanthomonas oryzae pv. oryzae	partial	full	full
**GCF_003296515.1**	Xanthomonas oryzae pv. oryzae	partial	full	full
**GCF_003296565.1**	Xanthomonas oryzae pv. oryzae	partial	full	full
**GCF_003296695.1**	Xanthomonas oryzae pv. oryzae	partial	full	full
**GCF_003296765.1**	Xanthomonas oryzae pv. oryzae	partial	full	full
**GCF_003296805.1**	Xanthomonas oryzae pv. oryzae	partial	full	full
**GCF_003296915.1**	Xanthomonas oryzae pv. oryzae	partial	full	full
**GCF_003296925.1**	Xanthomonas oryzae pv. oryzae	partial	full	full
**GCF_003296935.1**	Xanthomonas oryzae pv. oryzae	partial	full	full
**GCF_003297035.1**	Xanthomonas oryzae pv. oryzae	partial	full	full
**GCF_003297095.1**	Xanthomonas oryzae pv. oryzae	partial	full	full
**GCF_003297145.1**	Xanthomonas oryzae pv. oryzae	partial	full	full
**GCF_003297195.1**	Xanthomonas oryzae pv. oryzae	partial	full	full
**GCF_003297505.1**	Xanthomonas oryzae pv. oryzae	partial	full	full
**GCF_003297625.1**	Xanthomonas oryzae pv. oryzae	partial	full	full
**GCF_003297775.1**	Xanthomonas oryzae pv. oryzae	partial	full	full
**GCF_003297785.1**	Xanthomonas oryzae pv. oryzae	partial	full	full
**GCF_003297895.1**	Xanthomonas oryzae pv. oryzae	partial	full	full
**GCF_003297905.1**	Xanthomonas oryzae pv. oryzae	partial	full	full
**GCF_003297915.1**	Xanthomonas oryzae pv. oryzae	partial	full	full
**GCF_003297945.1**	Xanthomonas oryzae pv. oryzae	partial	full	full
**GCF_003298065.1**	Xanthomonas oryzae pv. oryzae	partial	full	full
**GCF_003298075.1**	Xanthomonas oryzae pv. oryzae	partial	full	full
**GCF_003298245.1**	Xanthomonas oryzae pv. oryzae	partial	full	full
**GCF_003298455.1**	Xanthomonas oryzae pv. oryzae	partial	full	full
**GCF_003298965.1**	Xanthomonas oryzae pv. oryzae	partial	full	full
**GCF_003299055.1**	Xanthomonas oryzae pv. oryzae	partial	full	full
**GCF_003299145.1**	Xanthomonas oryzae pv. oryzae	partial	full	full
**GCF_003299525.1**	Xanthomonas oryzae pv. oryzae	partial	full	full
**GCF_003299635.1**	Xanthomonas oryzae pv. oryzae	partial	full	full
**GCF_003299745.1**	Xanthomonas oryzae pv. oryzae	partial	full	full
**GCF_003299755.1**	Xanthomonas oryzae pv. oryzae	partial	full	full
**GCF_003299865.1**	Xanthomonas oryzae pv. oryzae	partial	full	full
**GCF_003299955.1**	Xanthomonas oryzae pv. oryzae	partial	full	full
**GCF_003300055.1**	Xanthomonas oryzae pv. oryzae	partial	full	full
**GCF_003300075.1**	Xanthomonas oryzae pv. oryzae	partial	full	full

**GCF_003300215.1**	Xanthomonas oryzae pv. oryzae	partial	full	full
**GCF_003300225.1**	Xanthomonas oryzae pv. oryzae	partial	full	full
**GCF_003300515.1**	Xanthomonas oryzae pv. oryzae	partial	full	full
**GCF_003300585.1**	Xanthomonas oryzae pv. oryzae	partial	full	full
**GCF_003300845.1**	Xanthomonas oryzae pv. oryzae	partial	full	full
**GCF_001927305.1**	Xanthomonas oryzae pv. oryzae	partial	full	full
**GCF_001927325.1**	Xanthomonas oryzae pv. oryzae	partial	full	full
**GCF_001927335.1**	Xanthomonas oryzae pv. oryzae	partial	full	full
**GCF_001927385.1**	Xanthomonas oryzae pv. oryzae	partial	full	full
**GCF_001927395.1**	Xanthomonas oryzae pv. oryzae	partial	full	full
**GCF_001927415.1**	Xanthomonas oryzae pv. oryzae	partial	full	full
**GCF_001927465.1**	Xanthomonas oryzae pv. oryzae	partial	full	full
**GCF_001927475.1**	Xanthomonas oryzae pv. oryzae	partial	full	full
**GCF_001927495.1**	Xanthomonas oryzae pv. oryzae	partial	full	full
**GCF_001927505.1**	Xanthomonas oryzae pv. oryzae	partial	full	full
**GCF_001927555.1**	Xanthomonas oryzae pv. oryzae	partial	full	full
**GCF_001927575.1**	Xanthomonas oryzae pv. oryzae	partial	full	full
**GCF_001927605.1**	Xanthomonas oryzae pv. oryzae	partial	full	full
**GCF_001927615.1**	Xanthomonas oryzae pv. oryzae	partial	full	full
**GCF_001927625.1**	Xanthomonas oryzae pv. oryzae	partial	full	full
**GCF_001927635.1**	Xanthomonas oryzae pv. oryzae	partial	full	full
**GCF_001927695.1**	Xanthomonas oryzae pv. oryzae	partial	full	full
**GCF_001927705.1**	Xanthomonas oryzae pv. oryzae	partial	full	full
**GCF_001927725.1**	Xanthomonas oryzae pv. oryzae	partial	full	full
**GCF_001927775.1**	Xanthomonas oryzae pv. oryzae	partial	full	full
**GCF_001927785.1**	Xanthomonas oryzae pv. oryzae	partial	full	full
**GCF_001927795.1**	Xanthomonas oryzae pv. oryzae	partial	full	full
**GCF_001927845.1**	Xanthomonas oryzae pv. oryzae	partial	full	full
**GCF_001927855.1**	Xanthomonas oryzae pv. oryzae	partial	full	full
**GCF_001927865.1**	Xanthomonas oryzae pv. oryzae	partial	full	full
**GCF_001927875.1**	Xanthomonas oryzae pv. oryzae	partial	full	full
**GCF_001927925.1**	Xanthomonas oryzae pv. oryzae	partial	full	full
**GCF_001927935.1**	Xanthomonas oryzae pv. oryzae	partial	full	full
**GCF_001927945.1**	Xanthomonas oryzae pv. oryzae	partial	full	full
**GCF_001927955.1**	Xanthomonas oryzae pv. oryzae	partial	full	full
**GCF_001928005.1**	Xanthomonas oryzae pv. oryzae	partial	full	full
**GCF_001928015.1**	Xanthomonas oryzae pv. oryzae	partial	full	full
**GCF_001928025.1**	Xanthomonas oryzae pv. oryzae	partial	full	full
**GCF_001928035.1**	Xanthomonas oryzae pv. oryzae	partial	full	full
**GCF_001928085.1**	Xanthomonas oryzae pv. oryzae	partial	full	full
**GCF_001928095.1**	Xanthomonas oryzae pv. oryzae	partial	full	full
**GCF_001928105.1**	Xanthomonas oryzae pv. oryzae	partial	full	full

**GCF_001928135.1**	Xanthomonas oryzae pv. oryzae	partial	full	full
**GCF_001928165.1**	Xanthomonas oryzae pv. oryzae	partial	full	full
**GCF_001928175.1**	Xanthomonas oryzae pv. oryzae	partial	full	full
**GCF_001928185.1**	Xanthomonas oryzae pv. oryzae	partial	full	full
**GCF_001928245.1**	Xanthomonas oryzae pv. oryzae	partial	full	full
**GCF_001928255.1**	Xanthomonas oryzae pv. oryzae	partial	full	full
**GCF_001928265.1**	Xanthomonas oryzae pv. oryzae	partial	full	full
**GCF_001928325.1**	Xanthomonas oryzae pv. oryzae	partial	full	full
**GCF_001928355.1**	Xanthomonas oryzae pv. oryzae	partial	full	full
**GCF_001928365.1**	Xanthomonas oryzae pv. oryzae	partial	full	full
**GCF_001928405.1**	Xanthomonas oryzae pv. oryzae	partial	full	full
**GCF_001928415.1**	Xanthomonas oryzae pv. oryzae	partial	full	full
**GCF_001928425.1**	Xanthomonas oryzae pv. oryzae	partial	full	full
**GCF_001928445.1**	Xanthomonas oryzae pv. oryzae	partial	full	full
**GCF_001928485.1**	Xanthomonas oryzae pv. oryzae	partial	full	full
**GCF_001928495.1**	Xanthomonas oryzae pv. oryzae	partial	full	full
**GCF_001928515.1**	Xanthomonas oryzae pv. oryzae	partial	full	full
**GCF_001928525.1**	Xanthomonas oryzae pv. oryzae	partial	full	full
**GCF_001928565.1**	Xanthomonas oryzae pv. oryzae	partial	full	full
**GCF_001928585.1**	Xanthomonas oryzae pv. oryzae	partial	full	full
**GCF_001928605.1**	Xanthomonas oryzae pv. oryzae	partial	full	full
**GCF_001928615.1**	Xanthomonas oryzae pv. oryzae	partial	full	full
**GCF_001928625.1**	Xanthomonas oryzae pv. oryzae	partial	full	full
**GCF_001928665.1**	Xanthomonas oryzae pv. oryzae	partial	full	full
**GCF_001928675.1**	Xanthomonas oryzae pv. oryzae	partial	full	full
**GCF_001928685.1**	Xanthomonas oryzae pv. oryzae	partial	full	full
**GCF_001928745.1**	Xanthomonas oryzae pv. oryzae	partial	full	full
**GCF_001928755.1**	Xanthomonas oryzae pv. oryzae	partial	full	full
**GCF_001928765.1**	Xanthomonas oryzae pv. oryzae	partial	full	full
**GCF_001928775.1**	Xanthomonas oryzae pv. oryzae	partial	full	full
**GCF_001928825.1**	Xanthomonas oryzae pv. oryzae	partial	full	full
**GCF_001928835.1**	Xanthomonas oryzae pv. oryzae	partial	full	full
**GCF_001928845.1**	Xanthomonas oryzae pv. oryzae	partial	full	full
**GCF_001928855.1**	Xanthomonas oryzae pv. oryzae	partial	full	full
**GCF_001928905.1**	Xanthomonas oryzae pv. oryzae	partial	full	full
**GCF_001928915.1**	Xanthomonas oryzae pv. oryzae	partial	full	full
**GCF_001928935.1**	Xanthomonas oryzae pv. oryzae	partial	full	full
**GCF_001928945.1**	Xanthomonas oryzae pv. oryzae	partial	full	full
**GCF_001928985.1**	Xanthomonas oryzae pv. oryzae	partial	full	full
**GCF_001928995.1**	Xanthomonas oryzae pv. oryzae	partial	full	full
**GCF_001929005.1**	Xanthomonas oryzae pv. oryzae	partial	full	full

**GCF_001929015.1**	Xanthomonas oryzae pv. oryzae	partial	full	partial
**GCF_001929065.1**	Xanthomonas oryzae pv. oryzae	partial	full	full
**GCF_001929075.1**	Xanthomonas oryzae pv. oryzae	partial	full	full
**GCF_001929085.1**	Xanthomonas oryzae pv. oryzae	partial	full	full
**GCF_001929145.1**	Xanthomonas oryzae pv. oryzae	partial	full	full
**GCF_001929155.1**	Xanthomonas oryzae pv. oryzae	partial	full	full
**GCF_001929165.1**	Xanthomonas oryzae pv. oryzae	partial	full	full
**GCF_001929185.1**	Xanthomonas oryzae pv. oryzae	partial	full	full
**GCF_001929225.1**	Xanthomonas oryzae pv. oryzae	partial	full	full
**GCF_001929245.1**	Xanthomonas oryzae pv. oryzae	partial	full	full
**GCF_001929255.1**	Xanthomonas oryzae pv. oryzae	partial	full	full
**GCF_001974485.1**	Xanthomonas oryzae pv. oryzae	partial	full	full
**GCF_002895805.1**	Xanthomonas oryzae pv. oryzae	partial	full	full
**GCF_003295275.1**	Xanthomonas oryzae pv. oryzae	partial	full	full
**GCF_003297745.1**	Xanthomonas oryzae pv. oryzae	partial	full	full
**GCF_003297765.1**	Xanthomonas oryzae pv. oryzae	partial	full	full
**GCF_003298055.1**	Xanthomonas oryzae pv. oryzae	partial	full	full
**GCF_003298825.1**	Xanthomonas oryzae pv. oryzae	partial	full	full
**GCF_003299625.1**	Xanthomonas oryzae pv. oryzae	partial	full	full
**GCF_003300065.1**	Xanthomonas oryzae pv. oryzae	partial	full	full
**GCF_003300125.1**	Xanthomonas oryzae pv. oryzae	partial	full	full
**GCF_003382265.1**	Xanthomonas oryzae pv. oryzae	partial	full	full
**GCF_001927315.1**	Xanthomonas oryzae pv. oryzae	partial	full	full
**GCF_001927545.1**	Xanthomonas oryzae pv. oryzae	partial	full	full
**GCF_001927685.1**	Xanthomonas oryzae pv. oryzae	partial	full	full
**GCF_001927765.1**	Xanthomonas oryzae pv. oryzae	partial	full	full
**GCF_001928285.1**	Xanthomonas oryzae pv. oryzae	partial	full	full
**GCF_001928345.1**	Xanthomonas oryzae pv. oryzae	partial	full	full
**GCF_001928695.1**	Xanthomonas oryzae pv. oryzae	partial	full	full
**GCF_003294095.1**	Xanthomonas oryzae pv. oryzae	partial	partial	partial
**GCF_003294115.1**	Xanthomonas oryzae pv. oryzae	partial	partial	full
**GCF_003294135.1**	Xanthomonas oryzae pv. oryzae	partial	partial	partial
**GCF_003294155.1**	Xanthomonas oryzae pv. oryzae	partial	partial	partial
**GCF_003294195.1**	Xanthomonas oryzae pv. oryzae	partial	partial	partial
**GCF_003294205.1**	Xanthomonas oryzae pv. oryzae	partial	partial	full
**GCF_003294225.1**	Xanthomonas oryzae pv. oryzae	partial	partial	full
**GCF_003294275.1**	Xanthomonas oryzae pv. oryzae	partial	full	full
**GCF_003294285.1**	Xanthomonas oryzae pv. oryzae	partial	partial	partial
**GCF_003294575.1**	Xanthomonas oryzae pv. oryzae	partial	full	full
**GCF_003294665.1**	Xanthomonas oryzae pv. oryzae	partial	full	full
**GCF_003294695.1**	Xanthomonas oryzae pv. oryzae	partial	partial	partial
**GCF_003294765.1**	Xanthomonas oryzae pv. oryzae	partial	partial	partial

**GCF_003295135.1**	Xanthomonas oryzae pv. oryzae	partial	partial	partial
**GCF_003295235.1**	Xanthomonas oryzae pv. oryzae	partial	full	partial
**GCF_003295405.1**	Xanthomonas oryzae pv. oryzae	partial	partial	partial
**GCF_003295625.1**	Xanthomonas oryzae pv. oryzae	partial	partial	full
**GCF_003295635.1**	Xanthomonas oryzae pv. oryzae	partial	full	full
**GCF_003295825.1**	Xanthomonas oryzae pv. oryzae	partial	partial	partial
**GCF_003295845.1**	Xanthomonas oryzae pv. oryzae	partial	partial	partial
**GCF_003295855.1**	Xanthomonas oryzae pv. oryzae	partial	partial	partial
**GCF_003295975.1**	Xanthomonas oryzae pv. oryzae	partial	full	full
**GCF_003296135.1**	Xanthomonas oryzae pv. oryzae	partial	partial	partial
**GCF_003296555.1**	Xanthomonas oryzae pv. oryzae	partial	partial	partial
**GCF_003296575.1**	Xanthomonas oryzae pv. oryzae	partial	full	partial
**GCF_003296615.1**	Xanthomonas oryzae pv. oryzae	partial	full	full
**GCF_003296625.1**	Xanthomonas oryzae pv. oryzae	partial	full	full
**GCF_003296655.1**	Xanthomonas oryzae pv. oryzae	partial	partial	full
**GCF_003296675.1**	Xanthomonas oryzae pv. oryzae	partial	full	full
**GCF_003296775.1**	Xanthomonas oryzae pv. oryzae	partial	partial	partial
**GCF_003296845.1**	Xanthomonas oryzae pv. oryzae	partial	partial	partial
**GCF_003296855.1**	Xanthomonas oryzae pv. oryzae	partial	partial	partial
**GCF_003296865.1**	Xanthomonas oryzae pv. oryzae	partial	full	full
**GCF_003297025.1**	Xanthomonas oryzae pv. oryzae	partial	full	partial
**GCF_003297085.1**	Xanthomonas oryzae pv. oryzae	partial	full	full
**GCF_003297135.1**	Xanthomonas oryzae pv. oryzae	partial	partial	full
**GCF_003297175.1**	Xanthomonas oryzae pv. oryzae	partial	partial	partial
**GCF_003297245.1**	Xanthomonas oryzae pv. oryzae	partial	partial	partial
**GCF_003297475.1**	Xanthomonas oryzae pv. oryzae	partial	partial	partial
**GCF_003297605.1**	Xanthomonas oryzae pv. oryzae	partial	partial	partial
**GCF_003298815.1**	Xanthomonas oryzae pv. oryzae	partial	partial	full
**GCF_003298955.1**	Xanthomonas oryzae pv. oryzae	partial	partial	full
**GCF_003299065.1**	Xanthomonas oryzae pv. oryzae	partial	partial	partial
**GCF_003299075.1**	Xanthomonas oryzae pv. oryzae	partial	full	full
**GCF_003299175.1**	Xanthomonas oryzae pv. oryzae	partial	partial	partial
**GCF_003299225.1**	Xanthomonas oryzae pv. oryzae	partial	full	full
**GCF_003299365.1**	Xanthomonas oryzae pv. oryzae	partial	full	full
**GCF_003299395.1**	Xanthomonas oryzae pv. oryzae	partial	partial	full
**GCF_003299665.1**	Xanthomonas oryzae pv. oryzae	partial	partial	partial
**GCF_003299885.1**	Xanthomonas oryzae pv. oryzae	partial	full	full
**GCF_003300235.1**	Xanthomonas oryzae pv. oryzae	partial	full	full
**GCF_003300265.1**	Xanthomonas oryzae pv. oryzae	partial	full	full
**GCF_003300385.1**	Xanthomonas oryzae pv. oryzae	partial	partial	full
**GCF_003300615.1**	Xanthomonas oryzae pv. oryzae	partial	partial	partial

**GCF_003300685.1**	Xanthomonas oryzae pv. oryzae	partial	partial	full
**GCF_003300715.1**	Xanthomonas oryzae pv. oryzae	partial	full	full
**GCF_003301345.1**	Xanthomonas oryzae pv. oryzae	partial	partial	partial
**GCF_003311715.1**	Xanthomonas oryzae pv. oryzae	partial	full	full
**GCF_006228425.1**	Xanthomonas citri pv. punicae	full	None	None
**GCF_021379025.1**	Xanthomonas citri pv. punicae	full	None	None
**GCF_014858165.1**	Xanthomonas citri pv. punicae	full	None	None
**GCF_007567665.1**	Xanthomonas citri pv. glycines	full	None	full
**GCF_007723825.1**	Xanthomonas citri pv. glycines	full	None	None
**GCF_007724025.1**	Xanthomonas citri pv. glycines	full	None	None
**GCF_007724385.1**	Xanthomonas citri pv. glycines	full	None	None
**GCF_008593565.1**	Xanthomonas citri pv. glycines	full	None	None
**GCF_017723895.1**	Xanthomonas citri pv. glycines	full	None	full
**GCF_002019125.1**	Xanthomonas citri pv. glycines	full	None	None
**GCF_001753585.1**	Xanthomonas citri pv. glycines	full	None	None
**GCF_007559225.1**	Xanthomonas citri pv. glycines	full	None	None
**GCF_007559245.1**	Xanthomonas citri pv. glycines	full	None	None
**GCF_009192945.1**	Xanthomonas maliensis	full	full	full
**GCF_000401255.1**	Xanthomonas maliensis	full	full	full
**GCF_009649705.1**	Xanthomonas sontii	None	None	None
**GCF_008119715.1**	Xanthomonas sontii	None	None	None
**GCF_008119705.1**	Xanthomonas sontii	None	None	None
**GCF_008705275.1**	Xanthomonas sontii	None	None	None
**GCF_009649775.1**	Xanthomonas sontii	None	None	None
**GCF_009769165.1**	Xanthomonas hyacinthi	None	None	None
**GCF_002939895.1**	Xanthomonas hyacinthi	None	None	None
**GCF_009883735.1**	Xanthomonas cucurbitae	partial	partial	None
**GCF_002939885.1**	Xanthomonas cucurbitae	partial	partial	None
**GCF_009931595.1**	Xanthomonas albilineans	None	None	None
**GCF_002939705.1**	Xanthomonas albilineans	None	None	None
**GCF_000962915.1**	Xanthomonas albilineans	None	None	None
**GCF_000962925.1**	Xanthomonas albilineans	None	None	None
**GCF_000962935.1**	Xanthomonas albilineans	None	None	None
**GCF_000962945.1**	Xanthomonas albilineans	None	None	None
**GCF_000962995.1**	Xanthomonas albilineans	None	None	None
**GCF_000963025.1**	Xanthomonas albilineans	None	None	None
**GCF_000963055.1**	Xanthomonas albilineans	None	None	None
**GCF_000963065.1**	Xanthomonas albilineans	None	None	None
**GCF_000963075.1**	Xanthomonas albilineans	None	None	None
**GCF_000963115.1**	Xanthomonas albilineans	None	None	None
**GCF_000963135.1**	Xanthomonas albilineans	None	None	None
**GCF_000963145.1**	Xanthomonas albilineans	None	None	None

**GCF_000963155.1**	Xanthomonas albilineans	None	None	None
**GCF_000963195.1**	Xanthomonas albilineans	None	None	None
**GCF_012848175.1**	Xanthomonas campestris pv. badrii	full	None	None
**GCF_013177355.1**	Xanthomonas axonopodis pv. vasculorum	partial	full	None
**GCF_000724905.1**	Xanthomonas axonopodis pv. vasculorum	partial	full	None
**GCF_002939725.1**	Xanthomonas axonopodis pv. vasculorum	partial	full	None
**GCF_013388375.1**	Xanthomonas campestris pv. raphani	partial	None	None
**GCF_000590355.1**	Xanthomonas campestris pv. raphani	partial	None	None
**GCF_003410105.1**	Xanthomonas campestris pv. raphani	partial	None	None
**GCF_014236795.1**	Xanthomonas theicola	None	None	None
**GCF_002940605.1**	Xanthomonas theicola	None	None	None
**GCF_015243835.1**	Xanthomonas citri	full	None	None
**GCF_016495605.1**	Xanthomonas citri	full	None	None
**GCF_018831345.1**	Xanthomonas citri	full	partial	full
**GCF_018831365.1**	Xanthomonas citri	full	partial	None
**GCF_019434355.1**	Xanthomonas citri	full	None	None
**GCF_001401545.2**	Xanthomonas citri	full	None	None
**GCF_002899435.1**	Xanthomonas citri	full	None	None
**GCF_012922295.1**	Xanthomonas citri	full	None	None
**GCF_016094595.1**	Xanthomonas citri	full	None	None
**GCF_016094615.1**	Xanthomonas citri	full	None	None
**GCF_020783685.1**	Xanthomonas citri	full	None	None
**GCF_017301775.1**	Xanthomonas translucens pv. undulosa	partial	full	full
**GCF_001021935.1**	Xanthomonas translucens pv. undulosa	partial	full	full
**GCF_003050685.1**	Xanthomonas translucens pv. undulosa	partial	full	full
**GCF_008330965.1**	Xanthomonas translucens pv. undulosa	partial	full	full
**GCF_008365355.1**	Xanthomonas translucens pv. undulosa	partial	full	full
**GCF_017301795.1**	Xanthomonas translucens pv. undulosa	partial	full	full
**GCF_017301815.1**	Xanthomonas translucens pv. undulosa	partial	full	full
**GCF_017301835.1**	Xanthomonas translucens pv. undulosa	partial	full	full
**GCF_001707195.1**	Xanthomonas translucens pv. undulosa	partial	full	full
**GCF_014356755.1**	Xanthomonas translucens pv. undulosa	partial	full	full
**GCF_001707225.1**	Xanthomonas translucens pv. undulosa	partial	full	full
**GCF_020525465.1**	Xanthomonas citri pv. mangiferaeindicae	full	None	None
**GCF_020544545.1**	Xanthomonas citri pv. mangiferaeindicae	full	None	None
**GCF_002920975.3**	Xanthomonas citri pv. mangiferaeindicae	full	None	None
**GCF_002926255.2**	Xanthomonas citri pv. mangiferaeindicae	full	None	None
**GCF_008764405.1**	Xanthomonas citri pv. mangiferaeindicae	None	None	None
**GCF_020879715.1**	Xanthomonas perforans	full	full	None
**GCF_001908855.1**	Xanthomonas perforans	full	full	None
**GCF_013112235.1**	Xanthomonas perforans	full	full	None

**GCF_001009445.1**	Xanthomonas perforans	full	full	None
**GCF_001009365.1**	Xanthomonas perforans	full	full	None
**GCF_001009385.1**	Xanthomonas perforans	full	full	None
**GCF_001009395.1**	Xanthomonas perforans	full	full	None
**GCF_001009405.1**	Xanthomonas perforans	full	full	None
**GCF_001009465.1**	Xanthomonas perforans	full	full	None
**GCF_001009475.1**	Xanthomonas perforans	full	full	None
**GCF_001009485.1**	Xanthomonas perforans	full	full	None
**GCF_001009545.1**	Xanthomonas perforans	full	full	None
**GCF_001009665.1**	Xanthomonas perforans	partial	full	None
**GCF_001009675.1**	Xanthomonas perforans	full	full	None
**GCF_001009685.1**	Xanthomonas perforans	full	full	None
**GCF_001009705.1**	Xanthomonas perforans	full	full	None
**GCF_001009745.1**	Xanthomonas perforans	full	full	None
**GCF_001009765.1**	Xanthomonas perforans	full	full	None
**GCF_001009795.1**	Xanthomonas perforans	full	full	None
**GCF_001009825.1**	Xanthomonas perforans	full	full	None
**GCF_001009845.1**	Xanthomonas perforans	full	full	None
**GCF_001009855.1**	Xanthomonas perforans	full	full	None
**GCF_001009865.1**	Xanthomonas perforans	full	full	None
**GCF_001009885.1**	Xanthomonas perforans	full	full	None
**GCF_001009925.1**	Xanthomonas perforans	full	full	None
**GCF_001009935.1**	Xanthomonas perforans	full	full	None
**GCF_001009945.1**	Xanthomonas perforans	full	full	None
**GCF_001009955.1**	Xanthomonas perforans	full	full	None
**GCF_001010005.1**	Xanthomonas perforans	full	full	None
**GCF_001010015.1**	Xanthomonas perforans	full	full	None
**GCF_001010025.1**	Xanthomonas perforans	full	full	None
**GCF_001010035.1**	Xanthomonas perforans	full	full	None
**GCF_001010085.1**	Xanthomonas perforans	full	full	None
**GCF_001010105.1**	Xanthomonas perforans	full	full	None
**GCF_001976075.1**	Xanthomonas perforans	full	full	None
**GCF_003136155.1**	Xanthomonas perforans	full	full	None
**GCF_003992975.1**	Xanthomonas perforans	full	full	None
**GCF_003993015.1**	Xanthomonas perforans	full	full	None
**GCF_003993025.1**	Xanthomonas perforans	full	full	None
**GCF_003993035.1**	Xanthomonas perforans	full	full	None
**GCF_003993055.1**	Xanthomonas perforans	full	full	None
**GCF_003993095.1**	Xanthomonas perforans	full	full	None
**GCF_003993105.1**	Xanthomonas perforans	full	full	None
**GCF_003993115.1**	Xanthomonas perforans	full	full	None
**GCF_003993135.1**	Xanthomonas perforans	full	full	None

GCF_003993535.1	Xanthomonas perforans	full	full	None
GCF_003993575.1	Xanthomonas perforans	full	full	None
GCF_004102205.1	Xanthomonas perforans	full	full	None
GCF_006979525.1	Xanthomonas perforans	full	full	None
GCF_006979565.1	Xanthomonas perforans	full	full	None
GCF_006979735.1	Xanthomonas perforans	full	full	None
GCF_006979785.1	Xanthomonas perforans	full	full	None
GCF_006979805.1	Xanthomonas perforans	full	full	None
GCF_006979915.1	Xanthomonas perforans	full	full	None
GCF_006980395.1	Xanthomonas perforans	full	full	None
GCF_006980465.1	Xanthomonas perforans	full	full	None
GCF_006980475.1	Xanthomonas perforans	full	full	None
GCF_007713955.1	Xanthomonas perforans	full	full	None
GCF_007713965.1	Xanthomonas perforans	full	full	None
GCF_007713985.1	Xanthomonas perforans	full	full	None
GCF_007714045.1	Xanthomonas perforans	full	full	None
GCF_007714065.1	Xanthomonas perforans	full	full	None
GCF_007714075.1	Xanthomonas perforans	full	full	None
GCF_007714105.1	Xanthomonas perforans	full	full	None
GCF_007714115.1	Xanthomonas perforans	full	full	None
GCF_009733625.1	Xanthomonas perforans	full	full	None
GCF_009733635.1	Xanthomonas perforans	full	full	None
GCF_020879295.1	Xanthomonas perforans	full	full	None
GCF_020879555.1	Xanthomonas perforans	full	full	None
GCF_020879675.1	Xanthomonas perforans	full	full	None
GCF_020879695.1	Xanthomonas perforans	full	full	None
GCF_020879735.1	Xanthomonas perforans	full	full	None
GCF_020879955.1	Xanthomonas perforans	full	full	None
GCF_020880155.1	Xanthomonas perforans	full	full	None
GCF_020880335.1	Xanthomonas perforans	full	full	None
GCF_020881595.1	Xanthomonas perforans	full	full	None
GCF_004102065.1	Xanthomonas perforans	full	full	None
GCF_004102075.1	Xanthomonas perforans	partial	full	None
GCF_004102085.1	Xanthomonas perforans	partial	full	None
GCF_004102095.1	Xanthomonas perforans	full	full	None
GCF_004102165.1	Xanthomonas perforans	full	full	None
GCF_004102175.1	Xanthomonas perforans	full	full	None
GCF_004102215.1	Xanthomonas perforans	partial	full	None
GCF_004102225.1	Xanthomonas perforans	full	full	None
GCF_004102235.1	Xanthomonas perforans	full	full	None
GCF_004102275.1	Xanthomonas perforans	full	full	None

**GCF_004102305.1**	Xanthomonas perforans	full	full	None
**GCF_004102315.1**	Xanthomonas perforans	full	full	None
**GCF_004102325.1**	Xanthomonas perforans	full	full	None
**GCF_004102335.1**	Xanthomonas perforans	full	full	None
**GCF_004102345.1**	Xanthomonas perforans	full	full	None
**GCF_004102405.1**	Xanthomonas perforans	partial	full	None
**GCF_004102415.1**	Xanthomonas perforans	full	full	None
**GCF_004102425.1**	Xanthomonas perforans	full	partial	None
**GCF_004102435.1**	Xanthomonas perforans	partial	full	None
**GCF_004102455.1**	Xanthomonas perforans	full	full	None
**GCF_004102505.1**	Xanthomonas perforans	full	partial	None
**GCF_004102515.1**	Xanthomonas perforans	full	full	None
**GCF_004102525.1**	Xanthomonas perforans	full	full	None
**GCF_004102535.1**	Xanthomonas perforans	full	full	None
**GCF_006979535.1**	Xanthomonas perforans	full	full	None
**GCF_006979545.1**	Xanthomonas perforans	full	full	None
**GCF_006979555.1**	Xanthomonas perforans	full	full	None
**GCF_006979625.1**	Xanthomonas perforans	full	full	None
**GCF_006979635.1**	Xanthomonas perforans	full	full	None
**GCF_006979645.1**	Xanthomonas perforans	full	full	None
**GCF_006979655.1**	Xanthomonas perforans	full	partial	None
**GCF_006979675.1**	Xanthomonas perforans	full	full	None
**GCF_006979715.1**	Xanthomonas perforans	full	full	None
**GCF_006979725.1**	Xanthomonas perforans	full	full	None
**GCF_006979815.1**	Xanthomonas perforans	full	full	None
**GCF_006979835.1**	Xanthomonas perforans	full	full	None
**GCF_006979855.1**	Xanthomonas perforans	full	full	None
**GCF_006979875.1**	Xanthomonas perforans	partial	full	None
**GCF_006979895.1**	Xanthomonas perforans	full	full	None
**GCF_006979945.1**	Xanthomonas perforans	full	full	None
**GCF_006979955.1**	Xanthomonas perforans	full	full	None
**GCF_006979965.1**	Xanthomonas perforans	full	full	None
**GCF_006979975.1**	Xanthomonas perforans	full	full	None
**GCF_006979985.1**	Xanthomonas perforans	full	full	None
**GCF_006980045.1**	Xanthomonas perforans	partial	full	None
**GCF_006980055.1**	Xanthomonas perforans	full	full	None
**GCF_006980075.1**	Xanthomonas perforans	full	full	None
**GCF_006980085.1**	Xanthomonas perforans	full	full	None
**GCF_006980095.1**	Xanthomonas perforans	full	full	None
**GCF_006980115.1**	Xanthomonas perforans	full	full	None
**GCF_006980165.1**	Xanthomonas perforans	partial	full	None
**GCF_006980175.1**	Xanthomonas perforans	full	full	None

**GCF_006980195.1**	Xanthomonas perforans	full	full	None
**GCF_006980215.1**	Xanthomonas perforans	full	full	None
**GCF_006980225.1**	Xanthomonas perforans	full	full	None
**GCF_006980245.1**	Xanthomonas perforans	full	full	None
**GCF_006980275.1**	Xanthomonas perforans	full	full	None
**GCF_006980285.1**	Xanthomonas perforans	full	full	None
**GCF_006980305.1**	Xanthomonas perforans	full	full	None
**GCF_006980315.1**	Xanthomonas perforans	full	full	None
**GCF_006980365.1**	Xanthomonas perforans	full	full	None
**GCF_006980375.1**	Xanthomonas perforans	full	full	None
**GCF_006980385.1**	Xanthomonas perforans	full	full	None
**GCF_006980405.1**	Xanthomonas perforans	full	full	None
**GCF_006980495.1**	Xanthomonas perforans	full	full	None
**GCF_006980505.1**	Xanthomonas perforans	full	full	None
**GCF_006980525.1**	Xanthomonas perforans	full	full	None
**GCF_006980565.1**	Xanthomonas perforans	full	full	None
**GCF_006980575.1**	Xanthomonas perforans	full	full	None
**GCF_006980585.1**	Xanthomonas perforans	full	full	None
**GCF_006980615.1**	Xanthomonas perforans	full	full	None
**GCF_006980625.1**	Xanthomonas perforans	full	full	None
**GCF_006980655.1**	Xanthomonas perforans	full	full	None
**GCF_006980665.1**	Xanthomonas perforans	full	full	None
**GCF_900092025.1**	Xanthomonas bromi	None	None	partial
**GCF_002939755.1**	Xanthomonas bromi	None	None	full
**GCF_900183975.1**	Xanthomonas fragariae	None	None	full
**GCF_001705545.1**	Xanthomonas fragariae	None	None	full
**GCF_001705565.1**	Xanthomonas fragariae	None	None	full
**GCF_017603965.1**	Xanthomonas fragariae	None	None	None
**GCF_900183985.1**	Xanthomonas fragariae	None	None	full
**GCF_900183995.1**	Xanthomonas fragariae	None	None	full
**GCF_016792185.1**	Xanthomonas fragariae	None	None	full
**GCF_016792245.1**	Xanthomonas fragariae	None	None	full
**GCF_900379705.1**	Xanthomonas fragariae	None	None	full
**GCF_900379715.1**	Xanthomonas fragariae	None	None	full
**GCF_900379735.1**	Xanthomonas fragariae	None	None	full
**GCF_900379745.1**	Xanthomonas fragariae	None	None	full
**GCF_900379795.1**	Xanthomonas fragariae	None	None	full
**GCF_900379805.1**	Xanthomonas fragariae	None	None	full
**GCF_900379815.1**	Xanthomonas fragariae	None	None	full
**GCF_900380005.1**	Xanthomonas fragariae	None	None	full
**GCF_900380025.1**	Xanthomonas fragariae	None	None	full

**GCF_900380035.1**	Xanthomonas fragariae	None	None	full
**GCF_900380055.1**	Xanthomonas fragariae	None	None	full
**GCF_900380115.1**	Xanthomonas fragariae	None	None	full
**GCF_900380125.1**	Xanthomonas fragariae	None	None	full
**GCF_900380135.1**	Xanthomonas fragariae	None	None	full
**GCF_900380145.1**	Xanthomonas fragariae	None	None	full
**GCF_900380155.1**	Xanthomonas fragariae	None	None	full
**GCF_900380165.1**	Xanthomonas fragariae	None	None	full
**GCF_900380175.1**	Xanthomonas fragariae	None	None	full
**GCF_900380185.1**	Xanthomonas fragariae	None	None	full
**GCF_900380205.1**	Xanthomonas fragariae	None	None	full
**GCF_900380225.1**	Xanthomonas fragariae	None	None	full
**GCF_900379725.1**	Xanthomonas fragariae	None	None	full
**GCF_900379755.1**	Xanthomonas fragariae	None	None	full
**GCF_900379765.1**	Xanthomonas fragariae	None	None	full
**GCF_900379775.1**	Xanthomonas fragariae	None	None	full
**GCF_900379785.1**	Xanthomonas fragariae	None	None	full
**GCF_900379825.1**	Xanthomonas fragariae	None	None	full
**GCF_900379835.1**	Xanthomonas fragariae	None	None	full
**GCF_900379845.1**	Xanthomonas fragariae	None	None	full
**GCF_900379855.1**	Xanthomonas fragariae	None	None	full
**GCF_900379865.1**	Xanthomonas fragariae	None	None	full
**GCF_900379875.1**	Xanthomonas fragariae	None	None	full
**GCF_900379885.1**	Xanthomonas fragariae	None	None	full
**GCF_900379895.1**	Xanthomonas fragariae	None	None	full
**GCF_900379905.1**	Xanthomonas fragariae	None	None	full
**GCF_900379915.1**	Xanthomonas fragariae	None	None	full
**GCF_900379925.1**	Xanthomonas fragariae	None	None	full
**GCF_900379935.1**	Xanthomonas fragariae	None	None	full
**GCF_900379945.1**	Xanthomonas fragariae	None	None	full
**GCF_900379955.1**	Xanthomonas fragariae	None	None	full
**GCF_900379965.1**	Xanthomonas fragariae	None	None	full
**GCF_900379975.1**	Xanthomonas fragariae	None	None	full
**GCF_900379985.1**	Xanthomonas fragariae	None	None	full
**GCF_900379995.1**	Xanthomonas fragariae	None	None	full
**GCF_900380015.1**	Xanthomonas fragariae	None	None	full
**GCF_900380045.1**	Xanthomonas fragariae	None	None	full
**GCF_900380065.1**	Xanthomonas fragariae	None	None	full
**GCF_900380085.1**	Xanthomonas fragariae	None	None	full
**GCF_900380095.1**	Xanthomonas fragariae	None	None	full
**GCF_900380105.1**	Xanthomonas fragariae	None	None	full
**GCF_900380195.1**	Xanthomonas fragariae	None	None	full

**GCF_900380215.1**	Xanthomonas fragariae	None	None	full
**GCF_900380235.1**	Xanthomonas fragariae	None	None	full
**GCF_900380245.1**	Xanthomonas fragariae	None	None	full
**GCF_903989455.1**	Xanthomonas euroxanthea	None	None	None
**GCF_900476395.1**	Xanthomonas euroxanthea	None	None	None
**GCF_903970585.2**	Xanthomonas euroxanthea	None	None	None
**GCF_905367725.1**	Xanthomonas euroxanthea	None	None	None
**GCF_905367735.1**	Xanthomonas euroxanthea	None	None	None
**GCF_905187425.1**	Xanthomonas euroxanthea	None	None	None
**GCF_905142465.1**	Xanthomonas hydrangea	None	None	None
**GCF_905142485.1**	Xanthomonas hydrangea	None	None	None
**GCF_905142495.1**	Xanthomonas hydrangea	None	None	None
**GCF_000007165.1**	Xanthomonas citri pv. citri str. 306	full	None	None
**GCF_000349225.1**	Xanthomonas citri subsp. citri Aw12879	full	None	None
**GCF_000816885.1**	Xanthomonas citri subsp. citri A306	full	None	None
**GCF_000961175.1**	Xanthomonas citri subsp. citri UI6	full	None	None
**GCF_001610795.1**	Xanthomonas citri pv. aurantifolii	full	None	None
**GCF_001610815.1**	Xanthomonas citri pv. aurantifolii	full	None	None
**GCF_001610915.1**	Xanthomonas citri pv. aurantifolii	full	None	None
**GCF_002079965.1**	Xanthomonas citri pv. aurantifolii	full	None	None
**GCF_004329265.1**	Xanthomonas citri pv. aurantifolii	full	None	None
**GCF_004329275.1**	Xanthomonas citri pv. aurantifolii	full	None	None
**GCF_001854145.2**	Xanthomonas citri pv. glycines str. 8ra	full	None	full
**GCF_000559125.1**	Xanthomonas citri pv. glycines str. 8ra	full	None	full
**GCF_002163775.1**	Xanthomonas citri pv. glycines str. 12-2	full	None	None
**GCF_000259445.1**	Xanthomonas citri pv. glycines str. 12-2	full	None	None
**GCF_002759175.2**	Xanthomonas citri pv. phaseoli var. fuscans	full	None	None
**GCF_002759195.2**	Xanthomonas citri pv. phaseoli var. fuscans	full	None	None
**GCF_002759215.2**	Xanthomonas citri pv. phaseoli var. fuscans	full	partial	None
**GCF_002759235.2**	Xanthomonas citri pv. phaseoli var. fuscans	full	partial	None
**GCF_002759255.2**	Xanthomonas citri pv. phaseoli var. fuscans	full	partial	None
**GCF_002759275.2**	Xanthomonas citri pv. phaseoli var. fuscans	full	partial	None
**GCF_002759295.2**	Xanthomonas citri pv. phaseoli var. fuscans	full	partial	None
**GCF_002759315.2**	Xanthomonas citri pv. phaseoli var. fuscans	full	partial	None
**GCF_002759335.2**	Xanthomonas citri pv. phaseoli var. fuscans	full	None	full
**GCF_002759355.2**	Xanthomonas citri pv. phaseoli var. fuscans	full	partial	None
**GCF_002759375.1**	Xanthomonas citri pv. phaseoli var. fuscans	full	partial	full
**GCF_002759395.2**	Xanthomonas citri pv. phaseoli var. fuscans	full	partial	None
**GCF_002759415.2**	Xanthomonas citri pv. phaseoli var. fuscans	full	partial	None
**GCF_017723855.1**	Xanthomonas citri pv. glycines CFBP 2526	full	None	None
**GCF_000495275.1**	Xanthomonas citri pv. glycines CFBP 2526	full	None	None

**GCF_000009165.1**	Xanthomonas campestris pv. vesicatoria str. 85-10	full	full	None
**GCF_001854165.1**	Xanthomonas campestris pv. vesicatoria str. 85-10	full	full	None
**GCF_000010025.1**	Xanthomonas oryzae pv. oryzae MAFF 311018	partial	full	full
**GCF_000012105.1**	Xanthomonas campestris pv. campestris str. 8004	partial	None	None
**GCF_000019585.2**	Xanthomonas oryzae pv. oryzae PXO99A	partial	full	full
**GCF_000070605.1**	Xanthomonas campestris pv. campestris	partial	None	None
**GCF_001186415.1**	Xanthomonas campestris pv. campestris	partial	None	None
**GCF_001186465.1**	Xanthomonas campestris pv. campestris	partial	None	None
**GCF_002879955.1**	Xanthomonas campestris pv. campestris	partial	None	None
**GCF_009177345.1**	Xanthomonas campestris pv. campestris	partial	None	None
**GCF_000589875.1**	Xanthomonas campestris pv. campestris	partial	None	None
**GCF_001293445.1**	Xanthomonas campestris pv. campestris	partial	None	None
**GCF_006088895.1**	Xanthomonas campestris pv. campestris	partial	None	None
**GCF_006088915.1**	Xanthomonas campestris pv. campestris	partial	None	None
**GCF_019170565.1**	Xanthomonas campestris pv. campestris	partial	None	None
**GCF_001372255.1**	Xanthomonas campestris pv. campestris	partial	None	None
**GCF_002806765.1**	Xanthomonas campestris pv. campestris	partial	None	None
**GCF_003410095.1**	Xanthomonas campestris pv. campestris	partial	None	None
**GCF_003410125.1**	Xanthomonas campestris pv. campestris	partial	None	None
**GCF_003413645.1**	Xanthomonas campestris pv. campestris	partial	None	None
**GCF_013390465.1**	Xanthomonas campestris pv. campestris	partial	None	None
**GCF_020990875.1**	Xanthomonas campestris pv. campestris	partial	None	None
**GCF_020990925.1**	Xanthomonas campestris pv. campestris	partial	None	None
**GCF_020990945.1**	Xanthomonas campestris pv. campestris	partial	None	None
**GCF_020990965.1**	Xanthomonas campestris pv. campestris	partial	None	None
**GCF_020990985.1**	Xanthomonas campestris pv. campestris	partial	None	None
**GCF_000087965.2**	Xanthomonas albilineans GPE PC73	None	None	None
**GCF_000168315.3**	Xanthomonas oryzae pv. oryzicola BLS256	partial	full	full
**GCF_000192045.2**	Xanthomonas perforans 91-118	full	full	None
**GCF_000212755.2**	Xanthomonas oryzae	partial	full	full
**GCF_001276975.2**	Xanthomonas oryzae	partial	full	full
**GCF_004319445.1**	Xanthomonas oryzae	partial	full	full
**GCF_004319465.1**	Xanthomonas oryzae	partial	full	full
**GCF_004319485.1**	Xanthomonas oryzae	partial	full	full
**GCF_004319505.1**	Xanthomonas oryzae	partial	full	full
**GCF_018310515.1**	Xanthomonas oryzae	partial	full	full
**GCF_020866865.1**	Xanthomonas oryzae	partial	full	full
**GCF_001277045.1**	Xanthomonas oryzae	partial	full	full
**GCF_001277055.1**	Xanthomonas oryzae	partial	full	full
**GCF_001277095.1**	Xanthomonas oryzae	partial	full	full
**GCF_000221965.1**	Xanthomonas campestris pv. raphani 756C	partial	None	None
**GCF_000225915.1**	Xanthomonas axonopodis pv. citrumelo F1	full	full	None

**GCF_000348585.1**	Xanthomonas axonopodis Xac29-1	full	None	None
**GCF_000401735.2**	Xanthomonas campestris pv. campestris str. CN14	partial	None	None
**GCF_000403575.2**	Xanthomonas campestris pv. campestris str. CN15	partial	None	None
**GCF_000772715.2**	Xanthomonas vasicola	partial	full	None
**GCF_000772775.3**	Xanthomonas vasicola	partial	full	None
**GCF_000772705.2**	Xanthomonas vasicola	partial	full	None
**GCF_002939925.1**	Xanthomonas vasicola	partial	full	None
**GCF_000772695.1**	Xanthomonas vasicola	partial	full	None
**GCF_000772725.1**	Xanthomonas vasicola	partial	full	None
**GCF_000772785.1**	Xanthomonas vasicola	partial	full	None
**GCF_000772795.1**	Xanthomonas vasicola	partial	full	None
**GCF_003312605.3**	Xanthomonas vasicola	partial	full	None
**GCF_003312615.2**	Xanthomonas vasicola	partial	full	None
**GCF_003312625.2**	Xanthomonas vasicola	partial	full	None
**GCF_003312635.3**	Xanthomonas vasicola	partial	full	None
**GCF_003312685.2**	Xanthomonas vasicola	partial	full	None
**GCF_003312695.2**	Xanthomonas vasicola	partial	full	None
**GCF_003312705.2**	Xanthomonas vasicola	partial	full	None
**GCF_003312715.2**	Xanthomonas vasicola	partial	full	None
**GCF_003312765.2**	Xanthomonas vasicola	partial	full	None
**GCF_003312775.3**	Xanthomonas vasicola	partial	full	None
**GCF_003312785.2**	Xanthomonas vasicola	partial	full	None
**GCF_003312795.4**	Xanthomonas vasicola	partial	full	None
**GCF_003312845.2**	Xanthomonas vasicola	partial	full	None
**GCF_007846135.1**	Xanthomonas vasicola	partial	full	None
**GCF_007846145.1**	Xanthomonas vasicola	partial	full	None
**GCF_007846155.1**	Xanthomonas vasicola	partial	full	None
**GCF_007846165.1**	Xanthomonas vasicola	partial	full	None
**GCF_007846185.1**	Xanthomonas vasicola	partial	full	None
**GCF_007846235.1**	Xanthomonas vasicola	partial	full	None
**GCF_007846245.1**	Xanthomonas vasicola	partial	full	None
**GCF_007846255.1**	Xanthomonas vasicola	partial	full	None
**GCF_007846285.1**	Xanthomonas vasicola	partial	full	None
**GCF_007846295.1**	Xanthomonas vasicola	partial	full	None
**GCF_007846335.1**	Xanthomonas vasicola	partial	full	None
**GCF_007846345.1**	Xanthomonas vasicola	partial	full	None
**GCF_007846385.1**	Xanthomonas vasicola	partial	full	None
**GCF_007846395.1**	Xanthomonas vasicola	partial	full	None
**GCF_007846405.1**	Xanthomonas vasicola	partial	full	None
**GCF_007846415.1**	Xanthomonas vasicola	partial	full	None
**GCF_007846465.1**	Xanthomonas vasicola	partial	full	None

**GCF_007846515.1**	Xanthomonas vasicola	partial	full	None
**GCF_000948075.1**	Xanthomonas oryzae pv. oryzae PXO86	partial	full	full
**GCF_000972745.1**	Xanthomonas arboricola	None	None	None
**GCF_007724205.1**	Xanthomonas arboricola	None	None	None
**GCF_905220695.1**	Xanthomonas arboricola	None	None	None
**GCF_905220745.1**	Xanthomonas arboricola	None	None	None
**GCF_001013485.1**	Xanthomonas arboricola	None	None	None
**GCF_001013505.1**	Xanthomonas arboricola	None	None	None
**GCF_002939805.1**	Xanthomonas arboricola	None	None	None
**GCF_002939825.1**	Xanthomonas arboricola	None	None	None
**GCF_002939965.1**	Xanthomonas arboricola	None	None	None
**GCF_002940475.1**	Xanthomonas arboricola	None	None	None
**GCF_002940505.1**	Xanthomonas arboricola	None	None	None
**GCF_002940665.1**	Xanthomonas arboricola	None	None	None
**GCF_003352905.1**	Xanthomonas arboricola	None	None	None
**GCF_003353025.1**	Xanthomonas arboricola	None	None	None
**GCF_003363845.1**	Xanthomonas arboricola	full	None	None
**GCF_003363915.1**	Xanthomonas arboricola	full	None	None
**GCF_003363925.1**	Xanthomonas arboricola	full	None	None
**GCF_003363955.1**	Xanthomonas arboricola	full	None	None
**GCF_003364015.1**	Xanthomonas arboricola	None	None	None
**GCF_003364025.1**	Xanthomonas arboricola	full	None	None
**GCF_003364075.1**	Xanthomonas arboricola	full	None	None
**GCF_003364095.1**	Xanthomonas arboricola	full	full	None
**GCF_011761585.1**	Xanthomonas arboricola	None	None	None
**GCF_011761665.1**	Xanthomonas arboricola	None	None	None
**GCF_011761675.1**	Xanthomonas arboricola	None	None	None
**GCF_011761705.1**	Xanthomonas arboricola	None	None	None
**GCF_011761715.1**	Xanthomonas arboricola	None	None	None
**GCF_011761865.1**	Xanthomonas arboricola	None	None	None
**GCF_011927445.1**	Xanthomonas arboricola	None	None	None
**GCF_011927585.1**	Xanthomonas arboricola	None	None	None
**GCF_011927595.1**	Xanthomonas arboricola	None	None	None
**GCF_014195715.1**	Xanthomonas arboricola	None	None	None
**GCF_014195725.1**	Xanthomonas arboricola	None	None	None
**GCF_014195775.1**	Xanthomonas arboricola	None	None	None
**GCF_014196045.1**	Xanthomonas arboricola	None	None	None
**GCF_014198985.1**	Xanthomonas arboricola	None	None	None
**GCF_014199015.1**	Xanthomonas arboricola	None	None	None
**GCF_014199025.1**	Xanthomonas arboricola	None	None	None
**GCF_014199055.1**	Xanthomonas arboricola	None	None	None
**GCF_014199075.1**	Xanthomonas arboricola	None	None	None

**GCF_014199095.1**	Xanthomonas arboricola	None	None	None
**GCF_014199105.1**	Xanthomonas arboricola	None	None	None
**GCF_014199735.1**	Xanthomonas arboricola	None	None	None
**GCF_014199755.1**	Xanthomonas arboricola	None	None	None
**GCF_014199835.1**	Xanthomonas arboricola	None	None	None
**GCF_014199845.1**	Xanthomonas arboricola	None	None	None
**GCF_014205665.1**	Xanthomonas arboricola	None	None	None
**GCF_014206795.1**	Xanthomonas arboricola	None	None	None
**GCF_014206935.1**	Xanthomonas arboricola	None	None	None
**GCF_001264265.1**	Xanthomonas arboricola	None	None	None
**GCF_001264275.1**	Xanthomonas arboricola	None	None	None
**GCF_001264285.1**	Xanthomonas arboricola	None	None	None
**GCF_001264295.1**	Xanthomonas arboricola	None	None	None
**GCF_001264345.1**	Xanthomonas arboricola	None	None	None
**GCF_001264355.1**	Xanthomonas arboricola	None	None	None
**GCF_001264385.1**	Xanthomonas arboricola	None	None	None
**GCF_001264425.1**	Xanthomonas arboricola	None	None	None
**GCF_001264435.1**	Xanthomonas arboricola	None	None	None
**GCF_001264465.1**	Xanthomonas arboricola	None	None	None
**GCF_001266515.1**	Xanthomonas arboricola	None	None	None
**GCF_001266525.1**	Xanthomonas arboricola	None	None	None
**GCF_001266535.1**	Xanthomonas arboricola	None	None	None
**GCF_001306965.1**	Xanthomonas arboricola	None	None	None
**GCF_001674995.1**	Xanthomonas arboricola	None	None	None
**GCF_001675005.1**	Xanthomonas arboricola	None	None	None
**GCF_002940095.1**	Xanthomonas arboricola	None	None	None
**GCF_002940165.1**	Xanthomonas arboricola	None	None	None
**GCF_002940225.1**	Xanthomonas arboricola	None	None	None
**GCF_002940265.1**	Xanthomonas arboricola	None	None	None
**GCF_002940285.1**	Xanthomonas arboricola	None	None	None
**GCF_002940305.1**	Xanthomonas arboricola	None	None	None
**GCF_002940315.1**	Xanthomonas arboricola	None	None	None
**GCF_002940345.1**	Xanthomonas arboricola	None	None	None
**GCF_002940365.1**	Xanthomonas arboricola	None	None	None
**GCF_002940385.1**	Xanthomonas arboricola	None	None	None
**GCF_002940405.1**	Xanthomonas arboricola	None	None	None
**GCF_002940425.1**	Xanthomonas arboricola	None	None	None
**GCF_002940445.1**	Xanthomonas arboricola	None	None	None
**GCF_002940465.1**	Xanthomonas arboricola	None	None	None
**GCF_002940525.1**	Xanthomonas arboricola	None	None	None
**GCF_002940545.1**	Xanthomonas arboricola	None	None	None

**GCF_003353015.1**	Xanthomonas arboricola	None	None	None
**GCF_003993355.1**	Xanthomonas arboricola	None	None	None
**GCF_003993395.1**	Xanthomonas arboricola	None	None	None
**GCF_003993415.1**	Xanthomonas arboricola	None	None	None
**GCF_003993715.1**	Xanthomonas arboricola	None	None	None
**GCF_003993745.1**	Xanthomonas arboricola	None	None	None
**GCF_011761775.1**	Xanthomonas arboricola	None	None	None
**GCF_014195785.1**	Xanthomonas arboricola	None	None	None
**GCF_014195835.1**	Xanthomonas arboricola	None	None	None
**GCF_014199115.1**	Xanthomonas arboricola	None	None	None
**GCF_014199515.1**	Xanthomonas arboricola	None	None	None
**GCF_014199765.1**	Xanthomonas arboricola	None	None	None
**GCF_020879215.1**	Xanthomonas arboricola	None	None	None
**GCF_020880475.1**	Xanthomonas arboricola	None	None	None
**GCF_001908755.1**	Xanthomonas hortorum pv. gardneri	None	None	None
**GCF_001908775.1**	Xanthomonas hortorum pv. gardneri	None	None	None
**GCF_903978225.1**	Xanthomonas hortorum pv. gardneri	None	None	None
**GCF_001009285.1**	Xanthomonas hortorum pv. gardneri	None	None	None
**GCF_001009295.1**	Xanthomonas hortorum pv. gardneri	None	None	None
**GCF_001009315.1**	Xanthomonas hortorum pv. gardneri	None	None	None
**GCF_001009325.1**	Xanthomonas hortorum pv. gardneri	None	None	None
**GCF_001009525.1**	Xanthomonas hortorum pv. gardneri	None	None	None
**GCF_001009535.1**	Xanthomonas hortorum pv. gardneri	None	None	None
**GCF_001009585.1**	Xanthomonas hortorum pv. gardneri	None	None	None
**GCF_001009605.1**	Xanthomonas hortorum pv. gardneri	None	None	None
**GCF_001009615.1**	Xanthomonas hortorum pv. gardneri	None	None	None
**GCF_001009625.1**	Xanthomonas hortorum pv. gardneri	None	None	None
**GCF_012922265.1**	Xanthomonas hortorum pv. gardneri	None	None	None
**GCF_020879225.1**	Xanthomonas hortorum pv. gardneri	None	None	None
**GCF_020879305.1**	Xanthomonas hortorum pv. gardneri	None	None	None
**GCF_020879315.1**	Xanthomonas hortorum pv. gardneri	None	None	None
**GCF_020879365.1**	Xanthomonas hortorum pv. gardneri	None	None	None
**GCF_020879455.1**	Xanthomonas hortorum pv. gardneri	None	None	full
**GCF_020879495.1**	Xanthomonas hortorum pv. gardneri	None	None	None
**GCF_020879535.1**	Xanthomonas hortorum pv. gardneri	None	None	None
**GCF_020879895.1**	Xanthomonas hortorum pv. gardneri	None	None	None
**GCF_020879935.1**	Xanthomonas hortorum pv. gardneri	None	None	None
**GCF_020879965.1**	Xanthomonas hortorum pv. gardneri	None	None	None
**GCF_020880095.1**	Xanthomonas hortorum pv. gardneri	None	None	None
**GCF_020880195.1**	Xanthomonas hortorum pv. gardneri	None	None	None
**GCF_020880245.1**	Xanthomonas hortorum pv. gardneri	None	None	None
**GCF_020880285.1**	Xanthomonas hortorum pv. gardneri	None	None	None

**GCF_020880455.1**	Xanthomonas hortorum pv. gardneri	None	None	None
**GCF_020880795.1**	Xanthomonas hortorum pv. gardneri	None	None	None
**GCF_020880915.1**	Xanthomonas hortorum pv. gardneri	None	None	None
**GCF_020880935.1**	Xanthomonas hortorum pv. gardneri	None	None	None
**GCF_021353075.1**	Xanthomonas hortorum pv. gardneri	None	None	None
**GCF_001908795.1**	Xanthomonas euvesicatoria	full	full	None
**GCF_001401555.1**	Xanthomonas euvesicatoria	partial	partial	None
**GCF_000802325.1**	Xanthomonas euvesicatoria	full	full	None
**GCF_000802345.1**	Xanthomonas euvesicatoria	full	full	None
**GCF_001008805.1**	Xanthomonas euvesicatoria	full	full	None
**GCF_001008815.1**	Xanthomonas euvesicatoria	partial	full	None
**GCF_001008825.1**	Xanthomonas euvesicatoria	full	full	None
**GCF_001008835.1**	Xanthomonas euvesicatoria	full	full	None
**GCF_001008885.1**	Xanthomonas euvesicatoria	full	full	None
**GCF_001008895.1**	Xanthomonas euvesicatoria	full	full	None
**GCF_001008905.1**	Xanthomonas euvesicatoria	full	full	None
**GCF_001008915.1**	Xanthomonas euvesicatoria	full	full	None
**GCF_001008965.1**	Xanthomonas euvesicatoria	partial	full	None
**GCF_001008975.1**	Xanthomonas euvesicatoria	full	full	None
**GCF_001008985.1**	Xanthomonas euvesicatoria	partial	full	None
**GCF_001008995.1**	Xanthomonas euvesicatoria	full	full	None
**GCF_001009045.1**	Xanthomonas euvesicatoria	full	full	None
**GCF_001009055.1**	Xanthomonas euvesicatoria	full	full	None
**GCF_001009075.1**	Xanthomonas euvesicatoria	full	full	None
**GCF_001009095.1**	Xanthomonas euvesicatoria	full	full	None
**GCF_001009125.1**	Xanthomonas euvesicatoria	full	full	None
**GCF_001009135.1**	Xanthomonas euvesicatoria	full	full	None
**GCF_001009165.1**	Xanthomonas euvesicatoria	full	full	None
**GCF_001009175.1**	Xanthomonas euvesicatoria	full	full	None
**GCF_001009205.1**	Xanthomonas euvesicatoria	full	full	None
**GCF_001009215.1**	Xanthomonas euvesicatoria	full	full	None
**GCF_001009245.1**	Xanthomonas euvesicatoria	full	full	None
**GCF_001009255.1**	Xanthomonas euvesicatoria	full	full	None
**GCF_001010095.1**	Xanthomonas euvesicatoria	full	full	None
**GCF_001401675.2**	Xanthomonas euvesicatoria	full	full	None
**GCF_001691315.1**	Xanthomonas euvesicatoria	full	full	None
**GCF_001691325.1**	Xanthomonas euvesicatoria	full	full	None
**GCF_001691345.1**	Xanthomonas euvesicatoria	full	full	None
**GCF_001691375.1**	Xanthomonas euvesicatoria	full	full	None
**GCF_001691385.1**	Xanthomonas euvesicatoria	full	full	None
**GCF_003136175.1**	Xanthomonas euvesicatoria	full	full	None

**GCF_003992785.1**	Xanthomonas euvesicatoria	full	full	None
**GCF_003992805.1**	Xanthomonas euvesicatoria	full	full	None
**GCF_003993175.1**	Xanthomonas euvesicatoria	full	full	None
**GCF_003993185.1**	Xanthomonas euvesicatoria	full	full	None
**GCF_003993195.1**	Xanthomonas euvesicatoria	full	full	None
**GCF_003993225.1**	Xanthomonas euvesicatoria	full	full	None
**GCF_003993255.1**	Xanthomonas euvesicatoria	full	full	None
**GCF_003993265.1**	Xanthomonas euvesicatoria	full	full	None
**GCF_003993275.1**	Xanthomonas euvesicatoria	full	full	None
**GCF_003993315.1**	Xanthomonas euvesicatoria	full	full	None
**GCF_003993335.1**	Xanthomonas euvesicatoria	full	full	None
**GCF_003993345.1**	Xanthomonas euvesicatoria	full	full	None
**GCF_003993445.1**	Xanthomonas euvesicatoria	full	full	None
**GCF_003993595.1**	Xanthomonas euvesicatoria	full	full	None
**GCF_003993605.1**	Xanthomonas euvesicatoria	full	full	None
**GCF_003993615.1**	Xanthomonas euvesicatoria	full	full	None
**GCF_003993655.1**	Xanthomonas euvesicatoria	full	full	None
**GCF_003993675.1**	Xanthomonas euvesicatoria	full	full	None
**GCF_003993685.1**	Xanthomonas euvesicatoria	full	full	None
**GCF_003993725.1**	Xanthomonas euvesicatoria	full	full	None
**GCF_014198935.1**	Xanthomonas euvesicatoria	full	None	None
**GCF_002759095.2**	Xanthomonas phaseoli pv. phaseoli	full	None	None
**GCF_002759115.2**	Xanthomonas phaseoli pv. phaseoli	full	None	None
**GCF_002759135.1**	Xanthomonas phaseoli pv. phaseoli	full	None	None
**GCF_002759155.2**	Xanthomonas phaseoli pv. phaseoli	full	None	None
**GCF_003999445.1**	Xanthomonas phaseoli pv. phaseoli	full	None	None
**GCF_003999545.1**	Xanthomonas phaseoli pv. phaseoli	full	None	None
**GCF_003999565.1**	Xanthomonas phaseoli pv. phaseoli	full	None	None
**GCF_017742795.1**	Xanthomonas phaseoli pv. phaseoli	full	None	None
**GCF_018831405.1**	Xanthomonas phaseoli pv. phaseoli	full	None	None
**GCF_018831425.1**	Xanthomonas phaseoli pv. phaseoli	full	None	None
**GCF_018831445.1**	Xanthomonas phaseoli pv. phaseoli	full	None	None
**GCF_000774035.1**	Xanthomonas phaseoli pv. phaseoli	full	None	None
**GCF_017745345.1**	Xanthomonas phaseoli pv. phaseoli	full	None	None
**GCF_000775205.1**	Xanthomonas phaseoli pv. phaseoli	full	partial	None
**GCF_000785925.1**	Xanthomonas phaseoli pv. phaseoli	full	None	None
**GCF_000785935.1**	Xanthomonas phaseoli pv. phaseoli	full	None	None
**GCF_000785945.1**	Xanthomonas phaseoli pv. phaseoli	full	None	None
**GCF_000786915.1**	Xanthomonas phaseoli pv. phaseoli	full	partial	None
**GCF_000786995.1**	Xanthomonas phaseoli pv. phaseoli	partial	None	None
**GCF_000807875.2**	Xanthomonas phaseoli pv. phaseoli	full	None	None
**GCF_000808655.2**	Xanthomonas phaseoli pv. phaseoli	partial	None	None

**GCF_000808675.2**	Xanthomonas phaseoli pv. phaseoli	full	None	None
**GCF_000808695.2**	Xanthomonas phaseoli pv. phaseoli	full	None	None
**GCF_000808715.2**	Xanthomonas phaseoli pv. phaseoli	full	None	full
**GCF_000808735.2**	Xanthomonas phaseoli pv. phaseoli	full	None	full
**GCF_000818835.2**	Xanthomonas phaseoli pv. phaseoli	partial	None	None
**GCF_000827975.2**	Xanthomonas phaseoli pv. phaseoli	full	None	None
**GCF_017745315.1**	Xanthomonas phaseoli pv. phaseoli	full	None	None
**GCF_900234435.1**	Xanthomonas phaseoli pv. phaseoli	full	None	None
**GCF_900234425.1**	Xanthomonas phaseoli pv. phaseoli	full	None	None
**GCF_900234535.1**	Xanthomonas phaseoli pv. phaseoli	full	None	None
**GCF_900234445.1**	Xanthomonas phaseoli pv. phaseoli	full	None	None
**GCF_900234525.1**	Xanthomonas phaseoli pv. phaseoli	full	partial	None
**GCF_002776715.1**	Xanthomonas campestris pv. campestris str. CN17	partial	None	None
**GCF_002776735.1**	Xanthomonas campestris pv. campestris str. CN03	partial	None	None
**GCF_002776775.1**	Xanthomonas campestris pv. campestris str. CN12	partial	None	None
**GCF_900002235.1**	Xanthomonas campestris pv. campestris str. CN12	partial	None	None
**GCF_002776835.1**	Xanthomonas campestris pv. campestris str. CN18	partial	None	None
**GCF_003698225.1**	Xanthomonas axonopodis pv. commiphoreae	full	partial	None
**GCF_008761555.1**	Xanthomonas arboricola pv. pruni	None	None	None
**GCF_001306955.1**	Xanthomonas arboricola pv. pruni	None	None	None
**GCF_001741965.1**	Xanthomonas arboricola pv. pruni	None	None	None
**GCF_003984685.1**	Xanthomonas arboricola pv. pruni	None	None	None
**GCF_003984725.1**	Xanthomonas arboricola pv. pruni	None	None	None
**GCF_020002335.1**	Xanthomonas arboricola pv. pruni	None	None	None
**GCF_020012115.1**	Xanthomonas arboricola pv. pruni	None	None	None
**GCF_020012175.1**	Xanthomonas arboricola pv. pruni	None	None	None
**GCF_020043425.1**	Xanthomonas arboricola pv. pruni	None	None	None
**GCF_014338485.1**	Xanthomonas hortorum pv. vitians	None	None	None
**GCF_903978195.1**	Xanthomonas hortorum pv. vitians	None	None	None
**GCF_012922125.1**	Xanthomonas hortorum pv. vitians	None	None	None
**GCF_012922135.1**	Xanthomonas hortorum pv. vitians	None	None	None
**GCF_012922175.1**	Xanthomonas hortorum pv. vitians	None	None	None
**GCF_012922195.1**	Xanthomonas hortorum pv. vitians	None	None	None
**GCF_012922255.1**	Xanthomonas hortorum pv. vitians	None	None	None
**GCF_012922335.1**	Xanthomonas hortorum pv. vitians	None	None	None
**GCF_021352675.1**	Xanthomonas hortorum pv. vitians	None	None	None
**GCF_021352685.1**	Xanthomonas hortorum pv. vitians	None	None	None
**GCF_021352695.1**	Xanthomonas hortorum pv. vitians	None	None	None
**GCF_021352715.1**	Xanthomonas hortorum pv. vitians	None	None	None
**GCF_021352775.1**	Xanthomonas hortorum pv. vitians	None	None	None
**GCF_021352845.1**	Xanthomonas hortorum pv. vitians	None	None	None

**GCF_021353115.1**	Xanthomonas hortorum pv. vitians	None	None	None
**GCF_021353135.1**	Xanthomonas hortorum pv. vitians	None	None	None
**GCF_021352705.1**	Xanthomonas hortorum pv. vitians	None	None	None
**GCF_021352795.1**	Xanthomonas hortorum pv. vitians	None	None	None
**GCF_021352805.1**	Xanthomonas hortorum pv. vitians	None	None	None
**GCF_021352835.1**	Xanthomonas hortorum pv. vitians	None	None	None
**GCF_021352875.1**	Xanthomonas hortorum pv. vitians	None	None	None
**GCF_021352895.1**	Xanthomonas hortorum pv. vitians	None	None	None
**GCF_021352915.1**	Xanthomonas hortorum pv. vitians	None	None	None
**GCF_021352935.1**	Xanthomonas hortorum pv. vitians	None	None	None
**GCF_021352975.1**	Xanthomonas hortorum pv. vitians	None	None	None
**GCF_021353035.1**	Xanthomonas hortorum pv. vitians	None	None	None
**GCF_014841015.1**	Xanthomonas campestris	partial	None	None
**GCF_000590375.1**	Xanthomonas campestris	partial	None	None
**GCF_003352895.1**	Xanthomonas campestris	full	full	None
**GCF_003352955.1**	Xanthomonas campestris	full	full	None
**GCF_003352965.1**	Xanthomonas campestris	full	None	None
**GCF_003363795.1**	Xanthomonas campestris	full	None	None
**GCF_003363805.1**	Xanthomonas campestris	full	full	None
**GCF_003363835.1**	Xanthomonas campestris	full	None	None
**GCF_003363965.1**	Xanthomonas campestris	full	None	None
**GCF_003363995.1**	Xanthomonas campestris	full	None	None
**GCF_003364005.1**	Xanthomonas campestris	full	None	None
**GCF_003602375.1**	Xanthomonas campestris	full	None	None
**GCF_011761725.1**	Xanthomonas campestris	None	None	None
**GCF_011761845.1**	Xanthomonas campestris	None	None	None
**GCF_011761885.1**	Xanthomonas campestris	None	None	None
**GCF_014195735.1**	Xanthomonas campestris	None	None	None
**GCF_000834675.1**	Xanthomonas campestris	full	None	None
**GCF_001705825.1**	Xanthomonas campestris	partial	None	None
**GCF_002916725.2**	Xanthomonas campestris	full	partial	None
**GCF_003111945.1**	Xanthomonas campestris	partial	None	None
**GCF_003410175.1**	Xanthomonas campestris	partial	None	None
**GCF_003410215.1**	Xanthomonas campestris	partial	None	None
**GCF_003410225.1**	Xanthomonas campestris	partial	None	None
**GCF_011761915.1**	Xanthomonas campestris	None	None	None
**GCF_014197275.1**	Xanthomonas campestris	None	None	None
**GCF_014839145.1**	Xanthomonas campestris	partial	None	None
**GCF_020178995.1**	Xanthomonas campestris	full	None	None
**GCF_020879405.1**	Xanthomonas campestris	partial	None	None
**GCF_020880515.1**	Xanthomonas campestris	partial	None	None
**GCF_020880535.1**	Xanthomonas campestris	partial	None	None

**GCF_017301655.1**	Xanthomonas translucens pv. translucens	partial	full	full
**GCF_017301675.1**	Xanthomonas translucens pv. translucens	partial	full	full
**GCF_017301695.1**	Xanthomonas translucens pv. translucens	partial	full	None
**GCF_017301715.1**	Xanthomonas translucens pv. translucens	partial	full	None
**GCF_017301855.1**	Xanthomonas translucens pv. translucens	partial	full	None
**GCF_001469515.1**	Xanthomonas translucens pv. translucens	partial	full	full
**GCF_001707125.1**	Xanthomonas translucens pv. translucens	partial	full	full
**GCF_009600865.1**	Xanthomonas translucens pv. translucens	partial	full	full
**GCF_020880735.1**	Xanthomonas translucens pv. translucens	partial	full	None
**GCF_001659915.1**	Xanthomonas translucens pv. translucens	partial	full	full
**GCF_001707275.1**	Xanthomonas translucens pv. translucens	partial	full	full
**GCF_018141705.1**	Xanthomonas arboricola pv. corylina	None	None	None
**GCF_002939845.1**	Xanthomonas arboricola pv. corylina	None	None	None
**GCF_002940125.1**	Xanthomonas arboricola pv. corylina	None	None	None
**GCF_020008105.1**	Xanthomonas campestris pv. incanae	partial	None	None
**GCF_000590315.1**	Xanthomonas campestris pv. incanae	partial	None	None
**GCF_000590335.1**	Xanthomonas campestris pv. incanae	partial	None	None
**GCF_003410195.1**	Xanthomonas campestris pv. incanae	partial	None	None
**GCF_020866885.1**	Xanthomonas phaseoli pv. manihotis	full	None	None
**GCF_001482665.1**	Xanthomonas phaseoli pv. manihotis	full	None	None
**GCF_015352415.1**	Xanthomonas phaseoli pv. manihotis	full	None	None
**GCF_015352865.1**	Xanthomonas phaseoli pv. manihotis	full	None	None
**GCF_017745335.1**	Xanthomonas phaseoli pv. manihotis	full	None	None
**GCF_017745415.1**	Xanthomonas phaseoli pv. manihotis	full	None	None
**GCF_017745455.1**	Xanthomonas phaseoli pv. manihotis	full	None	None
**GCF_017745475.1**	Xanthomonas phaseoli pv. manihotis	full	None	None
**GCF_017745505.1**	Xanthomonas phaseoli pv. manihotis	full	None	None
**GCF_021390095.1**	Xanthomonas translucens	partial	full	full
**GCF_021390115.1**	Xanthomonas translucens	partial	full	full
**GCF_001455815.1**	Xanthomonas translucens	partial	partial	full
**GCF_001455835.1**	Xanthomonas translucens	partial	full	full
**GCF_001455845.1**	Xanthomonas translucens	partial	full	full
**GCF_001461905.1**	Xanthomonas translucens	partial	full	full
**GCF_001461915.1**	Xanthomonas translucens	partial	full	full
**GCF_001461925.1**	Xanthomonas translucens	partial	full	full
**GCF_001461965.1**	Xanthomonas translucens	partial	full	full
**GCF_001461975.1**	Xanthomonas translucens	partial	full	full
**GCF_001461995.1**	Xanthomonas translucens	partial	full	full
**GCF_001462005.1**	Xanthomonas translucens	partial	full	full
**GCF_001462045.1**	Xanthomonas translucens	partial	full	full
**GCF_001462065.1**	Xanthomonas translucens	partial	full	full

**GCF_001462075.1**	Xanthomonas translucens	partial	full	full
**GCF_001462095.1**	Xanthomonas translucens	partial	full	full
**GCF_001462145.1**	Xanthomonas translucens	partial	full	full
**GCF_001462155.1**	Xanthomonas translucens	partial	full	full
**GCF_001542045.1**	Xanthomonas translucens	partial	partial	None
**GCF_001542205.1**	Xanthomonas translucens	partial	full	full
**GCF_900094325.1**	Xanthomonas translucens pv. translucens DSM 18974	partial	full	None
**GCF_000331775.1**	Xanthomonas translucens pv. translucens DSM 18974	partial	partial	None
**GCF_905367745.1**	Xanthomonas arboricola pv. juglandis	None	None	None
**GCF_001013475.1**	Xanthomonas arboricola pv. juglandis	None	None	None
**GCF_001237985.1**	Xanthomonas arboricola pv. juglandis	None	None	None
**GCF_001013495.1**	Xanthomonas arboricola pv. juglandis	None	None	None
**GCF_002879695.1**	Xanthomonas arboricola pv. juglandis	None	None	None
**GCF_900537245.1**	Xanthomonas arboricola pv. juglandis	None	None	None
**GCF_900537265.1**	Xanthomonas arboricola pv. juglandis	None	None	None
**GCF_001643295.1**	Xanthomonas arboricola pv. juglandis	None	None	None
**GCF_002940235.1**	Xanthomonas arboricola pv. juglandis	None	None	None
**GCF_900476315.1**	Xanthomonas arboricola pv. juglandis	None	None	None
**GCF_900537235.1**	Xanthomonas arboricola pv. juglandis	None	None	None
**GCF_903989475.2**	Xanthomonas arboricola pv. juglandis	None	None	None
**GCF_905367715.1**	Xanthomonas arboricola pv. juglandis	None	None	None
**GCF_000192065.1**	Xanthomonas gardneri ATCC 19865	None	None	None
**GCF_000263335.1**	Xanthomonas citri pv. mangiferaeindicae LMG 941	full	None	None
**GCF_000285775.1**	Xanthomonas citri pv. punicae str. LMG 859	full	None	None
**GCF_000403555.2**	Xanthomonas campestris pv. campestris str. CN16	partial	None	None
**GCF_000454505.1**	Xanthomonas citri pv. malvacearum X18	full	None	None
**GCF_000454525.1**	Xanthomonas citri pv. malvacearum X20	full	None	None
**GCF_000482445.1**	Xanthomonas oryzae ATCC 35933	partial	full	full
**GCF_000488895.1**	Xanthomonas citri pv. glycines CFBP 7119	full	None	full
**GCF_000488955.1**	Xanthomonas euvesicatoria pv. alfalfae CFBP 3836	full	full	None
**GCF_000505565.1**	Xanthomonas hortorum pv. carotae str. M081	None	None	None
**GCF_000589895.1**	Xanthomonas campestris pv. campestris str. CFBP 1712	partial	None	None
**GCF_000589915.1**	Xanthomonas campestris pv. campestris str. CFBP 1869	partial	None	None
**GCF_000589935.1**	Xanthomonas campestris pv. campestris str. CFBP 4954	partial	None	None
**GCF_000589955.1**	Xanthomonas campestris pv. campestris str. CFBP 4955	partial	None	None
**GCF_000589975.1**	Xanthomonas campestris pv. campestris str. CFBP 5130	partial	None	None
**GCF_000589995.1**	Xanthomonas campestris pv. campestris str. CFBP 5683	partial	None	None
**GCF_000590075.1**	Xanthomonas campestris pv. campestris str. CFBP 5817	partial	None	None

**GCF_000590115.1**	Xanthomonas campestris pv. campestris str. CN01	partial	None	None
**GCF_000590195.1**	Xanthomonas campestris pv. campestris str. CN10	partial	None	None
**GCF_000590275.1**	Xanthomonas campestris pv. campestris str. CFBP 1124	partial	None	None
**GCF_000590295.1**	Xanthomonas campestris pv. campestris str. 147	partial	None	None
**GCF_000723725.1**	Xanthomonas citri pv. viticola	full	None	None
**GCF_002564485.1**	Xanthomonas citri pv. viticola	full	None	None
**GCF_000730305.1**	Xanthomonas euvesicatoria pv. allii CFBP 6369	full	full	None
**GCF_000807145.1**	Xanthomonas translucens pv. cerealis	partial	full	full
**GCF_006838285.1**	Xanthomonas translucens pv. cerealis	partial	full	full
**GCF_001707115.1**	Xanthomonas translucens pv. cerealis	partial	full	full
**GCF_001010415.1**	Xanthomonas pisi DSM 18956	partial	partial	None
**GCF_001304695.1**	Xanthomonas axonopodis	partial	full	None
**GCF_014198975.1**	Xanthomonas axonopodis	full	None	None
**GCF_003111925.1**	Xanthomonas axonopodis	partial	full	None
**GCF_001401595.1**	Xanthomonas axonopodis pv. axonopodis	partial	full	None
**GCF_001908815.1**	Xanthomonas vesicatoria	full	None	None
**GCF_000803145.1**	Xanthomonas vesicatoria	full	None	None
**GCF_000803155.1**	Xanthomonas vesicatoria	full	None	None
**GCF_001469445.1**	Xanthomonas vesicatoria	full	None	None
**GCF_001469465.1**	Xanthomonas vesicatoria	full	None	None
**GCF_003992775.1**	Xanthomonas vesicatoria	full	None	None
**GCF_003992855.1**	Xanthomonas vesicatoria	full	None	None
**GCF_003992865.1**	Xanthomonas vesicatoria	full	None	None
**GCF_003992875.1**	Xanthomonas vesicatoria	full	None	None
**GCF_003992905.1**	Xanthomonas vesicatoria	full	None	None
**GCF_003992935.1**	Xanthomonas vesicatoria	full	None	None
**GCF_003992955.1**	Xanthomonas vesicatoria	full	None	None
**GCF_003993455.1**	Xanthomonas vesicatoria	full	None	None
**GCF_020879145.1**	Xanthomonas vesicatoria	full	None	None
**GCF_020879165.1**	Xanthomonas vesicatoria	full	None	None
**GCF_020879355.1**	Xanthomonas vesicatoria	full	None	None
**GCF_020879575.1**	Xanthomonas vesicatoria	full	None	None
**GCF_020879595.1**	Xanthomonas vesicatoria	full	None	None
**GCF_020879915.1**	Xanthomonas vesicatoria	full	None	None
**GCF_020879975.1**	Xanthomonas vesicatoria	full	None	None
**GCF_020880075.1**	Xanthomonas vesicatoria	full	None	None
**GCF_020880495.1**	Xanthomonas vesicatoria	full	None	None
**GCF_020880545.1**	Xanthomonas vesicatoria	full	None	None
**GCF_020880595.1**	Xanthomonas vesicatoria	full	None	None
**GCF_020880615.1**	Xanthomonas vesicatoria	full	None	None

**GCF_020880745.1**	Xanthomonas vesicatoria	full	None	None
**GCF_020880875.1**	Xanthomonas vesicatoria	full	None	None
**GCF_020880925.1**	Xanthomonas vesicatoria	full	None	None
**GCF_002688625.1**	Xanthomonas citri pv. anacardii CFBP 2913	full	partial	None
**GCF_002906675.1**	Xanthomonas citri pv. fuscans CFBP 6988	full	partial	None
**GCF_002939985.1**	Xanthomonas hortorum pv. cynarae	None	None	None
**GCF_021352995.1**	Xanthomonas hortorum pv. cynarae	None	None	None
**GCF_003064105.1**	Xanthomonas hortorum pv. hederae	None	None	full
**GCF_002940005.1**	Xanthomonas hortorum pv. hederae	None	None	full
**GCF_021353015.1**	Xanthomonas hortorum pv. hederae	None	None	full
**GCF_003999525.1**	Xanthomonas sp. ISO98C4	full	None	None
**GCF_014236815.1**	Xanthomonas sp. GW	full	None	None
**GCF_014236835.1**	Xanthomonas sp. SS	full	None	None
**GCF_014236855.1**	Xanthomonas sp. SI	full	None	None
**GCF_015224665.2**	Xanthomonas sp. WG16	full	None	None
**GCF_017746675.1**	Xanthomonas phaseoli pv. dieffenbachiae	full	full	None
**GCF_001401535.2**	Xanthomonas phaseoli pv. dieffenbachiae	full	full	None
**GCF_017745535.1**	Xanthomonas phaseoli pv. dieffenbachiae	full	full	None
**GCF_017745555.1**	Xanthomonas phaseoli pv. dieffenbachiae	full	full	None
**GCF_017745565.1**	Xanthomonas phaseoli pv. dieffenbachiae	full	full	None
**GCF_017745615.1**	Xanthomonas phaseoli pv. dieffenbachiae	full	full	None
**GCF_017745685.1**	Xanthomonas phaseoli pv. dieffenbachiae	full	full	None
**GCF_017745715.1**	Xanthomonas phaseoli pv. dieffenbachiae	full	full	None
**GCF_017745725.1**	Xanthomonas phaseoli pv. dieffenbachiae	full	full	None
**GCF_017745755.1**	Xanthomonas phaseoli pv. dieffenbachiae	full	full	None
**GCF_017745775.1**	Xanthomonas phaseoli pv. dieffenbachiae	full	full	None
**GCF_017745795.1**	Xanthomonas phaseoli pv. dieffenbachiae	full	full	None
**GCF_017745815.1**	Xanthomonas phaseoli pv. dieffenbachiae	full	full	None
**GCF_017745835.1**	Xanthomonas phaseoli pv. dieffenbachiae	full	full	None
**GCF_017745855.1**	Xanthomonas phaseoli pv. dieffenbachiae	full	full	None
**GCF_017745895.1**	Xanthomonas phaseoli pv. dieffenbachiae	full	full	None
**GCF_017745915.1**	Xanthomonas phaseoli pv. dieffenbachiae	full	full	None
**GCF_017745935.1**	Xanthomonas phaseoli pv. dieffenbachiae	full	full	None
**GCF_017745955.1**	Xanthomonas phaseoli pv. dieffenbachiae	full	full	None
**GCF_017745975.1**	Xanthomonas phaseoli pv. dieffenbachiae	full	full	None
**GCF_017745995.1**	Xanthomonas phaseoli pv. dieffenbachiae	full	full	None
**GCF_017746025.1**	Xanthomonas phaseoli pv. dieffenbachiae	full	full	None
**GCF_017746055.1**	Xanthomonas phaseoli pv. dieffenbachiae	full	full	None
**GCF_017746075.1**	Xanthomonas phaseoli pv. dieffenbachiae	full	full	None
**GCF_017746135.1**	Xanthomonas phaseoli pv. dieffenbachiae	full	full	None
**GCF_017746195.1**	Xanthomonas phaseoli pv. dieffenbachiae	full	full	None
**GCF_017746215.1**	Xanthomonas phaseoli pv. dieffenbachiae	full	full	None

GCF_017746235.1	Xanthomonas phaseoli pv. dieffenbachiae	full	full	None
GCF_017746255.1	Xanthomonas phaseoli pv. dieffenbachiae	full	full	None
GCF_017746275.1	Xanthomonas phaseoli pv. dieffenbachiae	full	full	None
GCF_017746295.1	Xanthomonas phaseoli pv. dieffenbachiae	full	full	None
GCF_017746315.1	Xanthomonas phaseoli pv. dieffenbachiae	full	full	None
GCF_017746335.1	Xanthomonas phaseoli pv. dieffenbachiae	full	full	None
GCF_017746355.1	Xanthomonas phaseoli pv. dieffenbachiae	full	full	None
GCF_017746375.1	Xanthomonas phaseoli pv. dieffenbachiae	full	full	None
GCF_017746395.1	Xanthomonas phaseoli pv. dieffenbachiae	full	full	None
GCF_017746415.1	Xanthomonas phaseoli pv. dieffenbachiae	full	full	None
GCF_017746435.1	Xanthomonas phaseoli pv. dieffenbachiae	full	full	None
GCF_017746455.1	Xanthomonas phaseoli pv. dieffenbachiae	full	full	None
GCF_017746475.1	Xanthomonas phaseoli pv. dieffenbachiae	full	full	None
GCF_017746495.1	Xanthomonas phaseoli pv. dieffenbachiae	full	full	None
GCF_017746515.1	Xanthomonas phaseoli pv. dieffenbachiae	full	full	None
GCF_017746535.1	Xanthomonas phaseoli pv. dieffenbachiae	full	full	None
GCF_017746555.1	Xanthomonas phaseoli pv. dieffenbachiae	full	full	None
GCF_017746575.1	Xanthomonas phaseoli pv. dieffenbachiae	full	full	None
GCF_017746595.1	Xanthomonas phaseoli pv. dieffenbachiae	full	full	None
GCF_017746615.1	Xanthomonas phaseoli pv. dieffenbachiae	full	full	None
GCF_017746635.1	Xanthomonas phaseoli pv. dieffenbachiae	full	full	None
GCF_017746655.1	Xanthomonas phaseoli pv. dieffenbachiae	full	full	None
GCF_017746665.1	Xanthomonas phaseoli pv. dieffenbachiae	full	full	None
GCF_018831305.1	Xanthomonas sp. MLO165	None	None	None
GCF_019192985.1	Xanthomonas euvesicatoria pv. physalidis	full	full	None
GCF_019193005.1	Xanthomonas euvesicatoria pv. alangii	full	None	None
GCF_019201105.1	Xanthomonas campestris pv. obscurae	full	partial	None
GCF_020011215.1	Xanthomonas citri pv. thirumalacharii	full	None	None
GCF_020011255.1	Xanthomonas citri pv. sesbaniae	full	None	None
GCF_020731405.1	Xanthomonas campestris pv. armoraciae	partial	None	None
GCF_020784175.1	Xanthomonas campestris pv. asclepiadis	None	None	None
GCF_903989465.1	Xanthomonas sp. CPBF 426	None	None	None
GCF_000175135.1	Xanthomonas citri pv. aurantifolii str. ICPB 11122	full	None	None
GCF_000175155.1	Xanthomonas citri pv. aurantifolii str. ICPB 10535	full	None	None
GCF_000309905.1	Xanthomonas citri pv. malvacearum str. GSPB1386	full	None	None
GCF_000309925.1	Xanthomonas citri pv. malvacearum str. GSPB2388	full	None	None
GCF_000590235.1	Xanthomonas citri pv. fuscans CFBP 6996	full	None	None
GCF_002837255.1	Xanthomonas citri pv. anacardii	full	partial	None
GCF_002898415.1	Xanthomonas citri pv. anacardii	full	None	None
GCF_002898475.1	Xanthomonas citri pv. anacardii	full	None	None
GCF_000212775.1	Xanthomonas oryzae X8-1A	partial	full	full

**GCF_000507025.1**	Xanthomonas oryzae pv. oryzicola MAI10	partial	full	full
**GCF_000511585.1**	Xanthomonas oryzae pv. oryzae NAI8	partial	full	full
**GCF_000263835.1**	Xanthomonas campestris JX	partial	None	None
**GCF_000321125.2**	Xanthomonas campestris pv. campestris str. Xca5	partial	None	None
**GCF_002019165.1**	Xanthomonas campestris pv. leeana	full	None	None
**GCF_002019195.1**	Xanthomonas campestris pv. thespesiae	full	None	None
**GCF_002019295.1**	Xanthomonas campestris pv. vitistrifoliae	full	None	None
**GCF_002019305.1**	Xanthomonas campestris pv. vitiscarnosae	full	None	None
**GCF_002019335.1**	Xanthomonas campestris pv. vitiswoodrowii	full	None	None
**GCF_002019395.1**	Xanthomonas campestris pv. centellae	full	None	None
**GCF_019201065.1**	Xanthomonas campestris pv. olitorii	full	None	None
**GCF_019201115.1**	Xanthomonas campestris pv. uppalii	full	full	None
**GCF_019201145.1**	Xanthomonas campestris pv. mirabilis	full	full	None
**GCF_019201165.1**	Xanthomonas campestris pv. pennamericanum	full	partial	None
**GCF_019201185.1**	Xanthomonas campestris pv. lawsoniae	full	full	None
**GCF_019201205.1**	Xanthomonas campestris pv. zingibericola	full	full	None
**GCF_019201225.1**	Xanthomonas campestris pv. clerodendri	full	full	None
**GCF_019201245.1**	Xanthomonas campestris pv. convolvuli	full	full	None
**GCF_019201265.1**	Xanthomonas campestris pv. viegasii	full	full	None
**GCF_019201285.1**	Xanthomonas campestris pv. paulliniae	full	None	None
**GCF_019201305.1**	Xanthomonas campestris pv. coriandri	full	full	None
**GCF_021353165.1**	Xanthomonas campestris pv. coriandri	full	full	None
**GCF_019201325.1**	Xanthomonas campestris pv. daturae	full	full	None
**GCF_019201345.1**	Xanthomonas campestris pv. veroniae	full	partial	None
**GCF_019201365.1**	Xanthomonas campestris pv. carissae	full	partial	None
**GCF_019201375.1**	Xanthomonas campestris pv. fici	full	None	None
**GCF_019201405.1**	Xanthomonas campestris pv. heliotropii	partial	full	None
**GCF_019201425.1**	Xanthomonas campestris pv. euphorbiae	full	None	None
**GCF_019201445.1**	Xanthomonas campestris pv. spermacoces	full	full	None
**GCF_019201465.1**	Xanthomonas campestris pv. ionidii	full	partial	None
**GCF_019201485.1**	Xanthomonas campestris pv. blepharidis	full	full	None
**GCF_020783715.1**	Xanthomonas campestris pv. phormiicola	None	None	None
**GCF_020783765.1**	Xanthomonas campestris pv. parthenii	full	None	None
**GCF_020783815.1**	Xanthomonas campestris pv. zinniae	None	None	None
**GCF_020784125.1**	Xanthomonas campestris pv. esculenti	None	None	None
**GCF_900002225.1**	Xanthomonas campestris pv. campestris str. CN11	partial	None	None
**GCF_000306055.1**	Xanthomonas arboricola pv. juglandis str. NCPPB 1447	None	None	None
**GCF_000355635.2**	Xanthomonas arboricola pv. corylina str. NCCB 100457	None	None	None
**GCF_000585435.1**	Xanthomonas arboricola 3004	None	None	None
**GCF_000724915.1**	Xanthomonas arboricola pv. celebensis	None	None	None
**GCF_000724925.1**	Xanthomonas arboricola pv. celebensis	None	None	None
**GCF_002939945.1**	Xanthomonas arboricola pv. populi	None	None	None

**GCF_002940585.1**	Xanthomonas arboricola pv. populi	None	None	None
**GCF_002940145.1**	Xanthomonas arboricola pv. guizotiae	None	None	None
**GCF_002940205.1**	Xanthomonas arboricola pv. guizotiae	None	None	None
**GCF_002940565.1**	Xanthomonas arboricola pv. arracaciae	None	None	None
**GCF_002940625.1**	Xanthomonas arboricola pv. zantedeschiae	None	None	None
**GCF_900240325.1**	Xanthomonas arboricola pv. fragariae	None	None	None
**GCF_900240345.1**	Xanthomonas arboricola pv. fragariae	None	None	None
**GCF_900240355.1**	Xanthomonas arboricola pv. fragariae	None	None	None
**GCF_900240365.1**	Xanthomonas arboricola pv. fragariae	None	None	None
**GCF_900240435.1**	Xanthomonas arboricola pv. fragariae	None	None	None
**GCF_000313775.1**	Xanthomonas translucens pv. graminis ART-Xtg29	None	None	None
**GCF_900092425.1**	Xanthomonas translucens pv. graminis ART-Xtg29	None	None	None
**GCF_000334075.1**	Xanthomonas translucens DAR61454	partial	partial	full
**GCF_001269865.1**	Xanthomonas translucens pv. graminis	None	None	None
**GCF_001659905.1**	Xanthomonas translucens pv. graminis	None	None	None
**GCF_900092365.1**	Xanthomonas translucens pv. graminis	None	None	partial
**GCF_900092415.1**	Xanthomonas translucens pv. graminis	None	None	full
**GCF_900092485.1**	Xanthomonas translucens pv. graminis	None	None	partial
**GCF_900092495.1**	Xanthomonas translucens pv. graminis	None	None	None
**GCF_900092565.1**	Xanthomonas translucens pv. graminis	None	None	partial
**GCF_001282765.1**	Xanthomonas translucens pv. poae	None	None	None
**GCF_001659965.1**	Xanthomonas translucens pv. poae	None	None	None
**GCF_001707285.1**	Xanthomonas translucens pv. poae	None	None	None
**GCF_001707335.1**	Xanthomonas translucens pv. poae	None	None	None
**GCF_001707435.1**	Xanthomonas translucens pv. poae	None	None	None
**GCF_001282805.1**	Xanthomonas translucens pv. arrhenatheri LMG 727	None	None	None
**GCF_001282885.1**	Xanthomonas translucens pv. phlei	None	None	None
**GCF_001401625.1**	Xanthomonas euvesicatoria pv. alfalfae	full	full	None
**GCF_017724035.1**	Xanthomonas euvesicatoria pv. alfalfae	full	full	None
**GCF_001659895.1**	Xanthomonas translucens pv. arrhenatheri	None	None	None
**GCF_002939715.1**	Xanthomonas euvesicatoria pv. citrumelonis	full	full	None
**GCF_005059795.1**	Xanthomonas euvesicatoria pv. citrumelonis	partial	full	None
**GCF_020879155.1**	Xanthomonas euvesicatoria pv. euvesicatoria	full	full	None
**GCF_020879415.1**	Xanthomonas euvesicatoria pv. euvesicatoria	full	full	None
**GCF_020879635.1**	Xanthomonas euvesicatoria pv. euvesicatoria	full	full	None
**GCF_020879655.1**	Xanthomonas euvesicatoria pv. euvesicatoria	full	full	None
**GCF_020879745.1**	Xanthomonas euvesicatoria pv. euvesicatoria	full	full	None
**GCF_020879775.1**	Xanthomonas euvesicatoria pv. euvesicatoria	full	full	None
**GCF_020879795.1**	Xanthomonas euvesicatoria pv. euvesicatoria	full	full	None
**GCF_020879815.1**	Xanthomonas euvesicatoria pv. euvesicatoria	full	full	None
**GCF_020879825.1**	Xanthomonas euvesicatoria pv. euvesicatoria	full	full	None

**GCF_020879845.1**	Xanthomonas euvesicatoria pv. euvesicatoria	full	full	None
**GCF_020879875.1**	Xanthomonas euvesicatoria pv. euvesicatoria	full	full	None
**GCF_020880015.1**	Xanthomonas euvesicatoria pv. euvesicatoria	full	full	None
**GCF_020880135.1**	Xanthomonas euvesicatoria pv. euvesicatoria	full	full	None
**GCF_020880315.1**	Xanthomonas euvesicatoria pv. euvesicatoria	full	full	None
**GCF_020880395.1**	Xanthomonas euvesicatoria pv. euvesicatoria	full	full	None
**GCF_020880575.1**	Xanthomonas euvesicatoria pv. euvesicatoria	full	full	None
**GCF_020880635.1**	Xanthomonas euvesicatoria pv. euvesicatoria	full	full	None
**GCF_020880835.1**	Xanthomonas euvesicatoria pv. euvesicatoria	full	full	None
**GCF_020880855.1**	Xanthomonas euvesicatoria pv. euvesicatoria	full	full	None
**GCF_020880895.1**	Xanthomonas euvesicatoria pv. euvesicatoria	full	full	None
**GCF_020879135.1**	Xanthomonas euvesicatoria pv. euvesicatoria	full	full	None
**GCF_020879265.1**	Xanthomonas euvesicatoria pv. euvesicatoria	full	full	None
**GCF_020879485.1**	Xanthomonas euvesicatoria pv. euvesicatoria	full	full	None
**GCF_020880035.1**	Xanthomonas euvesicatoria pv. euvesicatoria	full	full	None
**GCF_020880045.1**	Xanthomonas euvesicatoria pv. euvesicatoria	full	full	None
**GCF_020880115.1**	Xanthomonas euvesicatoria pv. euvesicatoria	full	full	None
**GCF_020880165.1**	Xanthomonas euvesicatoria pv. euvesicatoria	full	full	None
**GCF_020880235.1**	Xanthomonas euvesicatoria pv. euvesicatoria	full	full	None
**GCF_020880275.1**	Xanthomonas euvesicatoria pv. euvesicatoria	full	full	None
**GCF_020880345.1**	Xanthomonas euvesicatoria pv. euvesicatoria	full	full	None
**GCF_020880375.1**	Xanthomonas euvesicatoria pv. euvesicatoria	full	full	None
**GCF_020880415.1**	Xanthomonas euvesicatoria pv. euvesicatoria	full	full	None
**GCF_020880435.1**	Xanthomonas euvesicatoria pv. euvesicatoria	full	full	None
**GCF_000265585.1**	Xanthomonas axonopodis pv. manihotis str. UA226	full	None	None
**GCF_000265725.1**	Xanthomonas axonopodis pv. manihotis str. IBSBF 356	full	None	None
**GCF_000265745.1**	Xanthomonas axonopodis pv. manihotis str. IBSBF 320	full	None	None
**GCF_000265805.1**	Xanthomonas axonopodis pv. manihotis str. CFBP1851	full	None	None
**GCF_000265825.1**	Xanthomonas axonopodis pv. manihotis str. CIO1	full	None	None
**GCF_000265845.1**	Xanthomonas axonopodis pv. manihotis str. CIO151	full	None	None
**GCF_000265885.1**	Xanthomonas axonopodis pv. manihotis str. IBSBF 1411	full	None	None
**GCF_000265925.1**	Xanthomonas axonopodis pv. manihotis str. IBSBF 2345	full	None	None
**GCF_000265945.1**	Xanthomonas axonopodis pv. manihotis str. IBSBF 2346	full	None	None
**GCF_000266005.1**	Xanthomonas axonopodis pv. manihotis str. IBSBF 2672	full	None	None
**GCF_000266285.1**	Xanthomonas axonopodis pv. manihotis str. NCPPB 1159	full	None	None
**GCF_000266505.1**	Xanthomonas axonopodis pv. manihotis str. UG27	full	None	None
**GCF_000588855.1**	Xanthomonas phaseoli pv. phaseoli CFBP 6546	full	None	None
**GCF_000265565.1**	Xanthomonas axonopodis pv. manihotis str. UA303	full	None	None
**GCF_000265605.1**	Xanthomonas axonopodis pv. manihotis str. ORST17	full	None	None

**GCF_000265625.1**	Xanthomonas axonopodis pv. manihotis str. IBSBF 2666	full	None	None
**GCF_000265645.1**	Xanthomonas axonopodis pv. manihotis str. IBSBF 2665	full	None	None
**GCF_000265665.1**	Xanthomonas axonopodis pv. manihotis str. IBSBF 321	partial	None	None
**GCF_000265685.1**	Xanthomonas axonopodis pv. manihotis str. IBSBF 436	full	None	None
**GCF_000265705.1**	Xanthomonas axonopodis pv. manihotis str. NG1	partial	None	None
**GCF_000265765.1**	Xanthomonas axonopodis pv. manihotis str. AT6B	full	None	None
**GCF_000265785.1**	Xanthomonas axonopodis pv. manihotis str. AFNC1360	full	None	None
**GCF_000265865.1**	Xanthomonas axonopodis pv. manihotis str. IBSBF 1182	partial	partial	None
**GCF_000265905.1**	Xanthomonas axonopodis pv. manihotis str. IBSBF 1994	full	None	None
**GCF_000265965.1**	Xanthomonas axonopodis pv. manihotis str. IBSBF 2538	full	None	None
**GCF_000265985.1**	Xanthomonas axonopodis pv. manihotis str. IBSBF 2539	full	None	None
**GCF_000266025.1**	Xanthomonas axonopodis pv. manihotis str. IBSBF 2673	full	None	None
**GCF_000266045.1**	Xanthomonas axonopodis pv. manihotis str. IBSBF 278	full	None	None
**GCF_000266065.1**	Xanthomonas axonopodis pv. manihotis str. IBSBF 280	partial	partial	None
**GCF_000266085.1**	Xanthomonas axonopodis pv. manihotis str. IBSBF 2816	full	None	None
**GCF_000266105.2**	Xanthomonas axonopodis pv. manihotis str. IBSBF 2818	full	None	None
**GCF_000266125.1**	Xanthomonas axonopodis pv. manihotis str. IBSBF 2819	full	None	None
**GCF_000266145.1**	Xanthomonas axonopodis pv. manihotis str. IBSBF 2820	full	None	None
**GCF_000266165.1**	Xanthomonas axonopodis pv. manihotis str. IBSBF 2821	full	None	None
**GCF_000266185.1**	Xanthomonas axonopodis pv. manihotis str. IBSBF 2822	full	None	None
**GCF_000266205.1**	Xanthomonas axonopodis pv. manihotis str. IBSBF 285	full	None	None
**GCF_000266225.1**	Xanthomonas axonopodis pv. manihotis str. IBSBF 289	full	None	None
**GCF_000266245.1**	Xanthomonas axonopodis pv. manihotis str. IBSBF 725	full	None	None
**GCF_000266265.1**	Xanthomonas axonopodis pv. manihotis str. IBSBF 726	full	None	None
**GCF_000266305.1**	Xanthomonas axonopodis pv. manihotis str. ORST4	partial	partial	None
**GCF_000266325.1**	Xanthomonas axonopodis pv. manihotis str. ORST X27	full	None	None
**GCF_000266345.1**	Xanthomonas axonopodis pv. manihotis str. ThaiXam	full	None	None
**GCF_000266365.1**	Xanthomonas axonopodis pv. manihotis str. UA536	full	None	None
**GCF_000266385.1**	Xanthomonas axonopodis pv. manihotis str. UA556	full	None	None
**GCF_000266405.1**	Xanthomonas axonopodis pv. manihotis str. UA560	full	None	None
**GCF_000266425.1**	Xanthomonas axonopodis pv. manihotis str. UA686	full	None	None
**GCF_000266445.1**	Xanthomonas axonopodis pv. manihotis str. UG21	full	None	None
**GCF_000266465.1**	Xanthomonas axonopodis pv. manihotis str. UG23	full	None	None
**GCF_000266485.1**	Xanthomonas axonopodis pv. manihotis str. UG24	full	None	None

**GCF_000266525.1**	Xanthomonas axonopodis pv. manihotis str. UG28	full	None	None
**GCF_000266545.1**	Xanthomonas axonopodis pv. manihotis str. UG39	full	None	None
**GCF_000266565.1**	Xanthomonas axonopodis pv. manihotis str. UG43	full	None	None
**GCF_000266585.1**	Xanthomonas axonopodis pv. manihotis str. UG44	full	None	None
**GCF_000266605.1**	Xanthomonas axonopodis pv. manihotis str. UG45	full	None	None
**GCF_000266625.1**	Xanthomonas axonopodis pv. manihotis str. UG51	full	None	None
**GCF_000266645.1**	Xanthomonas axonopodis pv. manihotis str. Xam1134	full	None	None
**GCF_000266665.1**	Xanthomonas axonopodis pv. manihotis str. Xam668	full	None	None
**GCF_000266685.1**	Xanthomonas axonopodis pv. manihotis str. Xam669	full	None	None
**GCF_000266705.1**	Xanthomonas axonopodis pv. manihotis str. Xam672	full	None	None
**GCF_000266725.1**	Xanthomonas axonopodis pv. manihotis str. Xam678	full	None	None
**GCF_000266745.1**	Xanthomonas axonopodis pv. manihotis str. IBSBF 2667	full	None	None
**GCF_000266765.1**	Xanthomonas axonopodis pv. manihotis str. IBSBF 2670	full	None	None
**GCF_000266785.1**	Xanthomonas axonopodis pv. manihotis str. IBSBF 614	full	None	None
**GCF_000266805.1**	Xanthomonas axonopodis pv. manihotis str. UA323	full	None	None
**GCF_000266825.1**	Xanthomonas axonopodis pv. manihotis str. UA306	full	None	None
**GCF_000266845.1**	Xanthomonas axonopodis pv. manihotis str. UA324	full	None	None
**GCF_020879615.1**	Xanthomonas phaseoli	full	None	None
**GCF_020880215.1**	Xanthomonas phaseoli	full	None	None
**GCF_000278055.2**	Xanthomonas vasicola pv. vasculorum NCPPB 890	partial	full	None
**GCF_001189905.1**	Xanthomonas campestris pv. musacearum NCPPB 2251	partial	full	None
**GCF_002019215.1**	Xanthomonas axonopodis pv. melhusii	full	None	None
**GCF_002019345.1**	Xanthomonas axonopodis pv. clitoriae	full	None	None
**GCF_002019375.1**	Xanthomonas axonopodis pv. martyniicola	full	None	None
**GCF_002940645.1**	Xanthomonas axonopodis pv. begoniae	full	None	None
**GCF_003111865.1**	Xanthomonas vasicola pv. zeae	partial	full	None
**GCF_003111965.1**	Xanthomonas vasicola pv. zeae	partial	full	None
**GCF_003112025.1**	Xanthomonas vasicola pv. zeae	partial	full	None
**GCF_003112065.1**	Xanthomonas vasicola pv. zeae	partial	full	None
**GCF_003112105.1**	Xanthomonas vasicola pv. zeae	partial	full	None
**GCF_012844535.1**	Xanthomonas vasicola pv. zeae	partial	full	None
**GCF_012844555.1**	Xanthomonas vasicola pv. zeae	partial	full	None
**GCF_012844575.1**	Xanthomonas vasicola pv. zeae	partial	full	None
**GCF_012844585.1**	Xanthomonas vasicola pv. zeae	partial	full	None
**GCF_012844655.1**	Xanthomonas vasicola pv. zeae	partial	full	None
**GCF_012844665.1**	Xanthomonas vasicola pv. zeae	partial	full	None
**GCF_012844675.1**	Xanthomonas vasicola pv. zeae	partial	full	None
**GCF_012844705.1**	Xanthomonas vasicola pv. zeae	partial	full	None
**GCF_003111985.1**	Xanthomonas vasicola pv. zeae	partial	full	None
**GCF_003112005.1**	Xanthomonas vasicola pv. zeae	partial	full	None

**GCF_012844565.1**	Xanthomonas vasicola pv. zeae	partial	full	None
**GCF_012844625.1**	Xanthomonas vasicola pv. zeae	partial	full	None
**GCF_003932615.1**	Xanthomonas vasicola pv. musacearum	partial	full	None
**GCF_019209705.1**	Xanthomonas vasicola pv. musacearum	partial	full	None
**GCF_019209735.1**	Xanthomonas vasicola pv. musacearum	partial	full	None
**GCF_003862455.1**	Xanthomonas vasicola pv. musacearum	partial	full	None
**GCF_003957475.1**	Xanthomonas axonopodis pv. eucalyptorum	full	None	partial
**GCF_012922215.1**	Xanthomonas hortorum pv. pelargonii	None	None	None
**GCF_021353095.1**	Xanthomonas hortorum pv. pelargonii	None	None	None
**GCF_903978255.1**	Xanthomonas hortorum pv. carotae	None	None	None
**GCF_000159795.2**	Xanthomonas vasicola pv. vasculorum NCPPB 702	partial	partial	None
**GCF_000277875.1**	Xanthomonas campestris pv. musacearum NCPPB 2005	partial	full	None
**GCF_000277955.1**	Xanthomonas campestris pv. musacearum NCPPB 4392	partial	full	None
**GCF_000277975.1**	Xanthomonas campestris pv. musacearum NCPPB 4394	partial	full	None
**GCF_000277995.1**	Xanthomonas vasicola pv. vasculorum NCPPB 1326	partial	full	None
**GCF_000278015.1**	Xanthomonas vasicola pv. vasculorum NCPPB 1381	partial	full	None
**GCF_000278035.1**	Xanthomonas vasicola pv. vasculorum NCPPB 206	partial	full	None
**GCF_000278075.1**	Xanthomonas vasicola pv. vasculorum NCPPB 895	partial	partial	None
**GCF_002019095.1**	Xanthomonas axonopodis pv. bauhiniae	full	None	None
**GCF_002019105.1**	Xanthomonas axonopodis pv. cajani	full	None	None
**GCF_002019155.1**	Xanthomonas axonopodis pv. khayae	full	None	None
**GCF_012922225.1**	Xanthomonas hortorum pv. taraxaci	None	None	None
**GCF_021352955.1**	Xanthomonas hortorum pv. taraxaci	None	None	None
**GCF_000225975.1**	Xanthomonas sacchari NCPPB 4393	None	None	None
**GCF_000376745.1**	Xanthomonas fragariae LMG 25863	None	None	full
**GCF_000802365.1**	Xanthomonas cannabis pv. cannabis	None	None	None
**GCF_000802405.1**	Xanthomonas cannabis pv. cannabis	None	None	None
**GCF_001423495.1**	Xanthomonas sp. Leaf131	full	None	None
**GCF_001423585.1**	Xanthomonas sp. Leaf148	full	None	None
**GCF_001855615.1**	Xanthomonas alfalfae	partial	partial	None
**GCF_002940185.1**	Xanthomonas sp. CFBP 7912	None	None	None
**GCF_003600855.1**	Xanthomonas sp. CFBP 7698	None	None	None
**GCF_003993515.1**	Xanthomonas sp. BRIP62415	None	None	None
**GCF_003993525.1**	Xanthomonas sp. BRIP62411	None	None	None
**GCF_011927485.1**	Xanthomonas sp. 3376	None	None	None
**GCF_011927495.1**	Xanthomonas sp. 3272	None	None	None
**GCF_013410075.1**	Xanthomonas sp. JAI131	full	None	None
**GCF_014199795.1**	Xanthomonas sp. 3498	None	None	None
**GCF_014851915.1**	Xanthomonas sp. XNM01	None	None	None

**GCF_020515615.1**	Xanthomonas sp. MWU16_30325	None	None	None
**GCF_020880755.1**	Xanthomonas cannabis	None	None	None
**GCF_902375895.1**	Xanthomonas massiliensis	None	None	full
**GCF_900018785.1**	Xanthomonas massiliensis	None	None	full
**GCF_000364645.1**	Xanthomonas sp. SHU 308	None	None	None
**GCF_000364665.1**	Xanthomonas sp. SHU 199	None	None	None
**GCF_000364685.1**	Xanthomonas sp. SHU 166	None	None	None
**GCF_000963005.1**	Xanthomonas sp. GPE 39	None	None	None
**GCF_000963215.1**	Xanthomonas sp. MUS 060	None	None	None
**GCF_001043115.1**	Xanthomonas sp. NCPPB 1128	None	None	None
**GCF_003992815.1**	Xanthomonas sp. DAR33341	full	None	None
**GCF_003992965.1**	Xanthomonas sp. BRIP62409	None	None	None
**GCF_003993495.1**	Xanthomonas sp. BRIP62418	None	None	None
**GCF_009192885.1**	Xanthomonas sp. LMG 12462	None	None	None
**GCF_009192895.1**	Xanthomonas sp. LMG 12460	None	None	None
**GCF_009192905.1**	Xanthomonas sp. LMG 12459	None	None	None
**GCF_009192935.1**	Xanthomonas sp. LMG 12461	None	None	None
**GCF_009834995.1**	Xanthomonas sp. LMG 8989	None	None	None
**GCF_009835065.1**	Xanthomonas sp. LMG 8993	None	None	None
**GCF_009835085.1**	Xanthomonas sp. LMG 9002	None	None	None
**GCF_009835095.1**	Xanthomonas sp. LMG 8992	None	None	None
**GCF_014836395.1**	Xanthomonas sp. Sa3BUA13	full	None	None
**GCF_017163695.1**	Xanthomonas sp. CFBP 8700	full	None	None
**GCF_017163705.1**	Xanthomonas sp. CFBP 8703	full	None	None
**GCF_017163755.1**	Xanthomonas sp. AmX2	full	partial	full
**GCF_017745875.1**	Xanthomonas sp. A2111	None	None	None
**GCF_017746015.1**	Xanthomonas sp. A1809	full	full	None
**GCF_017746095.1**	Xanthomonas sp. D-109	None	None	None
**GCF_017746115.1**	Xanthomonas sp. D-93	None	None	None
**GCF_017746145.1**	Xanthomonas sp. D99	full	None	None
**GCF_017746155.1**	Xanthomonas sp. D-36-1	full	None	None
**GCF_020783675.1**	Xanthomonas dyei pv. eucalypti	partial	partial	None
**GCF_020783795.1**	Xanthomonas sp. NCPPB 1067	full	None	None
**GCF_900143175.1**	Xanthomonas retroflexus	None	None	None

Supplementary Table 2. List of nonpathogenic strains used in the phylogenetic analysis

	Strain Name	Isolation
1	*Xanthomonas campestris* CFBP7622	Bean leaf washings
2	*Xanthomonas arboricola* 2955	Rain
3	*Xanthomonas arboricola* 3640	Rain
4	*Xanthomonas campestris* CFBP7635	*Juglans regia* cv. Franquette
5	*Xanthomonas arboricola* 2974	Rain
6	*Xanthomonas arboricola* 2949	Rain
7	*Xanthomonas campestris* 3338	Rain
8	*Xanthomonas* sp. BRIP62411	*Solanum lycopersicum*
9	*Xanthomonas* sp. BRIP62409	*Solanum lycopersicum*
10	*Xanthomonas* sp. BRIP62415	*Solanum lycopersicum*
11	*Xanthomonas arboricola* 2957	Rain
12	*Xanthomonas arboricola* F2	*Physalis peruviana* Ground cherry
13	*Xanthomonas arboricola* CFBP8152	*Phaseolus vulgaris*
14	*Xanthomonas campestris* CFBP8151	bean seed
15	*Xanthomonas arboricola* 3793	Rain
16	*Xanthomonas arboricola* 4461	Rain
17	*Xanthomonas arboricola* 3058	Rain
18	*Xanthomonas campestris* 3075	Rain
19	*Xanthomonas* sp. 60	*Solanum lycopersicum*
20	*Xanthomonas* sp. F1	citrus orange
21	*Xanthomonas* sp. 3498	Rain
22	*Xanthomonas* sp. F4	citrus orange
23	*Xanthomonas sacchari* F10	citrus orange
24	*Xanthomonas arboricola* 3307	Rain
25	*Xanthomonas translucens* F5	pepper-Bell Boy
26	*Xanthomonas maliensis* LMG27592	Rice
27	*Xanthomonas maliensis* M97	Rice
28	*Xanthomonas floridensis* WHRI 8848	watercress
29	*Xanthomonas sontii* PPL1	Rice

Supplementary Table 3. Comparison of T6SS i3* cluster core genes in *Xe85-10* and early branching species, *X. translucens* F5 and identifying the closely related species to *X. translucens* F5 i3* cluster

Xe85-10 i3* locus tag	Gene name	Percent identity to Xe85-10 (%)	*X. translucens* F5 i3* cluster Locus tag	Closest match-percent identities (%) according to BLAST results
XCV4202	*tssA*	66.67	Ga0372549_3941	99% identity to *Xanthomonas* sp. SI

XCV4209	*tssM*	68.81	Ga0372549_3934	100% identity to *Xanthomonas* sp. SI
XCV4210	*tssL*	71.93	Ga0372549_3933	95.3% identity to *Xanthomonas* sp. SI
XCV4211	*tssK*	80.18	Ga0372549_3932	96% identity to *Xanthomonas* sp. SI
XCV4217	*vgrG*	61.56	Ga0372549_3929	95% *Xanthomonas* sp. SS
XCV4236	*clpV*	84.55	Ga0372549_3921	95% *Xanthomonas* sp. SS
XCV4237	*tssG*	72.59	Ga0372549_3920	95.45% *Xanthomonas* sp. GW
XCV4238	*tssF*	79.30	Ga0372549_3919	96.04% *Xanthomonas* sp. GW
XCV4239	*tssE*	78.44	Ga0372549_3918	97% *Xanthomonas* sp. SS
XCV4241	*hcp*	97.01	Ga0372549_3916	97.62% *Xanthomonas* sp. SI
XCV4242	*tssC*	93.16	Ga0372549_3915	100% *Xanthomonas* sp. SI
XCV4243	*tssB*	93.45	Ga0372549_3914	97.24% *Xanthomonas* sp. GW
